# Palladium-catalyzed intramolecular dearomatization of indoles *via* Heck/Stille cross-coupling with organostannanes

**DOI:** 10.1039/d6ra02185a

**Published:** 2026-04-30

**Authors:** Zhenjie Qi, Jiajing Hong, Baojing Ji, Chen Chen, Yixuan Xiao, Boxuan Song, Yanguo Lv, Zhenyu An, Peipei Ma

**Affiliations:** a School of Resource & Environment and Safety Engineering, Jining University Qufu 273155 Shandong China 202310001@jnxy.edu.cn; b School of Pharmacy, Ningxia Medical University Yinchuan 750000 Ningxia China

## Abstract

A palladium-catalyzed tandem intramolecular Heck cyclization/intermolecular Stille coupling for the dearomatization of *N*-(2-bromobenzoyl)indoles is described. This organotin-based protocol provides fused indoline derivatives bearing contiguous quaternary carbon stereocenters at the C2 and C3 positions in moderate to excellent yields and is compatible with structurally diverse organotin reagents, including aryl-, naphthyl-, vinyl ether-, alkenyl-, and alkyl-substituted stannanes. Moreover, this protocol is characterized by mild conditions and gram-scale scalability.

## Abstract 2

Palladium-catalyzed dearomative cascade Heck/Stille coupling of *N*-(2-bromobenzoyl)indoles with organostannanes constructs polycyclic indolines with quaternary stereocenters at C2 and C3.

## Graphical abstract

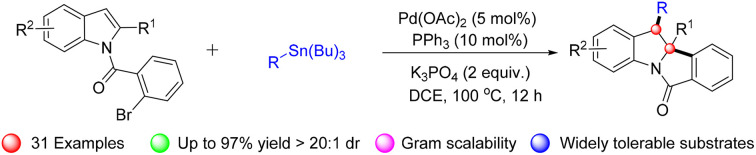

In recent years, transition-metal-catalyzed dearomatization reactions have emerged as a powerful tool for the efficient construction of a diverse array of three-dimensional complex molecules bearing multiple chiral centers. This strategy has markedly enhanced synthetic efficiency and thus provided a pivotal approach for the synthesis of pharmaceuticals, natural products, and the development of functional materials.^1^ Among such compounds, polycyclic fused indolines containing quaternary carbon stereocenters possess rigid three-dimensional architectures and unique electronic properties originating from their fused-ring skeletons. These scaffolds not only serve as the core structural motifs of numerous natural alkaloids and pharmaceutical molecules but also act as important precursors for the fabrication of chiral ligands and functional materials.^2^ Representative examples include (−)-isatisine A,^3^ (±)-mersicarpine,^4^ gliocladin C,^5^ brevianamide E^6^ and strychnine (Fig. 1).^7^ Recently, the mainstream strategy for synthesizing tetracyclic fused indolines relies on the intramolecular dearomative Heck reaction of *N*-(2-halobenzoyl)indoles. This transformation generates a key benzyl-palladium intermediate, which can either be trapped by a diverse array of nucleophiles including hydrides, cyanides, organoboron reagents, alkynes, and azoles or undergo transformation *via* β-hydride elimination.^8^

**Fig. 1:**
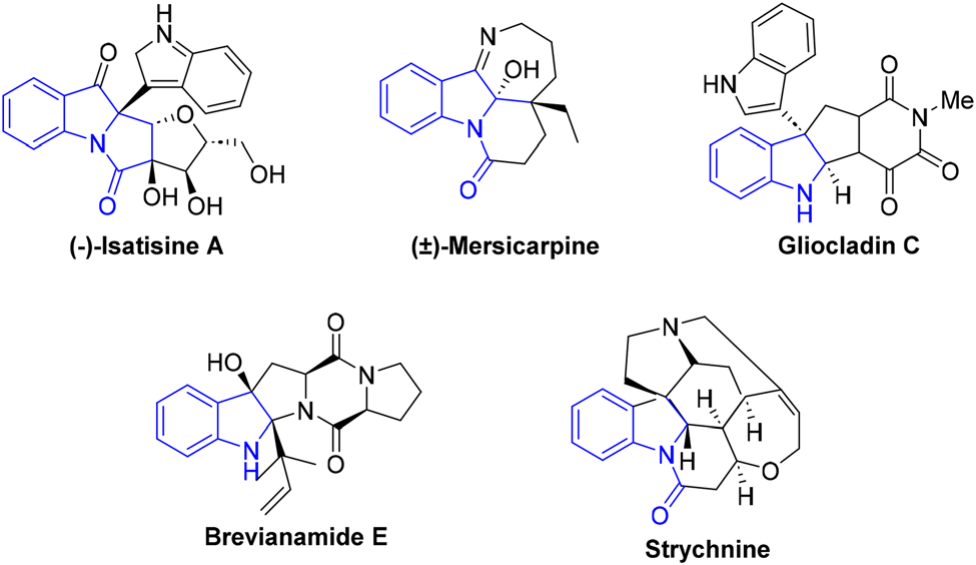
Some representative natural products and bioactive molecules.

Nevertheless, synthetic methodologies for the preparation of C3-aryl-substituted tetracyclic fused indolines remain scarce, with existing approaches primarily focused on the Suzuki coupling of *N*-substituted indoles with arylboronic acids or arylboroxines.^9^ In 2016, Lautens and co-workers reported a palladium-catalyzed dearomative 1,2-diarylation of *N*-(2-bromobenzoyl)indoles using triphenylboroxine as the coupling partner, which successfully afforded C3-aryl-substituted polycyclic fused indolines (Scheme 1a).^9*c*^ In 2021, Zhao's group developed a palladium-catalyzed asymmetric dearomative cyclization of indoles with phenylboronic acids, enabling the stereoselective construction of 3-arylindoline derivatives.^9*a*^ In the same year, Liang's group adopted a similar strategy for the synthesis of tetracyclic indoline frameworks (Scheme 1b).^9*b*^ Inspired by these pioneering studies, herein we report a one-pot protocol for the construction of C3-aryl-substituted polycyclic fused indolines. This methodology is based on the palladium-catalyzed dearomatization of *N*-substituted indoles with stable and readily accessible organotin reagents as coupling partners, proceeding *via* a sequential Heck coupling/Stille coupling cascade process.

**Scheme 1:**
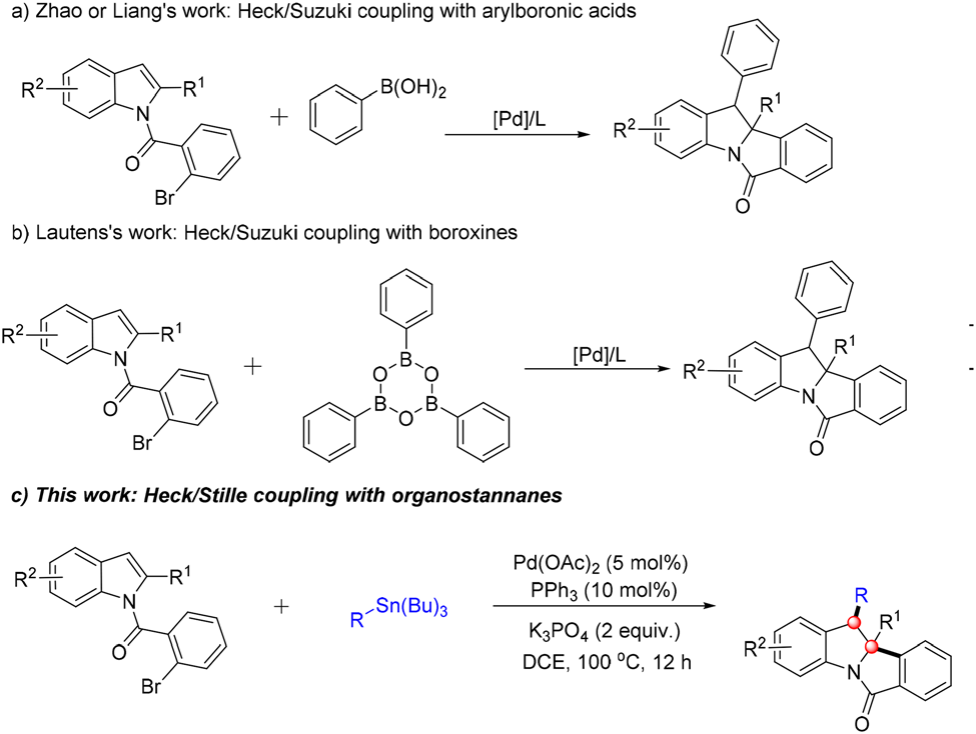
Approaches to 3-aryl-substituted indoline derivatives *via* palladium-catalyzed dearomatization.

Initially, *N*-(2-bromobenzoyl)-2-methylindole (1a) and tributyl(phenyl)stannane (2a) were selected as model substrates for the sequential Heck/Stille cross-coupling reaction, and the transformation was investigated under a palladium/ligand catalytic system (Table 1). The dearomatization reaction was first conducted with Pd(OAc)_2_/PCy_3_·HBF_4_ as the catalytic system, K_2_CO_3_ as the base, in 1,2-dichloroethane (DCE) at 100 °C under nitrogen protection for 12 h, affording the target product 3aa in 55% yield (Table 1, entry 1). The structure of 3aa was unambiguously confirmed by single-crystal X-ray diffraction, providing a solid foundation for subsequent optimization and scope studies. To improve the reaction yield, a systematic series of optimization experiments was subsequently performed. First, various organic solvents were screened, including THF, DCM, 1,4-dioxane and toluene, yet none delivered a superior yield to DCE (Table 1, entries 2–5). Next, a range of phosphine ligands was evaluated to enhance catalytic activity. When PtBu_3_·HBF_4_, PPh_3_, DPPB and XPhos were tested individually, the use of PPh_3_ as the ligand led to a slight increase in yield, affording 3aa in 66% yield (Table 1, entries 6–9). We then focused on the screening of inorganic bases, given that the base plays a pivotal role in modulating reaction kinetics and product formation. The results demonstrated that K_3_PO_4_ outperformed K_2_CO_3_ significantly, furnishing 3aa in an excellent yield of 87% (Table 1, entry 12). In contrast, other bases including Na_2_CO_3_, NaHCO_3_, NaOAc, and Na_2_HPO_4_ exhibited poorer efficacy in promoting this transformation (Table 1, entries 10, 11 and 13 and 14). Finally, a variety of palladium precatalysts, including PdCl_2_(CH_3_CN)_2_ and Pd(dba)_2_, were screened to identify the optimal catalytic precursor. Among these, Pd(OAc)_2_ remained the optimal choice, as the other palladium sources resulted in a notable decrease in yield (Table 1, entries 15 and 16). On the basis of the above optimization studies, the optimal reaction conditions were established as follows: 5 mol% Pd(OAc)_2_ as the precatalyst, 10 mol% PPh_3_ as the ligand, 2.0 equiv. K_3_PO_4_ as the base, and DCE as the solvent, with stirring at 100 °C under an N_2_ atmosphere for 12 h (Table 1, entry 12).

**Table 1:** Optimization of reaction conditions^a^

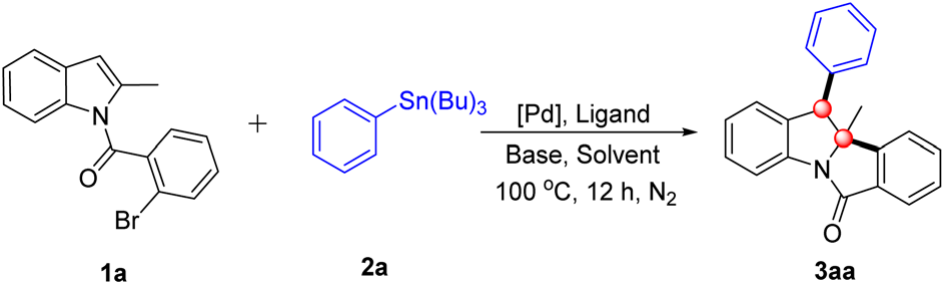					
Entry	[Pd]	Ligand	Base	Solvent	Yield^b^ %
1	Pd(OAc)_2_	PCy_3_·HBF_4_	K_2_CO_3_	DCE	55
2	Pd(OAc)_2_	PCy_3_·HBF_4_	K_2_CO_3_	THF	25
3	Pd(OAc)_2_	PCy_3_·HBF_4_	K_2_CO_3_	DCM	43
4	Pd(OAc)_2_	PCy_3_·HBF_4_	K_2_CO_3_	Dioxane	36
5	Pd(OAc)_2_	PCy_3_·HBF_4_	K_2_CO_3_	Toluene	30
6	Pd(OAc)_2_	P^*t*^Bu_3_·HBF_4_	K_2_CO_3_	DCE	45
7	Pd(OAc)_2_	PPh_3_	K_2_CO_3_	DCE	66
8	Pd(OAc)_2_	DPPB	K_2_CO_3_	DCE	40
9	Pd(OAc)_2_	XPhos	K_2_CO_3_	DCE	28
10	Pd(OAc)_2_	PPh_3_	Na_2_CO_3_	DCE	60
11	Pd(OAc)_2_	PPh_3_	NaHCO_3_	DCE	59
**12**	**Pd(OAc)**_**2**_	**PPh**_**3**_	**K**_**3**_ **PO**_**4**_	**DCE**	**87**
13	Pd(OAc)_2_	PPh_3_	NaOAc	DCE	45
14	Pd(OAc)_2_	PPh_3_	Na_2_HPO_4_	DCE	68
15	PdCl_2_(CH_3_CN)_2_	PPh_3_	K_3_PO_4_	DCE	60
16	Pd(dba)_2_	PPh_3_	K_3_PO_4_	DCE	59

a Reaction conditions: 1a (0.2 mmol), 2a (0.3 mmol), [Pd] (5 mol%), ligand (10 mol%), base (2.0 equiv.) and solvent (4.0 mL), at 100 °C under N_2_ atmosphere for 12 h.

b Isolated yield.

With the optimized conditions established, we further explored the substrate scope of this dearomative cascade transformation. We first evaluated *N*-(2-bromobenzoyl)-2-methylindole (1a) and its ring-substituted derivatives, results summarized in Table 2. Indole substrates with various C2 substituents (alkyl/aryl groups) were also compatible, delivering 3aa–3pa in moderate to good yields. It is noteworthy that substrates bearing an aryl group at the C2 position of the indole ring generally afforded lower yields (50–79%) compared to the methyl-substituted analogue 1a (87%). This is due to the larger size of the aryl group and altered electronic effects, which impede the key Heck insertion step by increasing steric congestion around the palladium center. Unsubstituted C2 afforded product 3pa only in 36% yield, much lower than substituted analogues. This is presumably due to the unsubstituted indole's propensity for Heck homocoupling side reactions, reducing the desired product yield. C2 alkyl-substituted indoles (*e.g.*, cyclopropyl) afforded 3ba in 60% yield. Replacement of C2–H with a phenyl group (either EWG- or EDG-substituted) enabled smooth reaction (3ca–3ma). Steric hindrance was negligible, with moderate yields maintained for *ortho*-, *meta*-, and *para*-substituted phenyl groups. C2 substituents such as 2-naphthyl and 2-thienyl (heteroaromatic) also gave good yields (3na–3oa), confirming the catalytic system's broad substrate tolerance. The reaction proceeded smoothly when C5/C6 positions of the indole ring bore diverse functional groups, affording products 3qa–3ua in 62–97% yields. A distinct electronic trend was observed: the electron-donating group (EDG)-substituted substrate 3ra gave an excellent 97% yield, significantly higher than those with electron-withdrawing groups (EWGs) (3sa–3ua).

**Table 2:** Substrate scope of substituted isoindolo[2,1-*a*]indol-6-one^a^

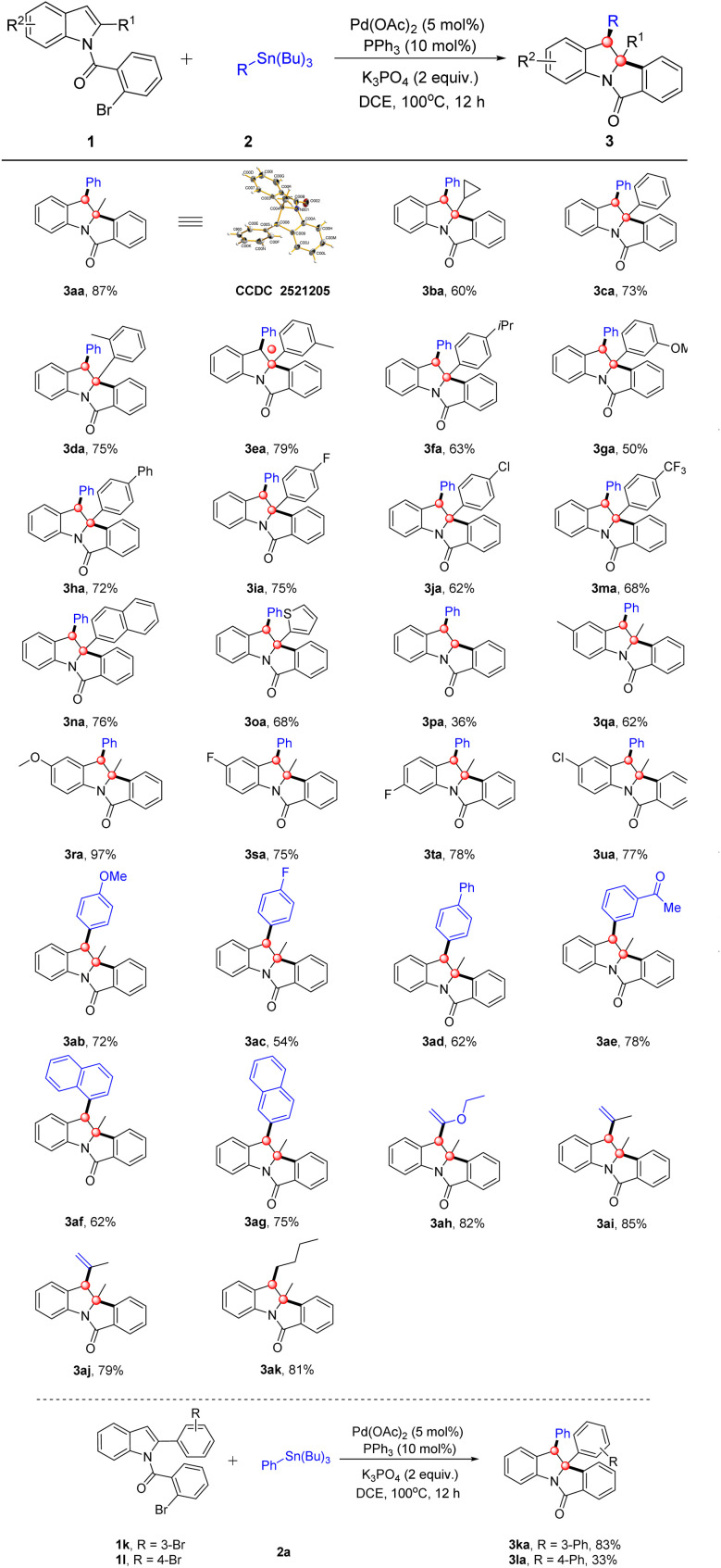

a Reaction conditions: 1 (0.2 mmol), 2 (0.3 mmol), [Pd] (5 mol%), ligand (10 mol%), base (2.0 equiv.) and solvent (4.0 mL), at 100 °C under N_2_ atmosphere for 12 h.

We further extended the study to the scope of organotin reagents with indoles (Table 2). Phenyl-substituted organotin substrates bearing EDGs (methoxy), EWGs (fluoro, formyl) or a phenyl group all reacted smoothly, affording 3ab–3ae in 54–78% yields. Gratifyingly, replacing the phenyl group with 1-naphthyl or 2-naphthyl also enabled efficient transformation, giving the products 3af–3ag in 62% and 75% yields, respectively. Notably, vinyl ether-, alkenyl-, and alkyl-substituted organotin reagents were likewise competent coupling partners, furnishing 3ah–3ak in 79–85% yields. These results further expand the catalytic system's applicability to structurally diverse organotin reagents. The relative configuration of the C2 and C3 quaternary stereocenters was established as *cis* by X-ray crystallography of 3aa, and all other products were assigned as single diastereomers with the same relative configuration on the basis of the rigid fused-ring system.

To demonstrate the scalability of this palladium-catalyzed dearomatization reaction, a gram-scale experiment was conducted (Scheme 2).^10^ Product 3aa was scaled up in 10.0 mL DCE at 100 °C for 15 h, using the standard catalytic system (5 mol% Pd(OAc)_2_, 10 mol% PPh_3_, 2.0 equiv. K_3_PO_4_). Gratifyingly, the desired product 3aa was isolated in a synthetically useful 74% yield, verifying the transformation's practical applicability.

**Scheme 2:**
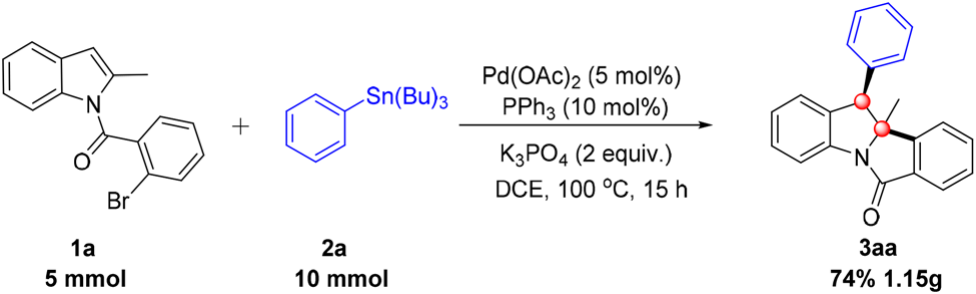
Gram-scale reaction.

Based on our experimental results and published literature, a plausible catalytic cycle for the palladium-catalyzed dearomative cyclization reaction is proposed in Scheme 3.^11^ Pd(0) species are *in situ* generated from Pd(OAc)_2_ and PPh_3_ ligands, which undergo oxidative addition with the C–Br bond of aryl bromide 1a to form arylpalladium complex A. Subsequent coordination of the indole C2=C3 bond to the palladium center, followed by intramolecular migratory insertion, furnishes dearomatized alkylpalladium(ii) intermediate C*via* transition state B. Intermediate C then undergoes transmetalation with tributylphenylstannane (2a) to form an alkyl(aryl)palladium(ii) intermediate D. Finally, reductive elimination of D yields target product 3aa and releases Pd(0) to perpetuate the catalytic cycle.

**Scheme 3:**
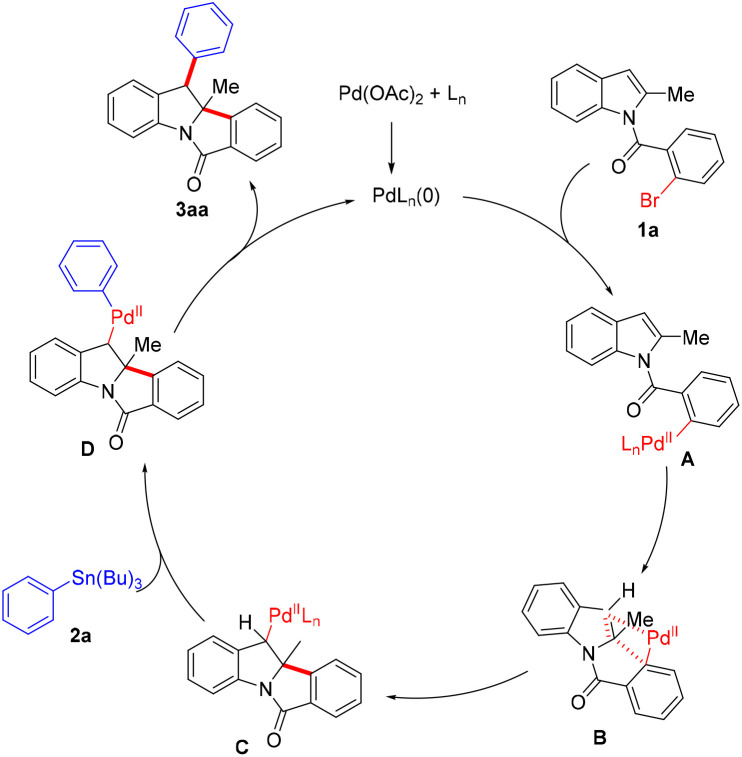
Proposed reaction mechanism.

In summary, we have developed a palladium-catalyzed tandem intramolecular Heck cyclization/intermolecular Stille coupling for the dearomatization of *N*-(2-bromobenzoyl)indoles. This method provides fused indoline frameworks bearing contiguous quaternary carbon centers at the C2 and C3 positions, with a substrate scope encompassing a range of indoles and structurally diverse organotin reagents. Gram-scale synthesis of 3aa further demonstrates the synthetic utility of this complementary Heck/Stille cascade.

## Conflicts of interest

There are no conflicts to declare.

## Supplementary Material

RA-016-D6RA02185A-s001

## Data Availability

The data supporting this article have been included as part of the supplementary information (SI). Supplementary information: experimental procedures spectroscopic data. See DOI: https://doi.org/10.1039/d6ra02185a.
