# Supplementary material for: Palladium-catalyzed intramolecular dearomatization of indoles via Heck/Stille cross-coupling with organostannanes
Source: RSC Adv. 2026 Apr 30;16(25):22465–9. doi: 10.1039/d6ra02185a (PMC13130186; doi:10.1039/d6ra02185a)

Supplementary Information (SI) for RSC Advances.
This journal is © The Royal Society of Chemistry 2026

# Supporting Information

## Palladium-catalyzed intramolecular dearomatization of indoles via Heck/Stille cross-coupling with organostannanes

Zhenjie Qi [a,1,*], Jiajing Hong [b,1], Baojing Ji [a], Chen Chen [a], Yixuan Xiao [a], Boxuan Song [a], Yanguo Lv [a], Zhenyu An [b,*], Peipei Ma [b,*]

[a] School of Resource & Environment and Safety Engineering, Jining University, Qufu 273155, Shandong, China.

[b] School of Pharmacy, Ningxia Medical University, Yinchuan 750000, Ningxia, China.

* Corresponding author.

E-mail address: 202310001@jnxy.edu.cn (Z. Qi), 20170024@nxmu.edu.cn (J. Hong), anzy@nxmu.edu.cn (Z. An)

[1] These authors contributed equally to this work.

General remark ........ S2

Experimental Section ........ S2

1 General procedure for the synthesis of benzoylacetonitrile. ........ S2

2 The process of optimizing reaction conditions ........ S4

3 Gram-scale synthesis ........ S5

4 The X-ray data of 3aa (CCDC 2521205) ........ S6

Reference ........ S7

Characterization of data for the palladium-catalyzed dearomatization products ........ S8

NMR spectra for the palladium-catalyzed dearomatization products ........ S19

## General remark

$^1$H NMR, $^{13}$C NMR, and $^{19}$F NMR spectra were recorded on Bruker 400M and 600M in $CDCl_3$. All $^1$H NMR, $^{13}$C NMR and $^{19}$F NMR chemical shifts were given as $\delta$ value (ppm) with reference to tetramethylsilane (TMS) as an internal standard. All compounds were further characterized by HRMS; copies of their $^1$H NMR, $^{13}$C NMR and $^{19}$F NMR spectra were provided. Products were purified by flash chromatography on 200-300 mesh silica gels. All melting points were determined without correction. All reagents were purchased commercially and used as received, unless otherwise noted.

## Experimental Section

## 1. General procedure for the synthesis of benzoylacetonitrile.

### 1.1 Synthesis of N-(2-bromobenzoyl) indole derivative 1a and 1q-1u.

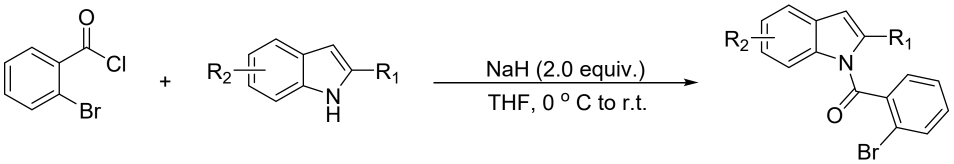


A 60% dispersion of NaH in mineral oil (2.0 equiv) was added to a stirred solution of the appropriate indole derivative (1 equiv, ~0.5 M) in THF at 0 °C and the corresponding solution was stirred for 5 minutes before warming to room temperature where it was stirred for 30 minutes. The solution of the sodium indolate was re-cooled to 0 °C at which time a solution of appropriate 2- bromobenzoyl chloride derivative (1.5 equiv, ~1 M) in THF was added dropwise. Once the addition was complete, the reaction was allowed to warm to room temperature and then was stirred at 65 °C for 30 minutes. At this time the extent of completion of the reaction was determined by conversion of the S4 indole derivate by TLC analysis. The reaction was cooled to room temperature and quenched with a saturated solution of $NH_4Cl$. The reaction mixture was then diluted with water and EtOAc, and after separating the layers, the aqueous layer was extracted with EtOAc (3x). The combined organic layers were washed sequentially with water and brine, dried over sodium sulfate, filtered and concentrated under reduced pressure. The crude N-(2-bromobenzoyl) indole derivative was purified by flash column gel chromatography using the indicated solvent system.

### 1.2 Synthesis of 2-aminoethyl indole derivatives 1b-1p.

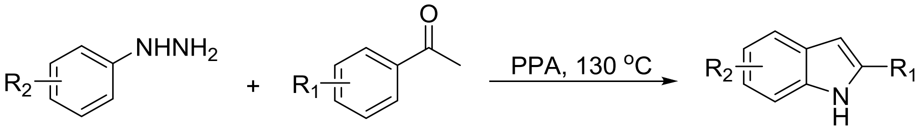


In air, a 100 mL round-bottom flask was charged with ketone (10 mmol, 1 equiv.), aromatic hydrazine (12 mmol, 1.2 equiv), and polyphosphoric acid (30 mL). The reaction mixture was heated in an oil bath at 130 °C for 1 h. Upon completion of the reaction, it was cooled to 60 °C. $H_2O$ (20 mL) was added and the reaction was stirred for another 30 minutes. Then the reaction was cooled to room temperature and extracted with ethyl acetate (3 * 30 mL). The combined organic layers were dried over anhydrous $Na_2SO_4$. After removal of the solvent, the crude reaction mixture was purified on silica gel eluting with petroleum ether/EtOAc to afford the substituted indole derivative.

**1.3 Synthesis of tributylphenyltin 2b-2g.**

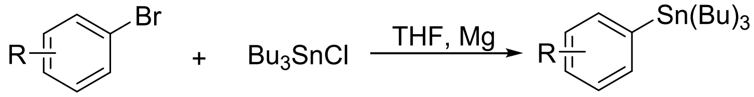


To a flame dried 50 mL two neck round-bottom flask equipped with a stir bar under an argon atmosphere, THF (6.0 mL) and magnesium turnings (391.8 mg, 16.0 mmol) were added. A solution of 1-bromo-benzene derivatives (1.2 mL, 9.6 mmol) in THF (5.0 mL) was added dropwise over 5 minutes. The mixture slowly turned dark brown and the stirred mixture was stirred for 1 h at ambient temperature. To this mixture was added dropwise $Bu_3SnCl$ (2.2 mL, 8.0 mmol) at ambient temperature. The mixture was heated to reflux overnight, and then it was cooled to ambient temperature. To the stirred mixture was added 14 mL of aqueous 1 M NaOH. After an hour, the mixture was transferred to a separatory funnel and the aqueous layer was extracted with $Et_2O$ (3 × 20 mL). The combined organic extracts were washed with brine (2 × 40 mL), dried over $MgSO_4$, and filtered. The solvent was removed by rotary evaporation, and the crude residue was purified by flash column chromatography on silica gel.

**1.4 Synthesis of 10 b-methy l -11 - pheny l - 10 b, 11 - dihydro - 6 H - isoindolo [2,1-a] indol - 6 - one derivatives 3aa.**

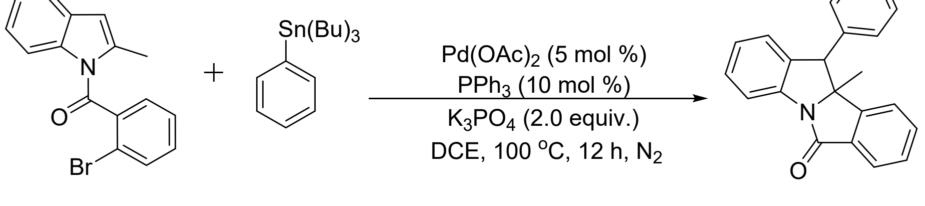


**1 a** **2a** **3aa**

A mixture of 10 b-methy l -11 - pheny l - 10 b, 11 - dihydro - 6 H - isoindolo [2,1-a]

indol - 6 - one **1a** (1 equiv., 0.2 mmol), tributylphenyltin **2a** (2 equiv., 0.4 mmol), $Pd(OAc)_2$ (0.05 equiv., 0.01 mmol), $PPh_3$ (0.1 equiv., 0.02 mmol), $K_3PO_4$ (2 equiv., 0.4 mmol), DCE (4 mL) were stirred at 100 °C under $N_2$ atmosphere for 12 h (TLC monitored). The solvent was evaporated in vacuo and the crude product was purified by column chromatography, eluting with petroleum ether/ethyl acetate (20 : 1) to afford the desired product **3aa**.

## 2. The process of optimizing reaction conditions

**Table S1. Screening of the solvent** [a]

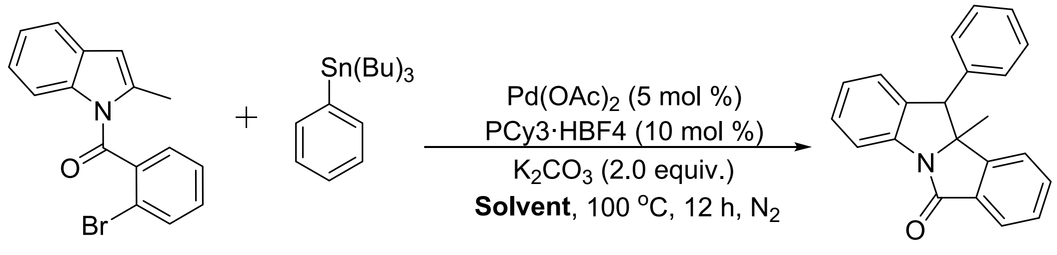


**1 a** **2a** **3aa**

| Entry | Solvent | Yield % |
|---|---|---|
| **1** | **DCE** | **55** |
| 2 | THF | 25 |
| 3 | DCM | 43 |
| 4 | Dioxane | 36 |
| 5 | Toluene | 30 |

[a] Reaction conditions: **1a** (0.2 mmol), **2a** (0.4 mmol), $Pd(OAc)_2$ (0.01 mmol), **L** (0.02 mmol), $K_2CO_3$ (0.4 mmol), and solvent (4 mL), 100 °C, under $N_2$ for 12 h. [b] Isolated yield.

**Table S2. Screening of the ligand** [a]

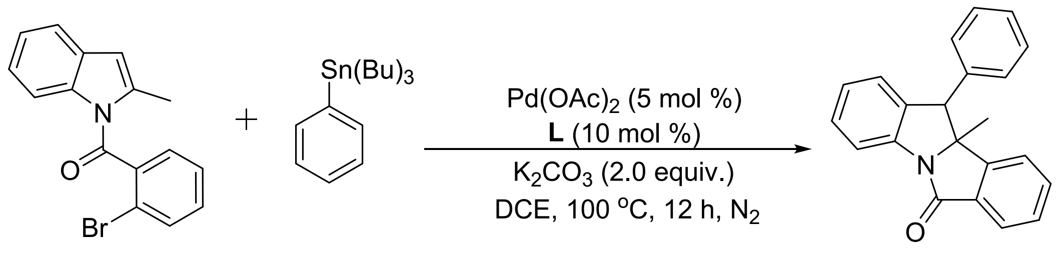


**1 a** **2a** **3aa**

| Entry | Ligand (10%) | Yield % |
|---|---|---|
| 1 | $PCy_3·HBF_4$ | 55 |
| 2 | $P^tBu_3·HBF_4$ | 45 |
| **3** | **$PPh_3$** | **66** |
| 4 | DPPB | 40 |
| 5 | XPhos | 28 |

[a] Reaction conditions: **1a** (0.2 mmol), **2a** (0.4 mmol), $Pd(OAc)_2$ (0.01 mmol), ligand (0.02 mmol), $K_2CO_3$ (0.4 mmol), and DCE (4 mL), 100 °C, under $N_2$ for 12 h. [b] Isolated yield.

**Table S3 Screening of the base** [a]

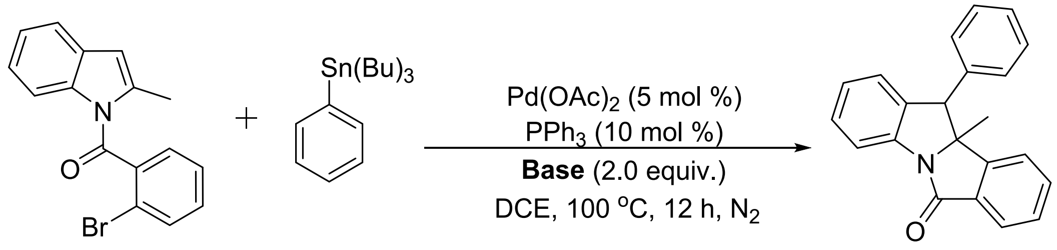


**1 a** **2a** **3aa**

| Entry | Base (2 equiv.) | Yield % |
|---|---|---|
| 1 | $K_2CO_3$ | 66 |
| 2 | $Na_2CO_3$ | 60 |
| 2 | $NaHCO_3$ | 59 |
| **3** | **$K_3PO_4$** | **87** |
| 4 | NaOAc | 45 |
| 5 | $Na_2HPO_4$ | 68 |

[a] Reaction conditions: **1a** (0.2 mmol), **2a** (0.4 mmol), $Pd(OAc)_2$ (0.01 mmol), $PPh_3$ (0.02 mmol), Base (0.4 mmol), and DCE (4 mL), 100 °C, under $N_2$ for 12 h. [b] Isolated yield.

**Table S4. Screening of the catalyst** [a]

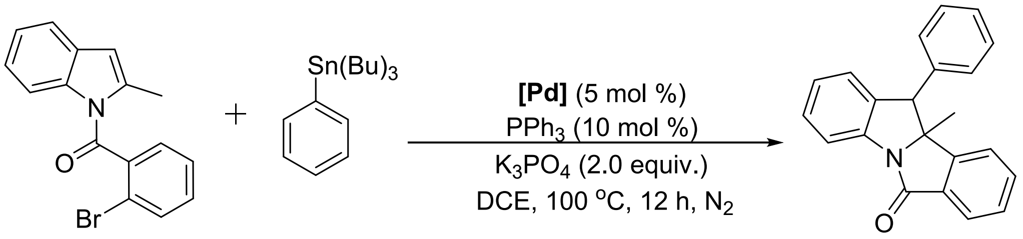


**1 a** **2a** **3aa**

| Entry | Catalyst (5 mol %) | Yield % |
|---|---|---|
| **1** | **$Pd(OAc)_2$** | **87** |
| 2 | $PdCl_2(CH_3CN)_2$ | 60 |
| 3 | $Pd(dba)_2$ | 59 |
| 4 | $Pd(PPh_3)_4$ | 56 |

[a] Reaction conditions: **1a** (0.2 mmol), **2a** (0.4 mmol), [Pd] (0.01 mmol), $PPh_3$ (0.02 mmol), $K_3PO_4$ (0.4 mmol), and DCE (4 mL), 100 °C, under $N_2$ for 12 h. [b] Isolated yield.

# 3. Gram-scale synthesis

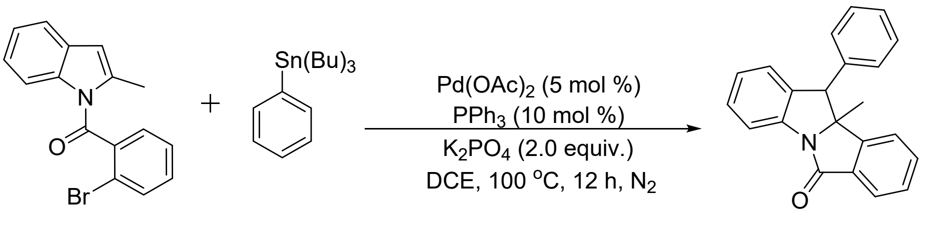


**1 a** **2a** **3aa**
**5 mmol** **10 mmol** **74% 1.15 g**

To demonstrate the synthetic application potentiality, gram-scale synthesis of product **3aa** was performed, and a decent yield of 74% was isolated smoothly.

## 4. The X-ray data of 3aa (CCDC 2521205)

An amount of 20 mg **3aa** were dissolved in dichloromethane on the brown small reagent bottle (5 mL), which acted as good solvent, and a layer of petroleum ether was injected on the dichloromethane of N,N-Dimethylformamide, and the cap is covered with a thin film, white crystals will be presented after seven days.

The crystal was kept at 273.15 K during data collection. Using Olex28[7], the structure was solved with the XT[8] structure solution program using Intrinsic Phasing and refined with the XL[9] refinement package using Least Squares minimisation. Nonhydrogen atoms were refined with anisotropic displacement parameters during the final cycles. All hydrogen atoms were placed by geometrical considerations and were added to the structure factor calculations.

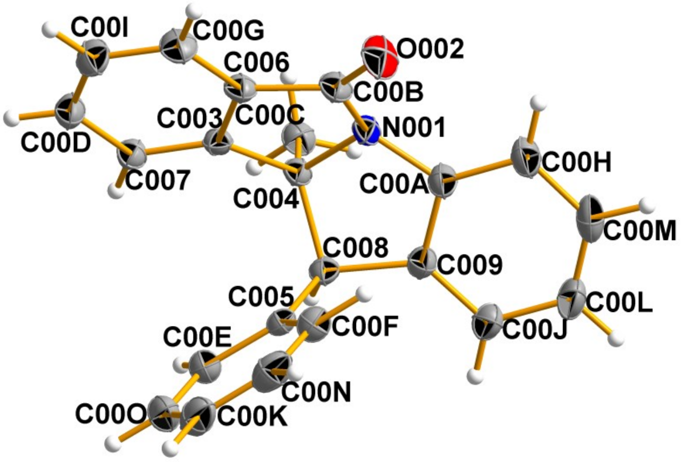


**Fig. S1.** X-ray crystal structure of compound **3aa**.

## Reference

1. Y. Horino, M. Sugata, I. Mutsuura, K. Tomohara, H. Abe, Org. lett. 19 (2017) 5968-5971.

2. K. J. Makaravage, A. F. Brooks, A. V. Mossine, M. S. Sanford, P. J. Scott, Org. lett. 18 (2016) 5440-5443.

3. J. E. Russell, E. D. Entz, I. M. Joyce, S. R. Neufeldt, ACS catal. 9 (2019) 3304-3310.

4. Q.-H. Liu, Y. Ma, H.-Y. Zhang, Y. Zhang, J. Zhao, X. Cao, Y.-P. Han, Y.-M. Liang, Org. Chem. Front. 11 (2024) 1357-1365.

5. P. S. Gribanov, Y. D. Golenko, M. A. Topchiy, L. I. Minaeva, A. F. Asachenko, M. S. Nechaev, Eur. J. Org. Chem. 2018 (2018) 120-125.

## Characterization of data for the palladium-catalyzed dearomatization products

**10B-methyl-11-phenyl-10b,11-dihydro-6*H*-isoindolo[2,1-*a*]indol-6-one (3aa)**

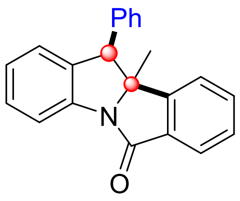


The product was purified by column chromatography (petroleum ether/EtOAc = 20:1). White solid (54.1 mg, 87% yield), melting point: 185-187 °C. **$^1$H NMR** (400 MHz, $CDCl_3$, ppm): δ 7.83 (d, *J* = 8.0 Hz, 1 H), 7.70 (d, *J* = 6.8 Hz, 1 H), 7.42-7.39 (m, 1 H), 7.29-7.05 (m, 5 H), 6.95-6.90 (m, 3 H), 6.55 (d, *J* = 6.0 Hz, 2 H), 4.36 (s, 1 H), 1.76 (s, 3 H); **$^{13}$C NMR** (100 MHz, $CDCl_3$, ppm): δ 168.6, 148.8, 140.8, 139.4, 139.1, 133.0, 132.1, 128.8, 128.4, 128.2, 127.8, 126.9, 126.7, 125.2, 124.4, 123.2, 117.3, 76.1, 57.3, 28.6. **HRMS** (ESI) calcd for $C_{22}H_{18}NO$ $[M+H]^+$ 312.1383, found: 312.1382.

**10B-cyclopropyl-11-phenyl-10b,11-dihydro-6*H*-isoindolo[2,1-*a*]indol-6-one (3ba)**

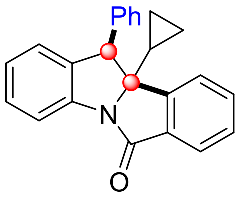


The product was purified by column chromatography (petroleum ether/EtOAc = 20:1). White solid (40.4 mg, 60% yield), melting point: 158-159 °C. **$^1$H NMR** (600 MHz, $CDCl_3$, ppm): δ 7.69 (d, *J* =6.6 Hz, 1 H), 7.61-7.60 (m, 1 H), 7.30-7.27 (m, 1 H), 7.18-7.13 (m, 2 H), 7.07-7.05 (m, 1 H), 7.03-7.00 (m, 1 H), 6.94-6.91 (m, 1 H), 6.89-6.83 (m, 3 H), 6.51 (d, *J* =6.6 Hz, 2 H), 4.54 (s, 1 H), 1.51-1.46 (m, 1 H), 0.43-0.39 (m, 1 H), 0.27-0.18 (m, 2 H), 0.12-0.07 (m, 1 H); **$^{13}$C NMR** (150 MHz, $CDCl_3$, ppm): δ 169.7, 148.4, 141.2, 140.7, 139.3, 132.9, 132.0, 128.6, 128.3, 128.1, 128.0, 126.9, 126.3, 125.1, 124.2, 123.4, 116.3, 78.1, 56.6, 22.0, 2.9, 0.9. **HRMS** (ESI) calcd for $C_{24}H_{20}NO$ $[M+H]^+$ 338.1540, found: 338.1540.

**10B,11-diphenyl-10b,11-dihydro-6*H*-isoindolo[2,1-*a*]indol-6-one (3ca)**

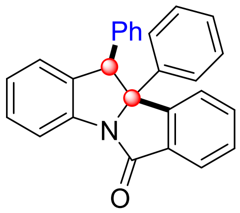


The product was purified by column chromatography (petroleum ether/EtOAc = 20:1). White solid (54.6 mg, 73% yield), melting point: 163-165 °C. **$^1$H NMR** (400 MHz, $CDCl_3$, ppm): δ 7.95 (d, *J* = 8 Hz, 1 H), 7.74 (d, *J* = 8 Hz, 2 H), 7.67-7.65 (m, 1 H), 7.43-7.32 (m, 3 H), 7.27-7.25 (m, 1 H), 7.20-7.15 (m, 3 H), 7.09 (d, *J* = 4.4 Hz, 2 H), 7.04-6.95 (m, 3 H), 6.74 (d, *J* = 6.8 Hz, 2 H), 5.00 (s, 1 H); **$^{13}$C NMR** (100 MHz, $CDCl_3$, ppm): δ 169.3, 148.1, 144.0, 140.5, 140.0, 138.7, 132.4, 132.3, 129.0, 128.9, 128.6, 128.2, 128.0, 127.2, 126.4, 125.5, 125.2, 124.4, 124.3, 117.1, 81.3, 59.2. **HRMS** (ESI) calcd for $C_{27}H_{20}NO$ $[M+H]^+$ 374.1540, found: 374.1539.

**11-Phenyl-10b-(o-tolyl)-10b,11-dihydro-6*H*-isoindolo[2,1-*a*]indol-6-one (3da)**

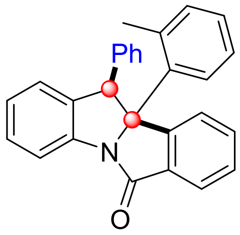

The product was purified by column chromatography (petroleum ether/EtOAc = 20:1). White solid (58.1 mg, 75% yield), melting point: 204-206 °C. **$^1$H NMR** (600 MHz, $CDCl_3$, ppm): δ 7.85-7.85 (m, 2 H), 7.63-7.61 (m, 1 H), 7.33-7.30 (m, 1 H), 7.17-7.11 (m, 4 H), 7.08-6.99 (m, 4 H), 6.95-6.89 (m, 3 H), 6.69 (d, $J$ =6.6 Hz, 2 H), 5.12 (s, 1 H), 2.58 (m, 3 H); **$^{13}$C NMR** (150 MHz, $CDCl_3$, ppm): δ 168.7, 147.7, 140.7, 140.1, 139.5, 139.4, 135.2, 133.5, 132.8, 132.4, 128.8, 128.6, 128.3, 128.1, 127.8, 127.2, 126.7, 126.6, 126.1, 125.5, 124.2, 124.2, 117.0, 83.2, 58.3, 23.0. **HRMS** (ESI) calcd for $C_{28}H_{22}NO$ $[M+H]^+$ 388.1696, found: 388.1696.

**11-Phenyl-10b-(m-tolyl)-10b,11-dihydro-6*H*-isoindolo[2,1-*a*]indol-6-one (3ea)**

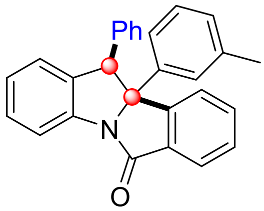

The product was purified by column chromatography (petroleum ether/EtOAc = 20:1). White solid (61.4 mg, 79% yield), melting point: 145-147 °C. **$^1$H NMR** (600 MHz, $CDCl_3$, ppm): δ 7.95 (d, $J$ =7.8 Hz, 1 H), 7.66 (d, $J$ =6.6 Hz, 1 H), 7.57 (d, $J$ =7.8 Hz, 1 H), 7.50 (s, 1 H), 7.40-7.37 (m, 1 H), 7.24-7.22 (m, 1 H), 7.19-7.12 (m, 3 H), 7.08-7.03 (m, 3 H), 7.01-6.99 (m, 2 H), 6.96-6.93 (m, 1 H), 6.74 (d, $J$ =6.6 Hz, 2 H), 5.02 (s, 1 H), 2.31 (s, 3 H); **$^{13}$C NMR** (150 MHz, $CDCl_3$, ppm): δ 169.3, 148.2, 143.8, 140.5, 140.0, 138.7, 138.7, 132.3, 132.2, 128.9, 128.8, 128.8, 128.5, 128.2, 128.1, 127.1, 126.4, 125.6, 125.5, 124.3, 124.2, 122.3, 117.0, 81.3, 59.0, 21.8. **HRMS** (ESI) calcd for $C_{28}H_{22}NO$ $[M+H]^+$ 388.1696, found: 388.1694.

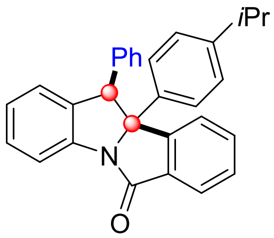

**10B-(4-isopropylphenyl)-11-phenyl-10b,11-dihydro-6*H*-isoindolo[2,1-*a*]indol-6-one (3fa)**

The product was purified by column chromatography (petroleum ether/EtOAc = 20:1). White solid (52.4 mg, 63% yield), melting point: 138-140 °C. **$^1$H NMR** (600 MHz, $CDCl_3$, ppm): δ 7.96 (d, $J$ =7.8 Hz, 1 H), 7.65-7.63 (m, 3 H), 7.41-7.37 (m, 1 H), 7.19-7.14 (m, 5 H), 7.09-7.06 (m, 2 H), 7.02-6.99 (m, 2 H), 6.96-6.94 (m, 1 H), 6.73 (d, $J$ =6.6 Hz, 2 H), 4.99 (s, 1 H), 2.86-2.81 (m, 1 H), 1.18 (d, $J$ =6.6 Hz, 6 H); **$^{13}$C NMR** (150 MHz, $CDCl_3$, ppm): δ 169.3, 148.6, 148.3, 141.2, 140.6, 140.1, 138.8, 132.4, 132.2, 128.8, 128.5, 128.2, 128.1, 127.1, 127.04, 126.4, 125.5, 125.1, 124.3, 124.3, 117.1, 81.2, 59.2, 33.7, 24.0. **HRMS** (ESI) calcd for $C_{30}H_{26}NO$ $[M+H]^+$ 416.2009, found: 416.2009.

**10B-(3-methoxyphenyl)-11-phenyl-10b,11-dihydro-6*H*-isoindolo[2,1-*a*]indol-6-one (3ga)**

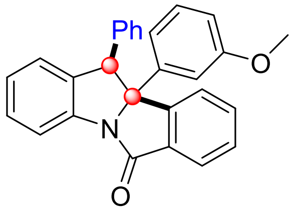

The product was purified by column chromatography (petroleum ether/EtOAc = 20:1). White solid (40.3 mg, 50% yield), melting point: 171-172 °C. **$^{1}$H NMR** (600 MHz, $CDCl_3$, ppm): δ 7.94 (d, *J* =7.8 Hz, 1 H), 7.65-7.64 (m, 1 H), 7.40-7.35 (m, 2 H), 7.29-7.25 (m, 2 H), 7.20-7.16 (m, 3 H), 7.09-7.07 (m, 2 H), 7.02-6.99 (m, 2 H), 6.97-6.94 (m, 1 H), 6.78-6.72 (m, 3 H), 5.01 (s, 1 H), 3.75 (s, 3 H); **$^{13}$C NMR** (150 MHz, $CDCl_3$, ppm): δ 169.3, 160.0, 147.9, 145.6, 140.4, 140.0, 139.0, 132.3, 132.3, 130.1, 128.9, 128.6, 128.2, 128.2, 127.2, 126.4, 125.5, 124.3, 124.3, 117.6, 117.1, 112.5, 111.8, 81.3, 59.1, 55.4. **HRMS** (ESI) calcd for $C_{28}H_{22}NO_2$ $[M+H]^+$ 404.1646, found: 404.1646.

**10B-([1,1'-biphenyl]-4-yl)-11-phenyl-10b,11-dihydro-6H-isoindolo[2,1-a]indol-6-one (3ha)**

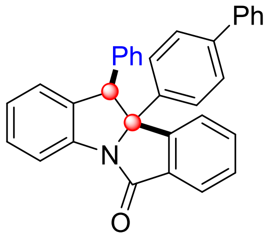

The product was purified by column chromatography (petroleum ether/EtOAc = 20:1). White solid (64.7 mg, 72% yield), melting point: 182-183 °C. **$^{1}$H NMR** (600 MHz, $CDCl_3$, ppm): δ 7.90 (d, *J* =7.8 Hz, 1 H), 7.73 (d, *J* =8.4 Hz, 2 H), 7.61-7.60 (m, 1 H), 7.49 (d, *J* =8.4 Hz, 2 H), 7.44-7.43 (m, 2 H), 7.37-7.32 (m, 3 H), 7.27-7.24 (m, 1 H), 7.16-7.11 (m, 3 H), 7.05-7.02 (m, 2 H), 6.97-6.95 (m, 2 H), 6.92-6.90 (m, 1 H), 7.69 (d, *J* =7.2 Hz, 2 H), 4.97 (s, 1 H); **$^{13}$C NMR** (150 MHz, $CDCl_3$, ppm): δ 169.3, 148.0, 142.9, 141.1, 140.5, 140.5, 140.0, 138.7, 132.4, 132.4, 129.0, 128.9, 128.6, 128.3, 128.2, 127.8, 127.6, 127.2, 127.2, 126.5, 125.6, 125.6, 124.5, 124.2, 117.1, 81.2, 59.2. **HRMS** (ESI) calcd for $C_{33}H_{24}NO$ $[M+H]^+$ 450.1852, found: 450.1853.

**10B-(4-fluorophenyl)-11-phenyl-10b,11-dihydro-6*H*-isoindolo[2,1-*a*]indol-6-one (3ia)**

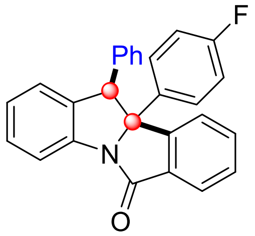

The product was purified by column chromatography (petroleum ether/EtOAc = 20:1). White solid (58.7 mg, 75% yield), melting point: 222-224 °C. **$^{1}$H NMR** (400 MHz, $CDCl_3$, ppm): δ 7.94 (d, *J* = 7.6 Hz, 1 H), 7.73-7.65 (m, 3 H), 7.44-7.38 (m, 1 H), 7.21-7.14 (m, 3 H), 7.10-7.09 (m, 2 H), 7.04-6.93 (m, 5 H), 6.74-6.71 (m, 2 H), 4.96 (s, 1 H); **$^{13}$C NMR** (100 MHz, $CDCl_3$, ppm): δ 169.2, 162.3 (d, *J* = 245.5 Hz, 1 C), 147.9, 140.2, 139.8, 139.6 (d, *J* = 3.2 Hz, 1 C), 138.4, 132.4, 132.2, 129.0,

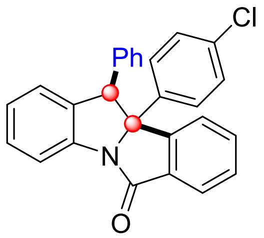


128.6, 128.3, 128.1, 127.3, 126.9, 126.9, 126.4, 125.7, 124.3 (d, $J$ = 34.3 Hz, 1 C), 116.5 (d, $J$ = 112.5 Hz, 1 C), 115.8, 80.8, 59.2; **$^{19}$F NMR** (377 MHz, $CDCl_3$, ppm) δ -114.4. **HRMS** (ESI) calcd for $C_{27}H_{19}FNO$ $[M+H]^+$ 392.1446, found: 392.1446.

**10B-(4-chlorophenyl)-11-phenyl-10b,11-dihydro-6*H*-isoindolo[2,1-*a*]indol-6-one (3ja)**

The product was purified by column chromatography (petroleum ether/EtOAc = 20:1). White solid (50.5 mg, 62% yield), melting point: 212-214 °C. **$^1$H NMR** (400 MHz, $CDCl_3$, ppm): δ 7.94 (d, $J$ =7.6 Hz, 1 H), 7.69-7.65 (m, 3 H), 7.44-7.39 (m, 1 H), 7.32-7.29 (m, 2 H), 7.21-7.14 (m, 3 H), 7.11-7.08 (m, 2 H), 7.04-6.94 (m, 3 H), 6.73-6.71 (m, 2 H), 4.94 (s, 1 H); **$^{13}$C NMR** (100 MHz, $CDCl_3$, ppm): δ 169.1, 147.6, 142.4, 140.1, 139.8, 138.3, 133.9, 132.4, 132.3, 129.2, 129.1, 128.6, 128.4, 128.1, 127.3, 126.6, 126.4, 125.7, 124.5, 124.1, 117.1, 80.9, 59.1. **HRMS** (ESI) calcd for $C_{27}H_{19}ClNO$ $[M+H]^+$ 408.1150, found: 408.1150.

**10B-([1,1'-biphenyl]-3-yl)-11-phenyl-10b,11-dihydro-6H-isoindolo[2,1-a]indol-6-one (3ka)**

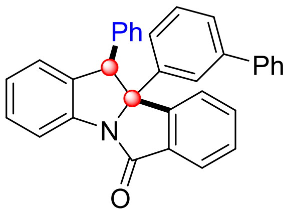


The product was purified by column chromatography (petroleum ether/EtOAc = 20:1). White solid (74.9 mg, 83% yield), melting point: 205-206 °C. **$^1$H NMR** (600 MHz, $CDCl_3$, ppm): δ 7.97 (d, $J$ =7.8 Hz, 1 H), 7.92 (s, 1 H), 7.75 (d, $J$ =7.8 Hz, 1 H), 7.67 (d, $J$ =7.2 Hz, 1 H), 7.53 (d, $J$ =7.2 Hz, 2 H), 7.47-7.40 (m, 5 H), 7.37-7.35 (m, 1 H), 7.25-7.23 (m, 1 H), 7.22-7.17 (m, 2 H), 7.11-7.08 (m, 2 H), 7.03-7.01 (m, 2 H), 6.98-6.96 (m, 1 H), 6.76 (d, $J$ =7.2 Hz, 2 H), 5.07 (s, 1 H); **$^{13}$C NMR** (150 MHz, $CDCl_3$, ppm): δ 169.3, 148.0, 144.5, 142.1, 141.0, 140.5, 140.0, 138.6, 132.4, 132.4, 129.5, 129.0, 128.6, 128.3, 128.2, 127.7, 127.4, 127.2, 127.0, 126.4, 125.6, 124.4, 124.2, 124.1, 123.9, 117.1, 81.4, 59.3, 29.8. **HRMS** (ESI) calcd for $C_{33}H_{24}NO$ $[M+H]^+$ 450.1852, found: 450.1854.

**10B-([1,1'-biphenyl]-4-yl)-11-phenyl-10b,11-dihydro-6H-isoindolo[2,1-a]indol-6-one (3la)**

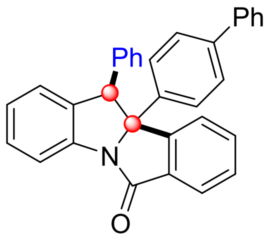


The product was purified by column chromatography (petroleum ether/EtOAc = 20:1). White solid (53.9 mg, 54% yield), melting point: 182-183 °C. **$^1$H NMR** (400 MHz, $CDCl_3$, ppm): δ 7.97 (d, $J$ =7.6 Hz, 1 H), 7.81 (d, $J$ =8.4 Hz, 2 H), 7.69-7.67 (m, 1 H), 7.57-7.50 (m, 4 H), 7.45-7.39 (m, 3 H), 7.34-7.31

(m, 1 H), 7.24-7.18 (m, 3 H), 7.13-7.11 (m, 2 H), 7.05-6.98 (m, 3 H), 6.76 (d, *J* =7.2 Hz, 2 H), 5.05 (s, 1 H); **$^{13}$C NMR** (100 MHz, $CDCl_3$, ppm): δ 169.3, 148.0, 142.9, 141.0, 140. 5, 140.0, 138.6, 132.4, 129.0, 128.9, 128.6, 128.3, 128.2, 127.8, 127.6, 127.2, 127.2, 126.5, 125.6, 125.6, 124.4, 124.2, 117.1, 81.2, 59.1,. **HRMS** (ESI) calcd for $C_{33}H_{24}NO$ $[M+H]^+$ 450.1852, found: 450.1853.

**11-Phenyl-10b-(4-(trifluoromethyl)phenyl)-10b,11-dihydro-6*H*-isoindolo[2,1-*a*]indol-6-one (3ma)**

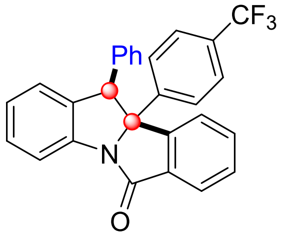


The product was purified by column chromatography (petroleum ether/EtOAc = 20:1). White solid (60.0 mg, 68% yield), melting point: 173-175 °C. **$^1$H NMR** (600 MHz, $CDCl_3$, ppm): δ 7.89 (d, *J* =8.4 Hz, 1 H), 7.80 (d, *J* =7.8 Hz, 2 H), 7.62-7.59 (m, 1 H), 7.53 (d, *J* =8.4 Hz, 2 H), 7.37-7.34 (m, 1 H), 7.15-7.10 (m, 3 H), 7.05-7.01 (m, 2 H), 6.97-6.90 (m, 3 H), 6.66 (d, *J* =7.2 Hz, 2 H), 4.89 (s, 1 H); **$^{13}$C NMR** (150 MHz, $CDCl_3$, ppm): δ 169.0, 147.9, 147.0, 139.8 (q, *J* =33.3 Hz, 1 C), 138.0, 132.4, 132.2, 130.2 (q, *J* =32.5 Hz, 1 C), 128.2 (q, *J* =260.7 Hz, 1 C), 128.6, 128.5, 128.0, 126.3, 126.0 (q, *J* =3.9 Hz, 1 C), 125.7, 125.5, 123.9 (q, *J* =270.3 Hz, 1 C), 124.5, 124.0, 117.0, 80.9, 59.1; **$^{19}$F NMR** (565 MHz, $CDCl_3$, ppm) δ -62.6. **HRMS** (ESI) calcd for $C_{28}H_{19}F_3NO$ $[M+H]^+$ 442.1414, found: 442.1414.

**10B-(naphthalen-2-yl)-11-phenyl-10b,11-dihydro-6*H*-isoindolo[2,1-*a*]indol-6-one (3na)**

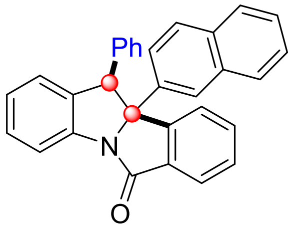


The product was purified by column chromatography (petroleum ether/EtOAc = 20:1). White solid (64.3 mg, 76% yield), melting point: 238-240 °C. **$^1$H NMR** (400 MHz, $CDCl_3$, ppm): δ 8.11 (s, 1 H), 8.03 (d, *J* = 7.6 Hz, 1 H), 7.94-7.86 (m, 2 H), 7.79-7.74 (m, 2 H), 7.69-7.67 (m, 1 H), 7.45-7.39 (m, 3 H), 7.27-7.24 (m, 1 H), 7.19-7.12 (m, 2 H), 7.09-6.95 (m, 5 H), 6.81 (d, *J* = 6.8 Hz, 2 H), 5.14 (s, 1 H); **$^{13}$C NMR** (100 MHz, $CDCl_3$, ppm): δ 169.3, 148.0, 140.8, 140.5, 140.0, 138.6, 133.2, 132.9, 132.3, 132.3, 129.1, 128.9, 128.6, 128.3, 128.2, 128.2, 127.6, 127.2, 126.7, 126.5, 126.4, 125.6, 124.4, 124.2, 123.5, 123.5, 117.1, 81.4, 58.8. **HRMS** (ESI) calcd for $C_{31}H_{22}NO$ $[M+H]^+$ 424.1696, found: 424.1696.

**11-Phenyl-10b-(thiophen-2-yl)-10b,11-dihydro-6*H*-isoindolo[2,1-*a*]indol-6-one (3oa)**

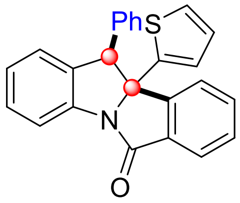


The product was purified by column chromatography (petroleum ether/EtOAc = 20:1). White solid (51.5 mg, 68% yield), melting point: 230-233 °C. **$^{1}$H NMR** (400 MHz, $CDCl_3$, ppm): δ 7.88 (d, $J$ =8.0 Hz, 1 H), 7.68-7.66 (m, 1 H), 7.44-7.39 (m, 1 H), 7.27-7.11 (m, 7 H), 7.00-6.88 (m, 4 H), 6.67 (d, $J$ =6.4 Hz, 2 H), 5.05 (s, 1 H); **$^{13}$C NMR** (100 MHz, $CDCl_3$, ppm): δ 168.9, 148.1, 147.3, 140.0, 139.8, 138.6, 132.3, 132.2, 129.1, 128.6, 128.5, 128.2, 127.3, 127.0, 126.5, 125.7, 124.8, 124.6, 124.4, 124.2, 117.4, 79.2, 59.5. **HRMS** (ESI) calcd for $C_{25}H_{18}NOS$ $[M+H]^+$ 380.1104, found: 380.1104.

**11-Phenyl-10b,11-dihydro-6*H*-isoindolo[2,1-*a*]indol-6-one (3pa)**

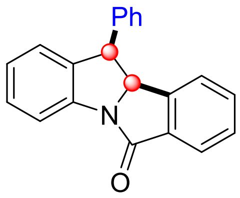


The product was purified by column chromatography (petroleum ether/EtOAc = 20:1). White solid (21.4 mg, 36% yield), melting point: 188-190 °C. **$^{1}$H NMR** (400 MHz, $CDCl_3$, ppm): δ 7.84 (d, $J$ =7.6 Hz, 1 H), 7.75-7.73 (m, 1 H), 7.42-7.38 (m, 1 H), 7.33-7.28 (m, 2 H), 7.20-7.10 (m, 3 H), 7.01-6.93 (m, 3 H), 6.63-6.60 (m, 2 H), 5.79 (d, $J$ =8.4 Hz, 1 H), 4.77 (d, $J$ =8.8 Hz, 1 H); **$^{13}$C NMR** (100 MHz, $CDCl_3$, ppm): δ 168.7, 143.3, 140.4, 139.9, 139.5, 134.6, 132.1, 128.9, 128.4, 128.4, 128.0, 127.1, 126.5, 125.1, 124.4, 124.0, 116.5, 69.6, 49.9. **HRMS** (ESI) calcd for $C_{21}H_{16}NO$ $[M+H]^+$ 298.1227, found: 298.1227.

**2,10B-dimethyl-11-phenyl-10b,11-dihydro-6*H*-isoindolo[2,1-*a*]indol-6-one (3qa)**

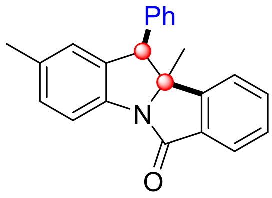


The product was purified by column chromatography (petroleum ether/EtOAc = 20:1). White solid (40.3 mg, 62% yield), melting point: 205-206 °C. **$^{1}$H NMR** (400 MHz, $CDCl_3$, ppm): δ 7.72-7.67 (m, 2 H), 7.28-7.20 (m, 3 H), 7.04 (d, $J$ = 6.4 Hz, 1 H), 6.99 (s, 1 H), 6.96-6.88 (s, 3 H), 6.56 (d, $J$ = 4.0 Hz, 2 H), 4.31 (s, 1 H), 2.30 (s, 3 H), 1.75 (s, 3 H); **$^{13}$C NMR** (100 MHz, $CDCl_3$, ppm): δ 168.5, 148.8, 140.9, 139.2, 137.1, 134. 9, 133.2, 132.0, 129.4, 128.4, 128.1, 127.8, 127.3, 126.9, 124.3, 123.1, 116.9, 76.3, 57.2, 28.5, 21.3. **HRMS** (ESI) calcd for $C_{23}H_{20}NO$ $[M+H]^+$ 326.1540, found: 326.1540.

**2-Methoxy-10b-methyl-11-phenyl-10b,11-dihydro-6*H*-isoindolo[2,1-*a*]indol-6-one (3ra)**

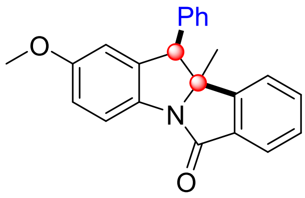


The product was purified by column chromatography (petroleum ether/EtOAc = 20:1). White solid (66.2 mg, 97% yield), melting point: 54-55 °C. **$^{1}$H NMR** (400 MHz, $CDCl_3$, ppm): δ 7.73 (d, $J$ = 8.4 Hz, 1 H), 7.67 (d, $J$ = 6.4 Hz, 1 H),

7.28-7.20 (m, 2 H), 7.05 (d, *J* = 6.4 Hz, 1 H), 6.96-6.88 (m, 4 H), 6.74 (d, *J* = 2.8 Hz, 1 H), 6.57 (d, *J* = 6.0 Hz, 2 H), 4.31 (s, 1 H), 3.74 (s, 3 H) 1.75 (s, 3 H); **$^{13}$C NMR** (100 MHz, $CDCl_3$, ppm): δ 168.5, 157.6, 148.7, 140.6, 140.5, 133.2, 133.0, 131.9, 128.4, 128.2, 127. 8, 126.9, 124.2, 123.1, 117.8, 114.0, 112.6, 76.5, 57.5, 55.7, 28.4. **HRMS** (ESI) calcd for $C_{23}H_{20}NO_2$ $[M+H]^+$ 342.1489, found: 342.1489.

**2-Fluoro-10b-methyl-11-phenyl-10b,11-dihydro-6*H*-isoindolo[2,1-*a*]indol-6-one (3sa)**

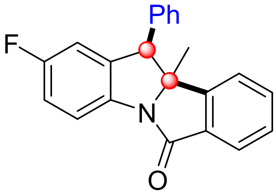


The product was purified by column chromatography (petroleum ether/EtOAc = 20:1). White solid (49.4 mg, 75% yield), melting point: 177-179 °C. **$^1$H NMR** (400 MHz, $CDCl_3$, ppm): δ 7.79-7.75 (m, 1 H), 7.69 (d, *J* = 6.8 Hz, 1 H), 7.31-7.23 (m, 2 H), 7.13-7.05 (m, 2 H), 6.98-6.89 (m, 4 H), 6.55 (d, *J* = 5.6 Hz, 2 H), 4.34 (s, 1 H), 1.77 (s, 3 H); **$^{13}$C NMR** (100 MHz, $CDCl_3$, ppm): δ 168.7, 160.4 (d, *J* = 241.9 Hz, 1 C), 148.6, 140.8 (d, *J* = 8.1 Hz, 1 C), 140.1, 135.5 (d, *J* = 2.2 Hz, 1 C), 132.7, 132.2, 128.5, 128.3, 127.7, 127.2, 124.4, 123.1, 118.0 (d, *J* = 8.6 Hz, 1 C), 115.5 (d, *J* = 23.4 Hz, 1 C), 114.0 (d, *J* = 23.9 Hz, 1 C), 76.6, 57.3 (d, *J* = 1.8 Hz, 1 C), 28.4; **$^{19}$F NMR** (377 MHz, $CDCl_3$, ppm) δ -117.4. **HRMS** (ESI) calcd for $C_{22}H_{17}FNO$ $[M+H]^+$ 330.1289, found: 330.1289.

**3-Fluoro-10b-methyl-11-phenyl-10b,11-dihydro-6*H*-isoindolo[2,1-*a*]indol-6-one (3ta)**

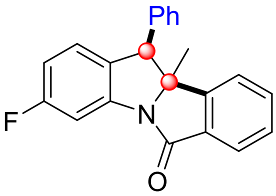


The product was purified by column chromatography (petroleum ether/EtOAc = 20:1). White solid (51.3 mg, 78% yield), melting point: 165-167 °C. **$^1$H NMR** (400 MHz, $CDCl_3$, ppm): δ 7.69 (d, *J* = 6.8 Hz, 1 H), 7.56-7.53 (m, 1 H), 7.31-7.22 (m, 2 H), 7.13-7.09 (m, 1 H), 7.05 (d, *J* = 6.8 Hz, 1 H), 6.97-6.92 (m, 3 H), 6.84-6.79 (m, 1 H), 6.56-6.53 (m, 2 H), 4.33 (s, 1 H), 1.77 (s, 3 H); **$^{13}$C NMR** (100 MHz, $CDCl_3$, ppm): δ 168.6, 163.2 (d, *J* = 244.1 Hz, 1 C), 148.8, 140.7, 140.6, 134.4 (d, *J* = 2.9 Hz, 1 C), 132.6, 132.4, 128.4, 128.3, 127.7, 127.5, 127.4, 127.1, 123.9 (d, *J* = 130.1 Hz, 1 C), 112.0, 111.8, 105.2 (d, *J* = 26.4 Hz, 1 C), 56.7, 28.7; **$^{19}$F NMR** (377 MHz, $CDCl_3$, ppm) δ -112.4. **HRMS** (ESI) calcd for $C_{22}H_{17}FNO$ $[M+H]^+$ 330.1289, found: 330.1289.

**2-Chloro-10b-methyl-11-phenyl-10b,11-dihydro-6*H*-isoindolo[2,1-*a*]indol-6-one (3ua)**

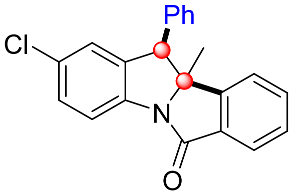


The product was purified by column chromatography (petroleum ether/EtOAc = 20:1). White solid (53.1 mg, 77%

yield), melting point: 145-148 °C. **$^1$H NMR** (400 MHz, $CDCl_3$, ppm): δ 7.75 (d, $J$ = 8.4 Hz, 1 H), 7.39 (d, $J$ = 8.0 Hz, 1 H), 7.39-7.36 (m, 1 H), 7.30-7.22 (m, 2 H), 7.16 (d, $J$ = 2.0 Hz, 1 H), 7.04 (d, $J$ = 9.2 Hz, 1 H), 6.97-6.92 (m, 3 H), 6.55-6.53 (m, 2 H), 4.33 (s, 1 H), 1.75 (s, 3 H); **$^{13}$C NMR** (100 MHz, $CDCl_3$, ppm): δ 168.6, 148.6, 140.8, 140.1, 138.1, 132.6, 132.4, 130.3, 129.0, 128.5, 128.4, 127.7, 127.2, 127.0, 124.5, 123.2, 118.1, 76.4, 57.2, 28.5. **HRMS** (ESI) calcd for $C_{22}H_{17}ClNO$ $[M+H]^+$ 346.0994, found: 346.0994.

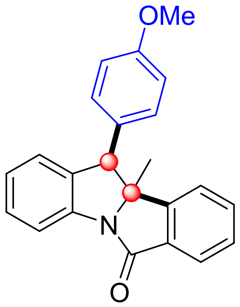


**11-(4-Methoxyphenyl)-10b-methyl-10b,11-dihydro-6*H*-isoindolo[2,1-*a*]indol-6-one (3ab)**

The product was purified by column chromatography (petroleum ether/EtOAc = 20:1). White solid (49.1 mg, 72% yield), melting point: 192-193 °C. **$^1$H NMR** (400 MHz, $CDCl_3$, ppm): δ 7.82 (d, $J$ =8.0 Hz, 1 H), 7.71-7.69 (m, 1 H), 7.42-7.38 (m, 1 H), 7.33-7.24 (m, 2 H), 7.18-7.07 (m, 3 H), 6.47 (s, 4 H), 4.32 (s, 1 H), 3.58 (s, 3 H), 1.74 (s, 3 H); **$^{13}$C NMR** (100 MHz, $CDCl_3$, ppm) : δ 168.7, 158.3, 149.0, 139.4, 139.2, 133.3, 133.0, 132.2, 128.8, 128.8, 128.2, 126.7, 125.2, 124.4, 123.2, 117.2, 113.7, 76.1, 56.5, 55.1, 28.5. **HRMS** (ESI) calcd for $C_{23}H_{22}NO_2$ $[M+H]^+$ 342.1489, found: 342.1489.

**11-(4-Fluorophenyl)-10b-methyl-10b,11-dihydro-6*H*-isoindolo[2,1-*a*]indol-6-one (3ac)**

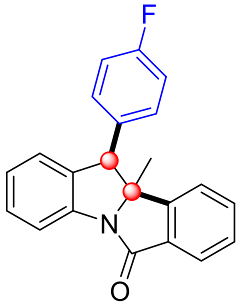


The product was purified by column chromatography (petroleum ether/EtOAc = 20:1). White solid (35.5 mg, 54% yield), melting point: 174-175 °C. **$^1$H NMR** (400 MHz, $CDCl_3$, ppm): δ 7.83 (d, $J$ =7.6 Hz, 1 H), 7.72-7.70 (m, 1 H), 7.44-7.40 (m, 1 H), 7.34-7.26 (m, 2 H), 7.18-7.12 (m, 2 H), 7.07 (d, $J$ =6.8 Hz, 1 H), 6.66-6.62 (m, 2 H), 6.53-6.48 (m, 2 H), 4.36-4.34 (m, 1 H), 1.76 (s, 3 H); **$^{13}$C NMR** (100 MHz, $CDCl_3$, ppm): δ 168.5, 161.6 (d, $J$ =244.4 Hz, 1 C), 148.6, 139.1 (d, $J$ =35.9 Hz, 1 C), 136.7 (d, $J$ =3.3 Hz, 1 C), 132.3, 129.3, 129.2, 129.1, 129.0, 128.6, 128.4, 126.7, 125.3, 124.5, 123.1, 117.4, 115.3 (d, $J$ =21.3 Hz, 1 C), 76.0, 56.4; **$^{19}$F NMR** (377 MHz, $CDCl_3$, ppm): δ -115.5. **HRMS** (ESI) calcd for $C_{22}H_{17}FNO$ $[M+H]^+$ 330.1289, found: 330.1289.

**11-([1,1'-Biphenyl]-4-yl)-10b-methyl-10b,11-dihydro-6*H*-isoindolo[2,1-*a*]indol-6-one (3ad)**

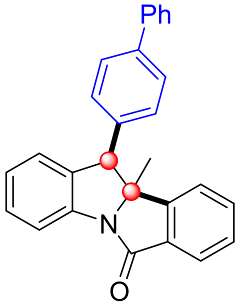


The product was purified by column chromatography (petroleum ether/EtOAc = 20:1). White solid (48.0 mg, 62% yield), melting

point: 197-198 °C. **$^1$H NMR** (400 MHz, $CDCl_3$, ppm): δ 7.85 (d, $J$ =8.0 Hz, 1 H), 7.70 (d, $J$ = 7.2 Hz, 1 H), 7.45-7.40 (m, 1 H), 7.38-7.35 (m, 2 H), 7.34-7.30 (m, 2 H), 7.28-7.14 (m, 7 H), 7.13-7.09 (m, 1 H), 6.62 (d, $J$ = 8.0 Hz,, 2 H), 4.41 (s, 1 H), 1.78 (s, 3 H); **$^{13}$C NMR** (100 MHz, $CDCl_3$, ppm): δ 168.6, 148.8, 140.5, 140.0, 139.6, 139.4, 139.0, 133.0, 132.2, 128.9, 128.8, 128.3, 128.2, 127.3, 127.0, 126.9, 126.7, 125.2, 124.4, 123.2, 117.3, 76.2, 56.9, 28.6. **HRMS** (ESI) calcd for $C_{28}H_{22}NO$ $[M+H]^+$ 388.1696, found: 388.1696.

**11-(3-Acetylphenyl)-10b-methyl-10b,11-dihydro-6*H*-isoindolo[2,1-*a*]indol-6-one (3ae)**

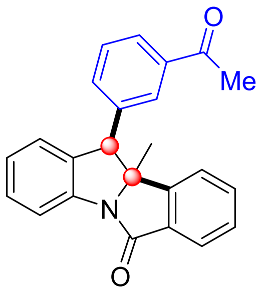


The product was purified by column chromatography (petroleum ether/EtOAc = 20:1). White oil (55.1 mg, 78% yield). **$^1$H NMR** (600 MHz, $CDCl_3$, ppm): δ 7.85 (d, $J$ =7.8 Hz, 1 H), 7.70 (d, $J$ =6.6 Hz, 1 H), 7.51 (d, $J$ =7.2 Hz, 1 H), 7.45-7.42 (m, 2 H), 7.29-7.23 (m, 2 H), 7.19-7.13 (m, 2 H), 7.06 (d, $J$ =7.2 Hz, 1 H), 6.99-6.96 (m, 1 H), 6.50 (d, $J$ =7.8 Hz, 1 H), 4.45 (s, 1 H), 2.41 (s, 3 H), 1.79 (s, 3 H); **$^{13}$C NMR** (150 MHz, $CDCl_3$, ppm): δ 198.1, 168.5, 148.5, 141.5, 139.3, 138.6, 136.8, 133.0, 132.4, 132.2, 129.2, 128.9, 128.5, 127.7, 127.0, 126.7, 125.4, 124.4, 123.1, 117.5, 76.1, 57.0, 28.4, 26.7; **HRMS** (ESI) calcd for $C_{24}H_{20}NO_2$ $[M+H]^+$ 354.1489, found: 354.1488.

**10B-methyl-11-(naphthalen-1-yl)-10b,11-dihydro-6*H*-isoindolo[2,1-*a*]indol-6-one (3af)**

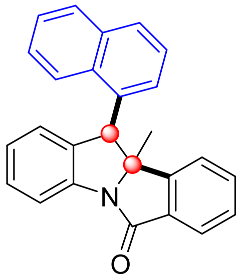


The product was purified by column chromatography (petroleum ether/EtOAc = 20:1). White solid (44.8 mg, 62% yield), melting point: 218-220 °C. **$^1$H NMR** (400 MHz, $CDCl_3$, ppm): δ 8.45 (d, $J$ = 8.4 Hz, 1 H), 7.88 (d, $J$ = 8.0 Hz, 1 H), 7.77-7.69 (m, 2 H), 7.60 (d, $J$ = 6.8 Hz, 1 H), 7.54-7.40 (m, 3 H), 7.19-7.17 (m, 2 H), 7.10-7.0 (m, 1 H), 6.920-6.86 (m, 2 H), 6.72 (d, $J$ =9.6 Hz, 1 H), 6.05 (d, $J$ =8.4 Hz, 1 H), 5.33 (s, 1 H), 1.92 (s, 3 H); **$^{13}$C NMR** (100 MHz, $CDCl_3$, ppm) : δ 169.0, 148.3, 140.1, 139.2, 137.1, 133.6, 132.7, 131.6, 131.5, 129.3, 128.9, 128.2, 127.3, 126.8, 126.5, 126.2, 125.7, 125.6, 125.4, 124.2, 123.0, 122.6, 117.4, 51.3, 28.5. **HRMS** (ESI) calcd for $C_{26}H_{20}NO$ $[M+H]^+$ 362.1540, found: 362.1539.

**10B-methyl-11-(naphthalen-2-yl)-10b,11-dihydro-6*H*-isoindolo[2,1-*a*]indol-6-one (3ag)**

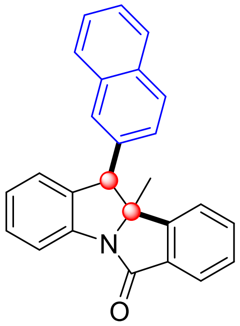

The product was purified by column chromatography (petroleum ether/EtOAc = 20:1). White solid (54.2 mg, 75% yield), melting point: 178-180 °C. **$^1$H NMR** (400 MHz, $CDCl_3$, ppm): δ 7.88 (d, $J$ =8.0 Hz, 1 H), 7.70-7.66 (m, 1 H), 7.63-7.56 (m, 2 H), 7.45-7.41 (m, 1 H), 7.37-7.30 (m, 3 H), 7.22-7.05 (m, 6 H), 6.52-6.49 (m, 1 H), 4.53 (s, 1 H), 1.81 (s, 3 H); **$^{13}$C NMR** (100 MHz, $CDCl_3$, ppm): δ 168.7, 148.8, 139.4, 139.1, 138.4, 133.1, 133.0, 132.3, 132.3, 129.0, 128.3, 128.3, 127.8, 127.6, 126.8, 126.5, 126.1, 125.8, 125.8, 125.2, 124.4, 123.1, 117.3, 76.1, 57.4, 29.0. **HRMS** (ESI) calcd for $C_{26}H_{20}NO$ $[M+H]^+$ 362.1540, found: 362.1541.

**(10bS,11R)-11-(1-ethoxyvinyl)-10b-methyl-10b,11-dihydro-6*H*-isoindolo[2,1-a]indol-6-one (3ah)**

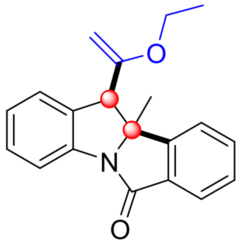

The product was purified by column chromatography (petroleum ether/EtOAc = 20:1). White solid (50.0 mg, 82% yield), melting point: 156-157 °C. **$^1$H NMR** (600 MHz, $CDCl_3$, ppm): δ 7.82 (d, $J$ =7.8 Hz, 1 H), 7.71 (m, $J$ =7.8 Hz, 1 H), 7.55-7.53 (m, 1 H), 7.48-7.43 (m, 2 H), 7.38-7.35 (m, 1 H), 7.28-7.26 (m, 1 H), 7.14-7.11 (m, 1 H), 3.75 (s, 1 H), 3.54-3.50 (m, 2 H), 2.99-2.93 (m, 2 H), 1.64 (s, 3 H), 0.80-0.77 (m, 3 H); **$^{13}$C NMR** (150 MHz, $CDCl_3$, ppm): δ 168.9, 161.2, 148.9, 140.1, 136.6, 133.6, 131.6, 128.8, 128.4, 126.2, 124.6, 124.2, 123.2, 117.3, 83.82, 74.8, 62.6, 56.4, 27.9, 13.9. **HRMS** (ESI) calcd for $C_{20}H_{20}NO_2$ $[M+H]^+$ 305.1416, found: 305.1416

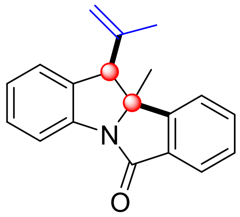

**(10bS,11R)-10b-methyl-11-(prop-1-en-2-yl)-10b,11-dihydro-6H-isoindolo[2,1-a]indol-6-one (3ai)**

The product was purified by column chromatography (petroleum ether/EtOAc = 20:1). White solid (46.8 mg, 85% yield), melting point: 162-163 °C. **$^1$H NMR** (600 MHz, $CDCl_3$, ppm): δ 7.85 (d, $J$ =7.2 Hz, 1 H), 7.75 (d, $J$ =7.8 Hz, 1 H), 7.58-7.56 (m, 1 H), 7.49-7.46 (m, 1 H), 7.41-7.35 (m, 2 H), 7.21 (d, $J$ =7.8 Hz, 1 H), 7.14-7.12 (m, 1 H), 4.78 (s, 1 H), 4.58 (s, 1 H), 3.88 (s, 1 H), 1.63 (s, 3 H), 0.75 (s, 3 H); **$^{13}$C NMR** (150 MHz, $CDCl_3$, ppm): δ 168.0, 149.0, 144.6, 139.3, 137.6, 133.4, 132.3, 128.7, 128.6, 126.3, 124.8, 124.8, 123.2, 116.9, 114.0, 74.4, 59.2, 29.3, 18.9. **HRMS** (ESI) calcd for $C_{19}H_{18}NO$ $[M+H]^+$ 275.1310, found: 275.1311

**(10bS,11R)-10b-methyl-11-vinyl-10b,11-dihydro-6H-isoindolo[2,1-a]indol-6-one (3aj)**

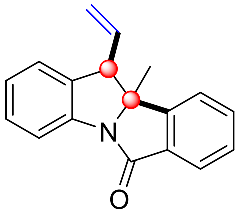

The product was purified by column chromatography (petroleum ether/EtOAc = 20:1). White solid (41.2 mg, 79% yield), melting

point: 145-147 °C. **$^1$H NMR** (600 MHz, $CDCl_3$, ppm): δ 7.86 (d, *J* =7.2 Hz, 1 H), 7.72 (d, *J* =7.8 Hz, 1 H), 7.61-7.58 (m, 1 H), 7.49-7.46 (m, 1 H), 7.40-7.34 (m, 2 H), 7.24 (d, *J* =7.8 Hz, 1 H), 7.14-7.12 (m, 1 H), 5.15-5.09 (m, 1 H), 4.83-4.73 (m, 2 H), 3.78 (d, *J* =8.4 Hz, 1 H),1.64 (s, 3 H); **$^{13}$C NMR** (150 MHz, $CDCl_3$, ppm): δ 168.2, 148.7, 138.7, 137.7, 136.9, 133.3, 132.5, 128.7, 128.7, 126.7, 124.8, 124.8, 123.4, 117.3, 115.8, 75.0, 55.0, 27.6. **HRMS** (ESI) calcd for $C_{18}H_{16}NO$ $[M+H]^+$ 261.1154, found: 261.1154

**(10bS,11R)-11-butyl-10b-methyl-10b,11-dihydro-6H-isoindolo[2,1-a]indol-6-one (3ak)**

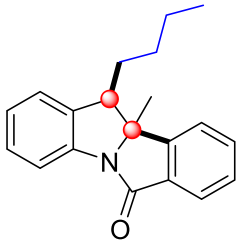


The product was purified by column chromatography (petroleum ether/EtOAc = 20:1). White solid (47.1 mg, 81% yield), melting point: 181-183 °C. **$^1$H NMR** (600 MHz, $CDCl_3$, ppm): δ 7.86 (d, *J* =6.6 Hz, 1 H), 7.68 (d, *J* =7.8 Hz, 1 H), 7.62-7.59 (m, 1 H), 7.49-7.46 (m, 2 H), 7.32-7.29 (m, 1 H), 7.23 (d, *J* =7.8 Hz, 1 H), 7.10-7.07 (m, 1 H), 3.20-3.10 (m, 2 H), 1.65 (s, 3 H), 1.56-1.51 (m, 2 H), 1.36-1.30 (m, 2 H), 1.11-1.08 (m, 2 H), 0.90-0.88 (m, 3 H); **$^{13}$C NMR** (150 MHz, $CDCl_3$, ppm): δ 168.3, 151.4, 139.7, 135.9, 132.9, 132.8, 128.7, 128.1, 125.8, 125.1, 124.6, 122.0, 117.4, 72.0, 40.5, 29.1, 27.3, 13.8, 9.7. **HRMS** (ESI) calcd for $C_{20}H_{22}NO$ $[M+H]^+$ 291.1623, found: 291.1622

## NMR spectra for the palladium-catalyzed dearomatization products

$^{1}H$-NMR spectrum ($CDCl_3$, 400 MHz) of **3aa**

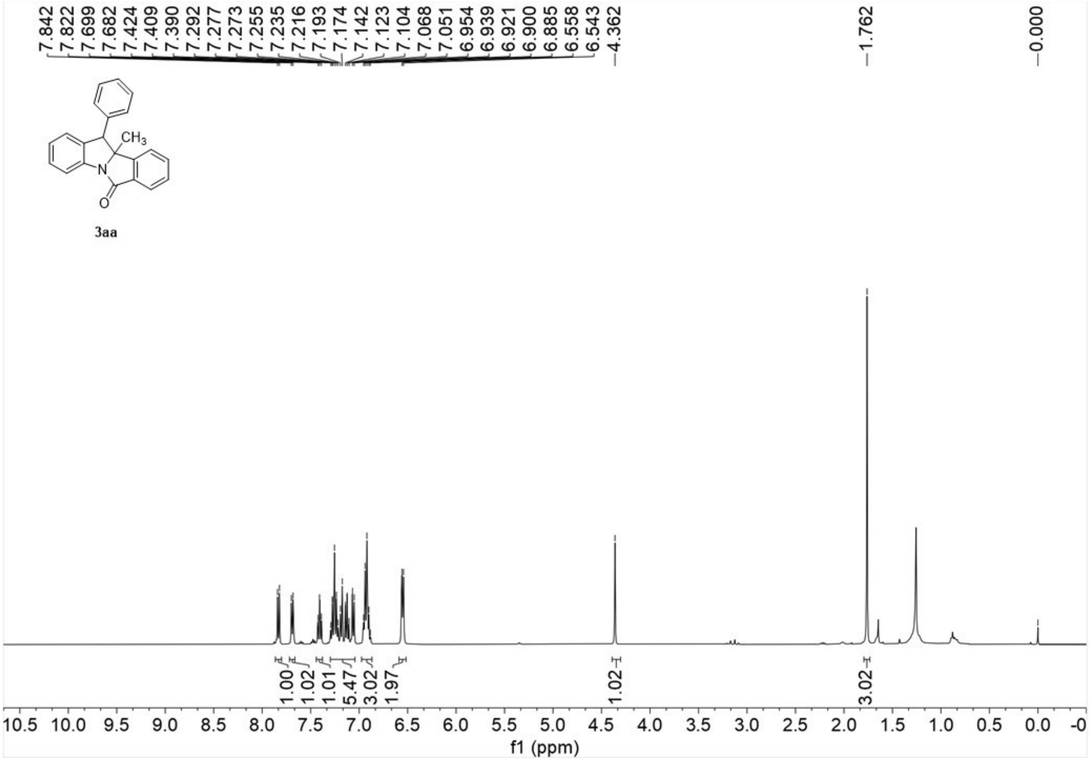


$^{13}C$-NMR spectrum ($CDCl_3$, 100 MHz) of **3aa**

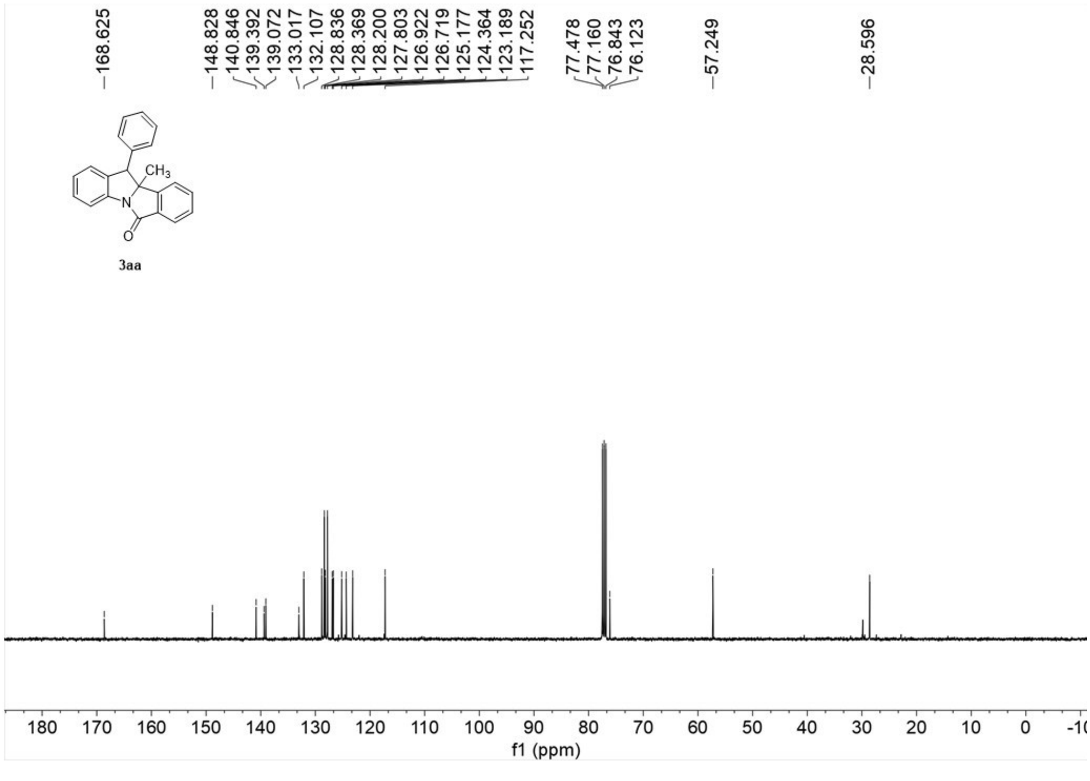

$^{1}$H-NMR spectrum ($CDCl_3$, 600 MHz) of **3ba**

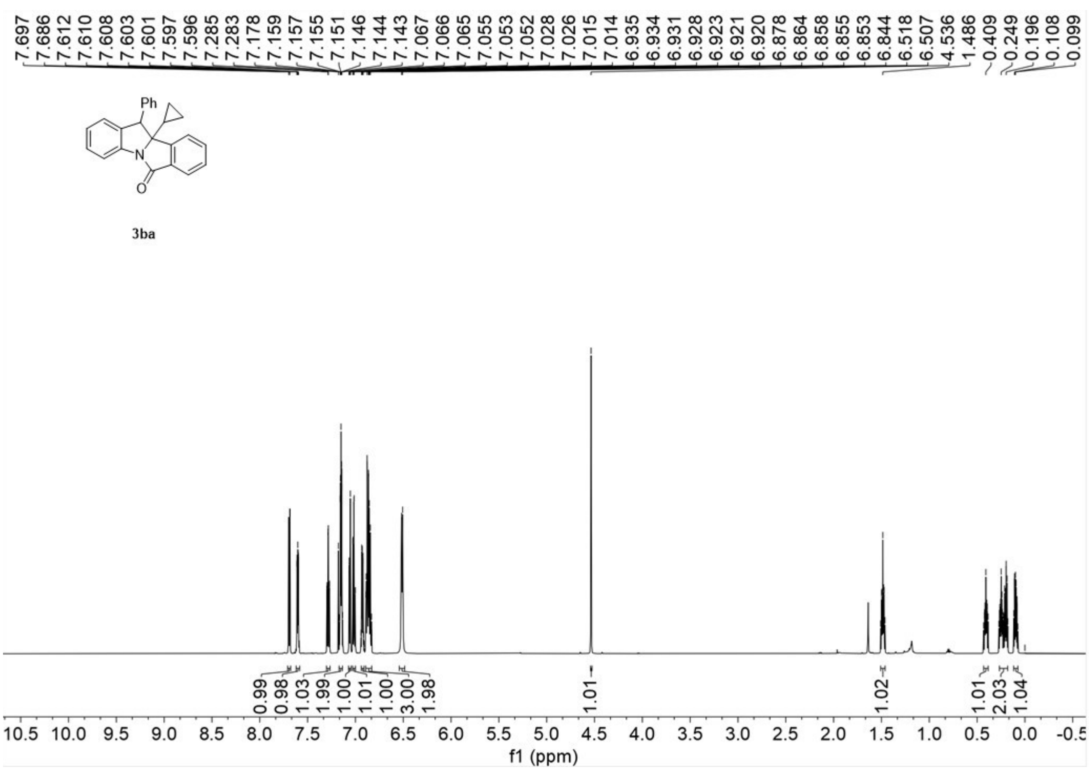


$^{13}$C-NMR spectrum ($CDCl_3$, 150 MHz) of **3ba**

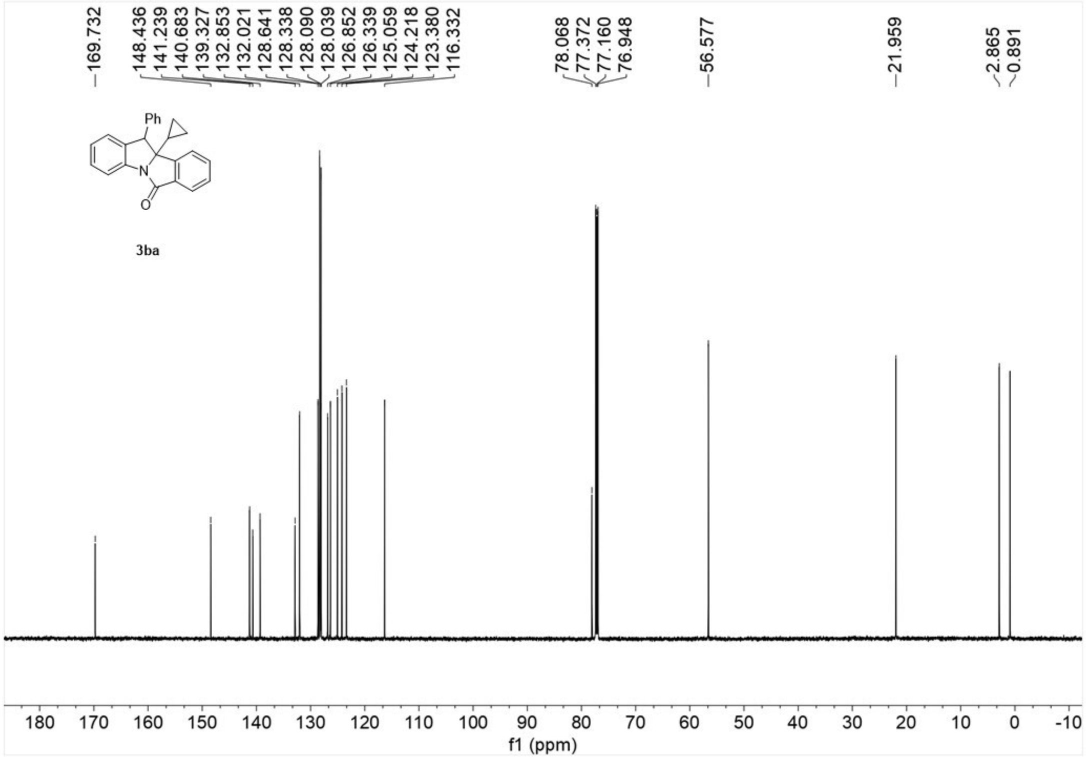

$^1$H-NMR spectrum ($CDCl_3$, 400 MHz) of **3ca**

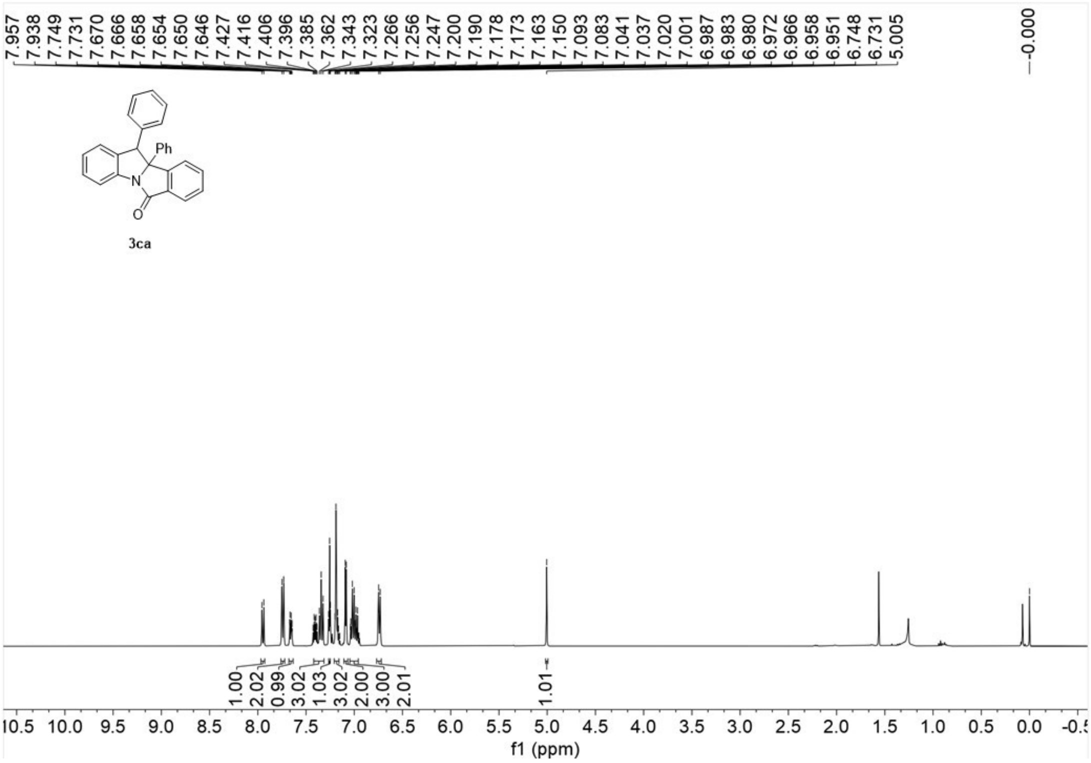

$^{13}$C-NMR spectrum ($CDCl_3$, 100 MHz) of **3ca**

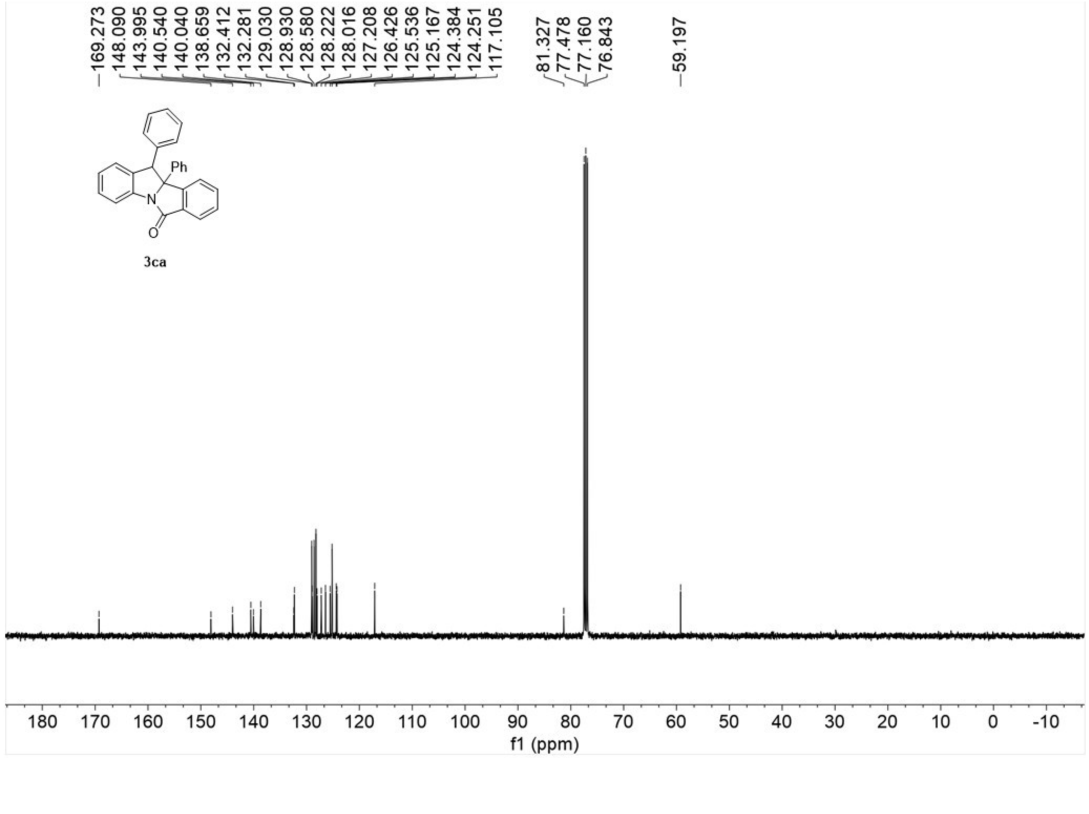

$^{1}$H-NMR spectrum ($CDCl_3$, 600 MHz) of **3da**

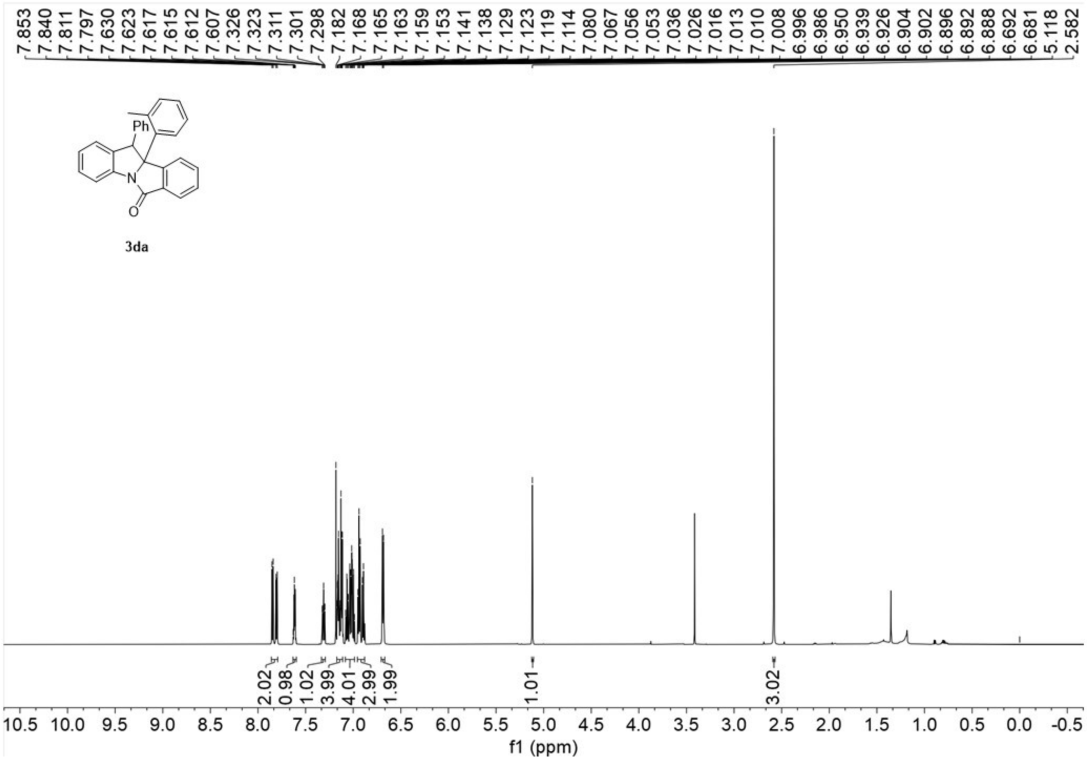


$^{13}$C-NMR spectrum ($CDCl_3$, 150 MHz) of **3da**

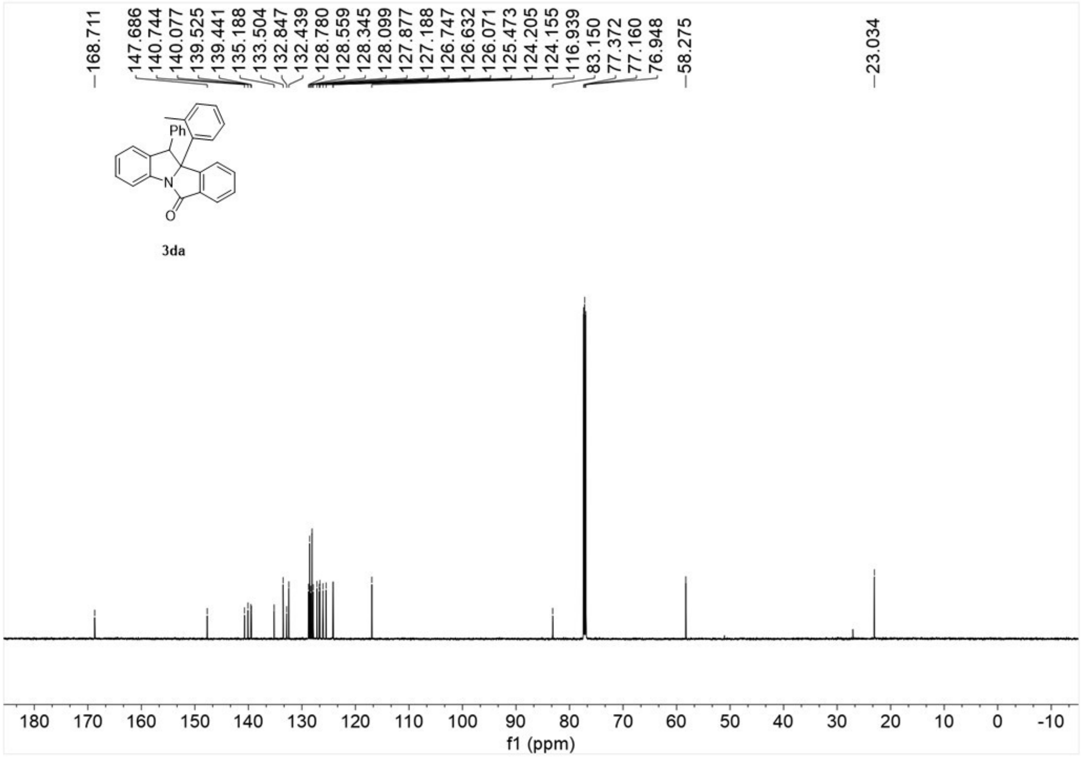

$^1$H-NMR spectrum ($CDCl_3$, 600 MHz) of **3ea**

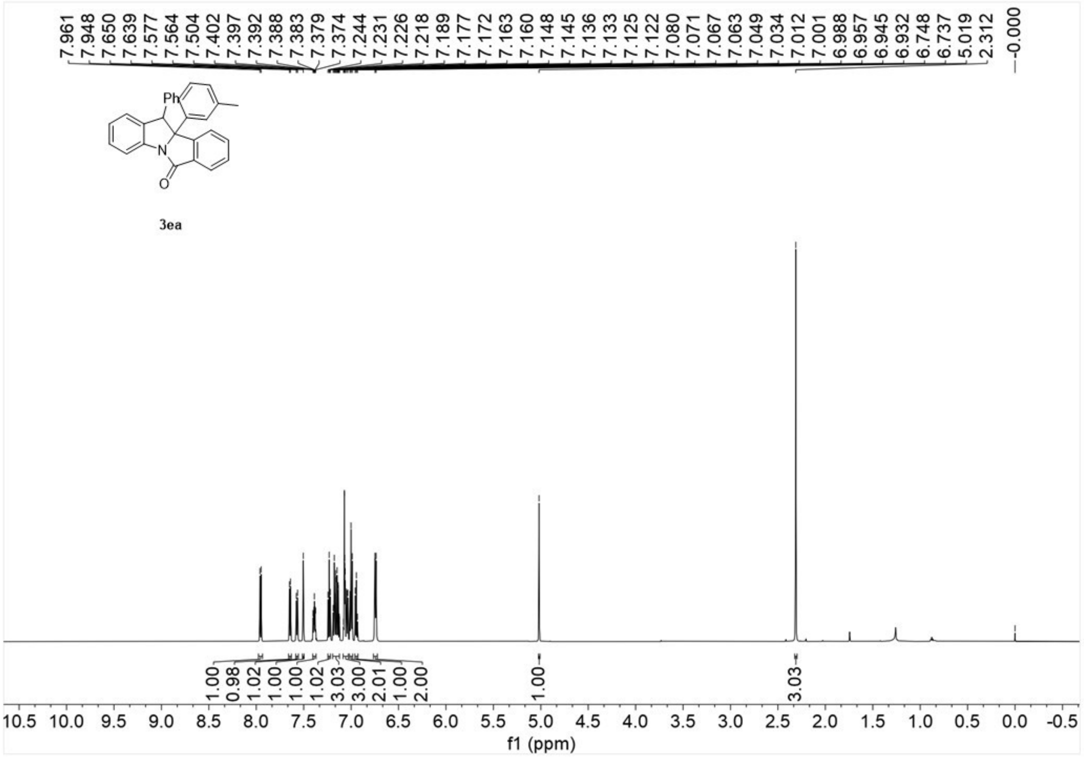

$^{13}$C-NMR spectrum ($CDCl_3$, 150 MHz) of **3ea**

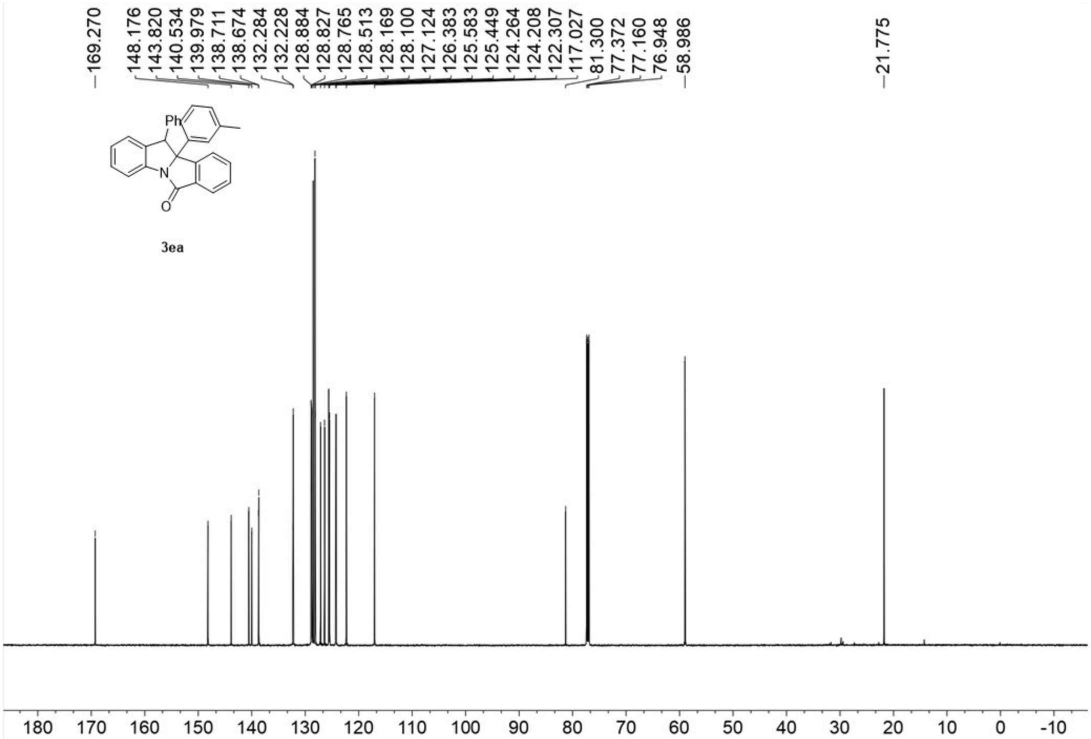

$^{1}$H-NMR spectrum ($CDCl_3$, 600 MHz) of **3fa**

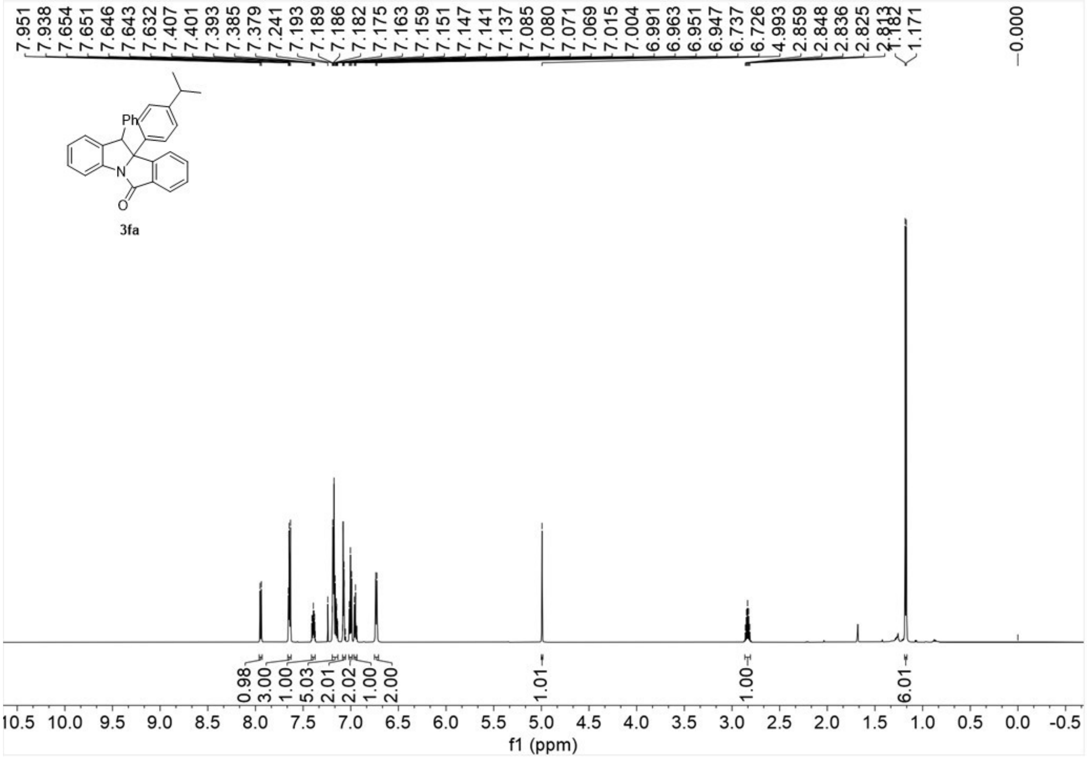

$^{13}$C-NMR spectrum ($CDCl_3$, 150 MHz) of **3fa**

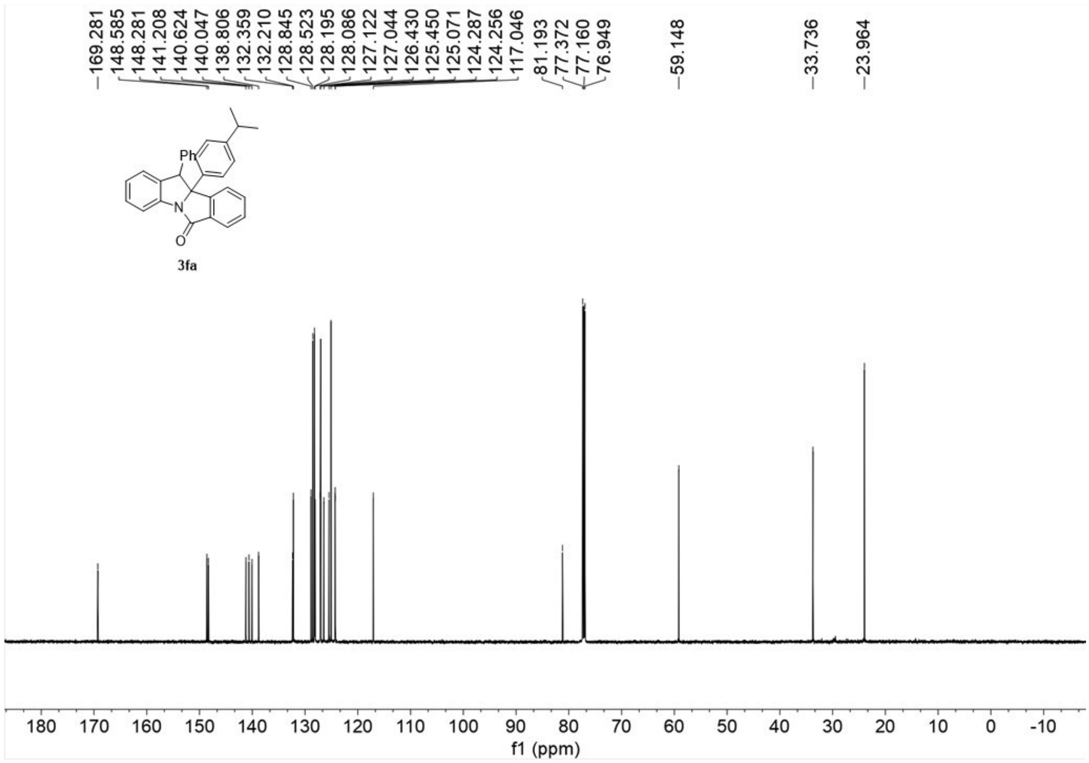

$^1$H-NMR spectrum ($CDCl_3$, 600 MHz) of **3ga**

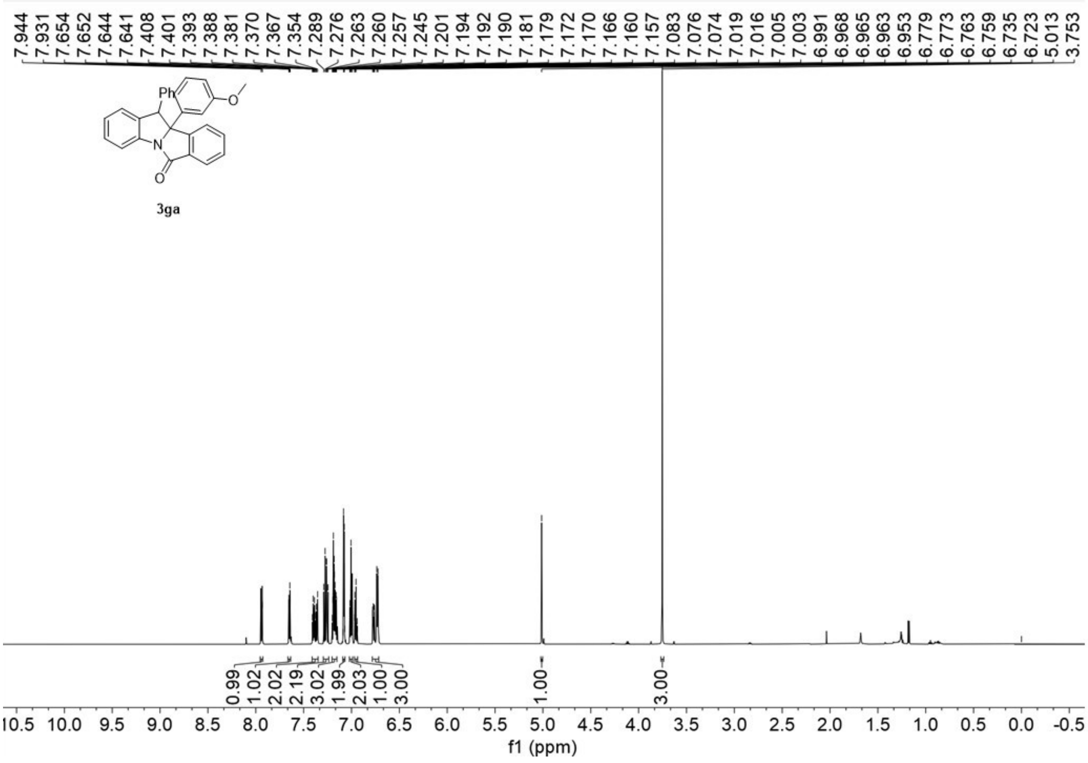

$^{13}$C-NMR spectrum ($CDCl_3$, 150 MHz) of **3ga**

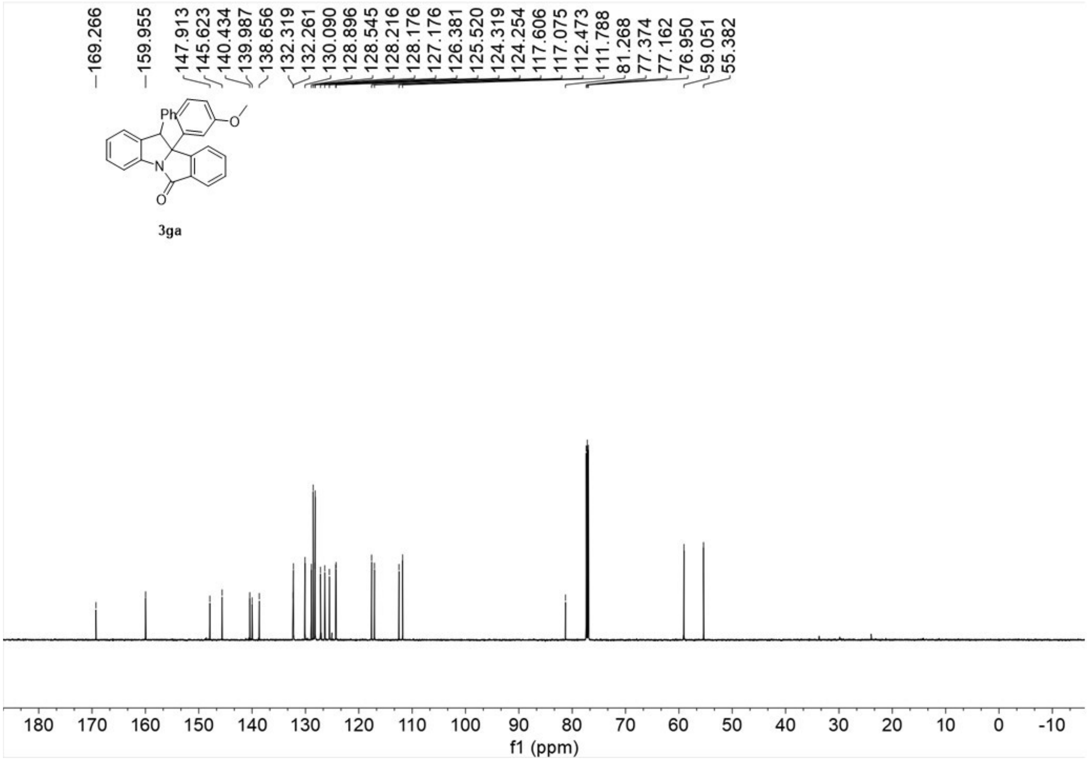

$^{1}$H-NMR spectrum ($CDCl_3$, 600 MHz) of **3ha**

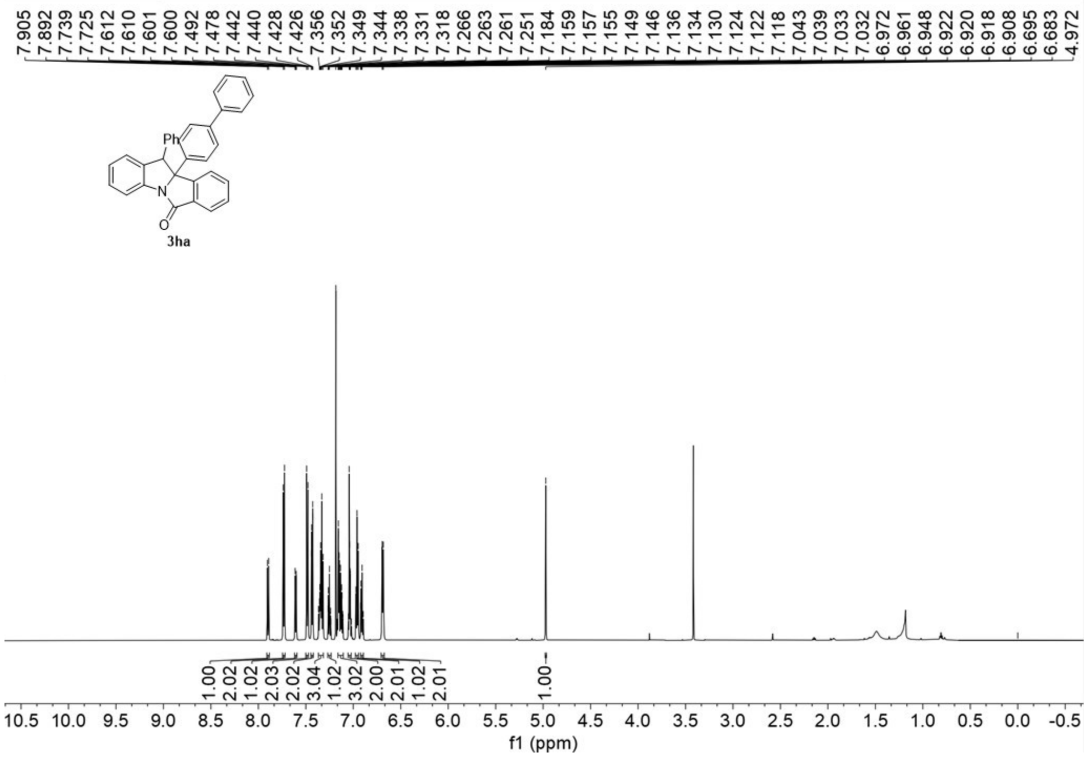


$^{13}$C-NMR spectrum ($CDCl_3$, 150 MHz) of **3ha**

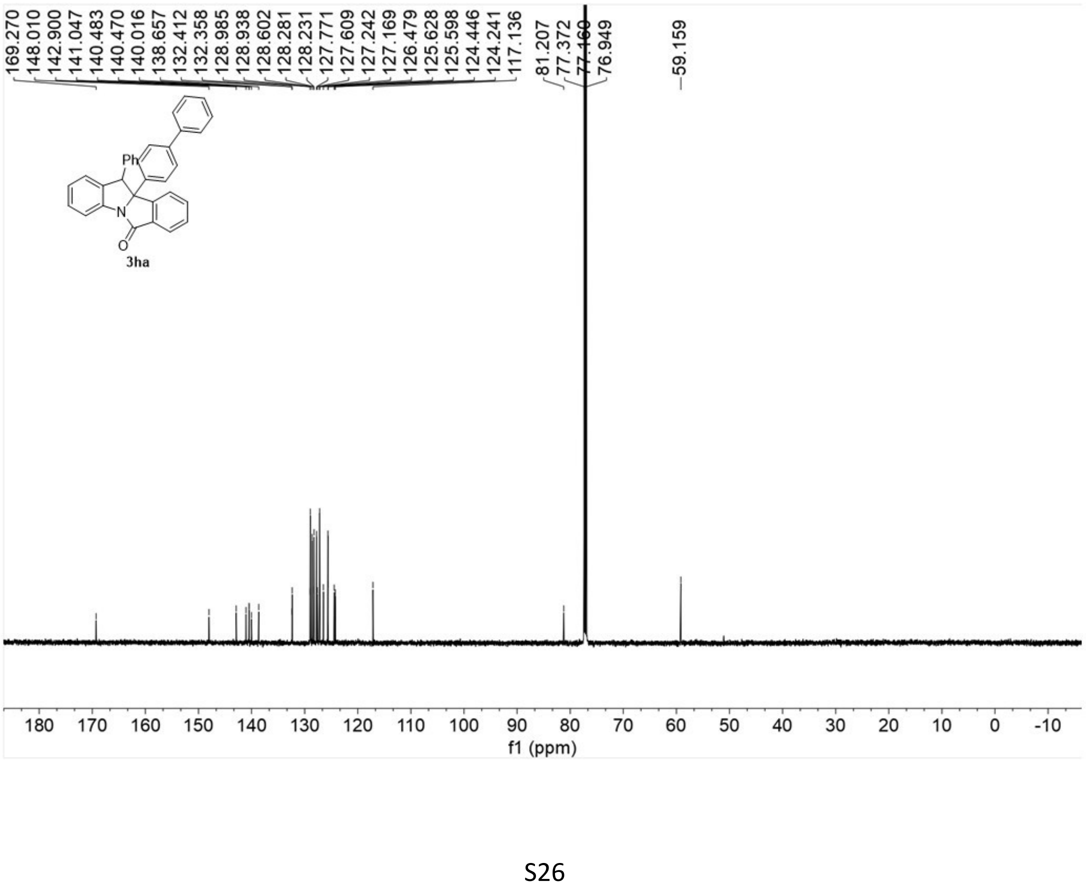

$^{1}$H-NMR spectrum ($CDCl_3$, 400 MHz) of **3ia**

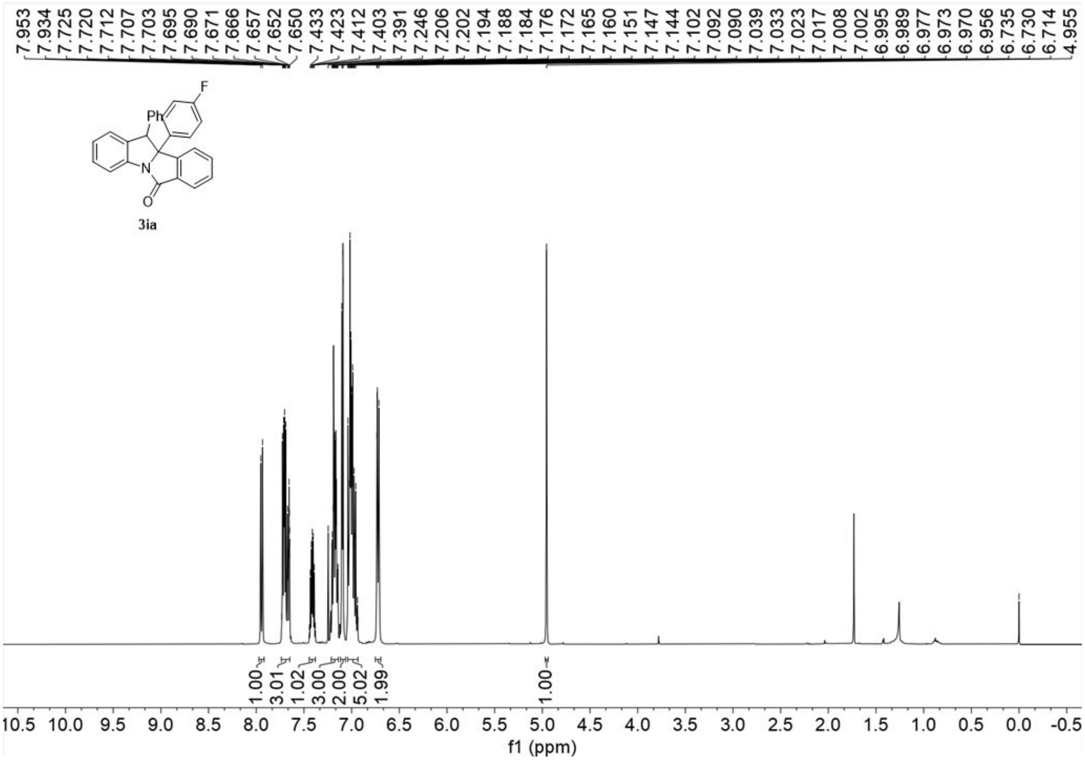


$^{13}$C-NMR spectrum ($CDCl_3$, 100 MHz) of **3ia**

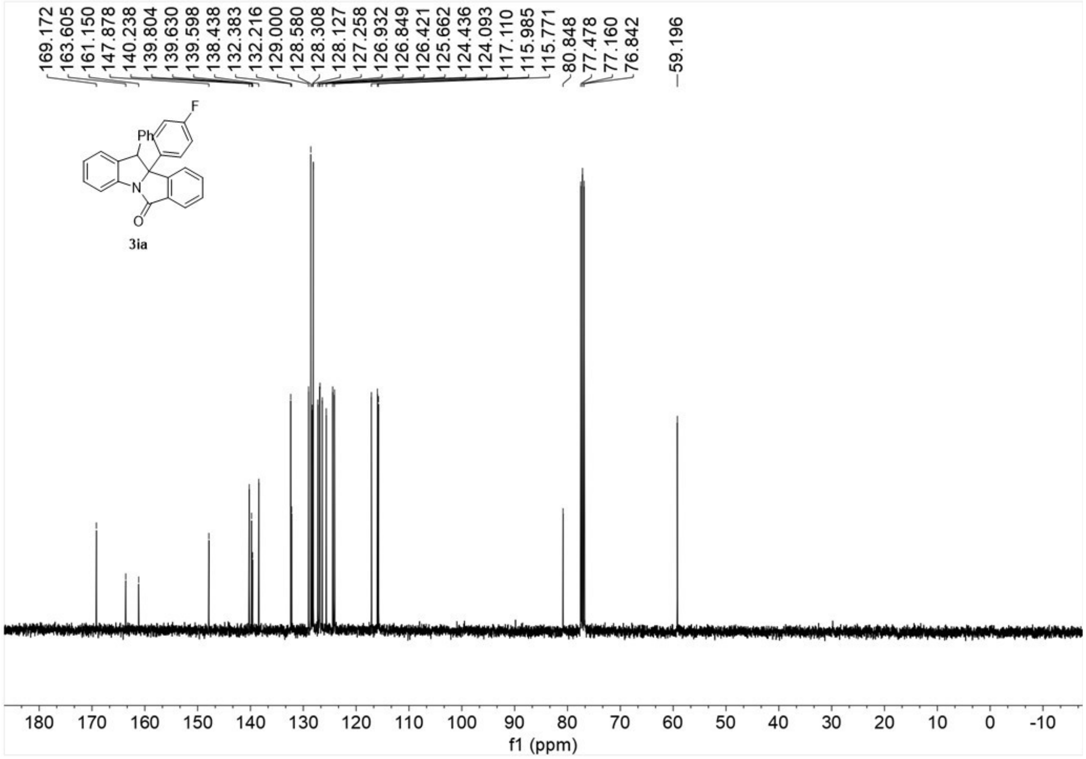

$^{19}$F-NMR spectrum ($CDCl_3$, 377 MHz) of **3ia**

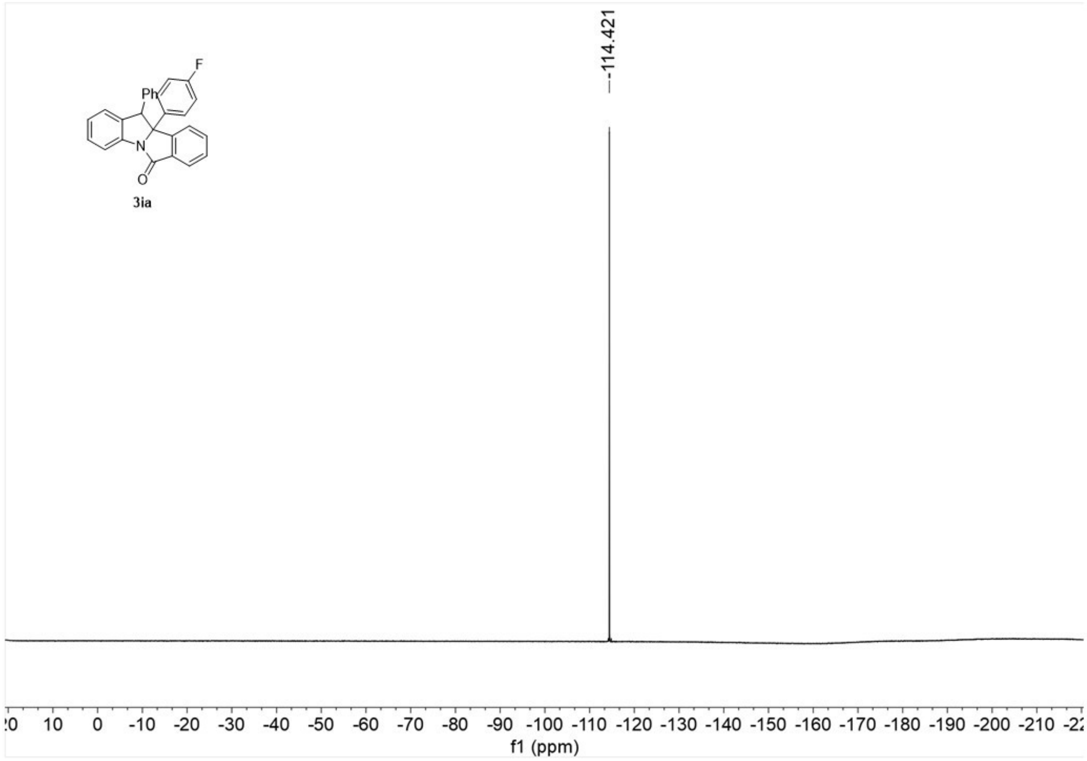

$^{1}$H-NMR spectrum ($CDCl_3$, 400 MHz) of **3ja**

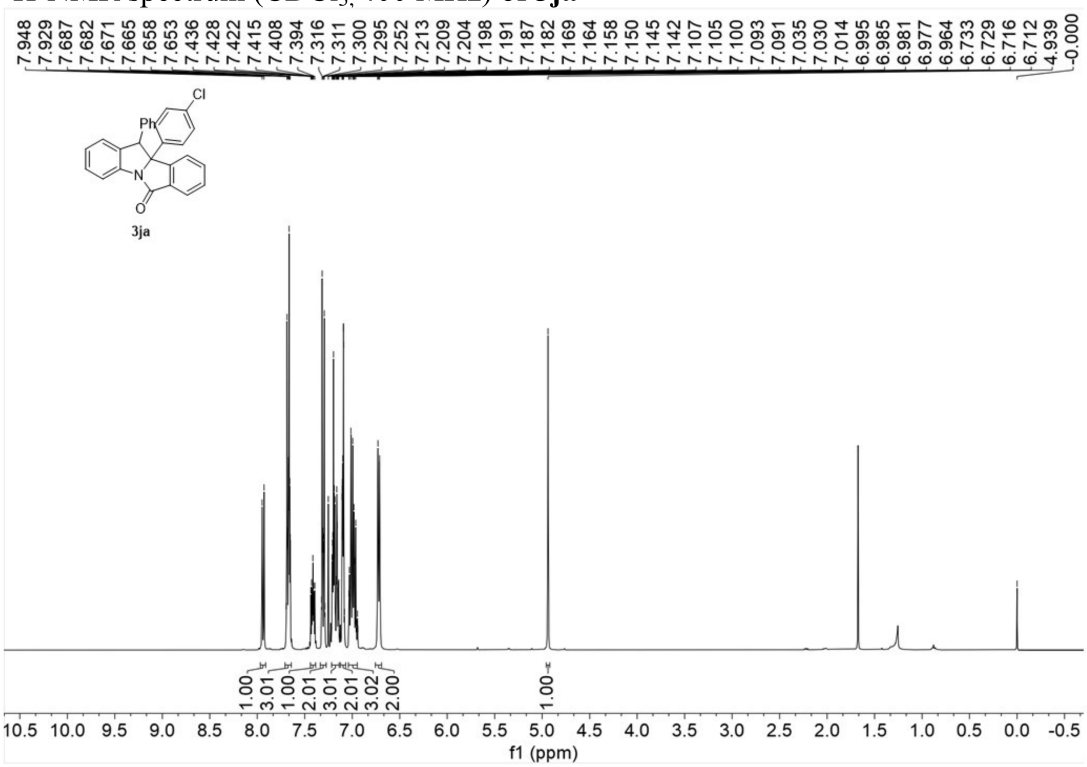

$^{13}$C-NMR spectrum ($CDCl_3$, 100 MHz) of **3ja**

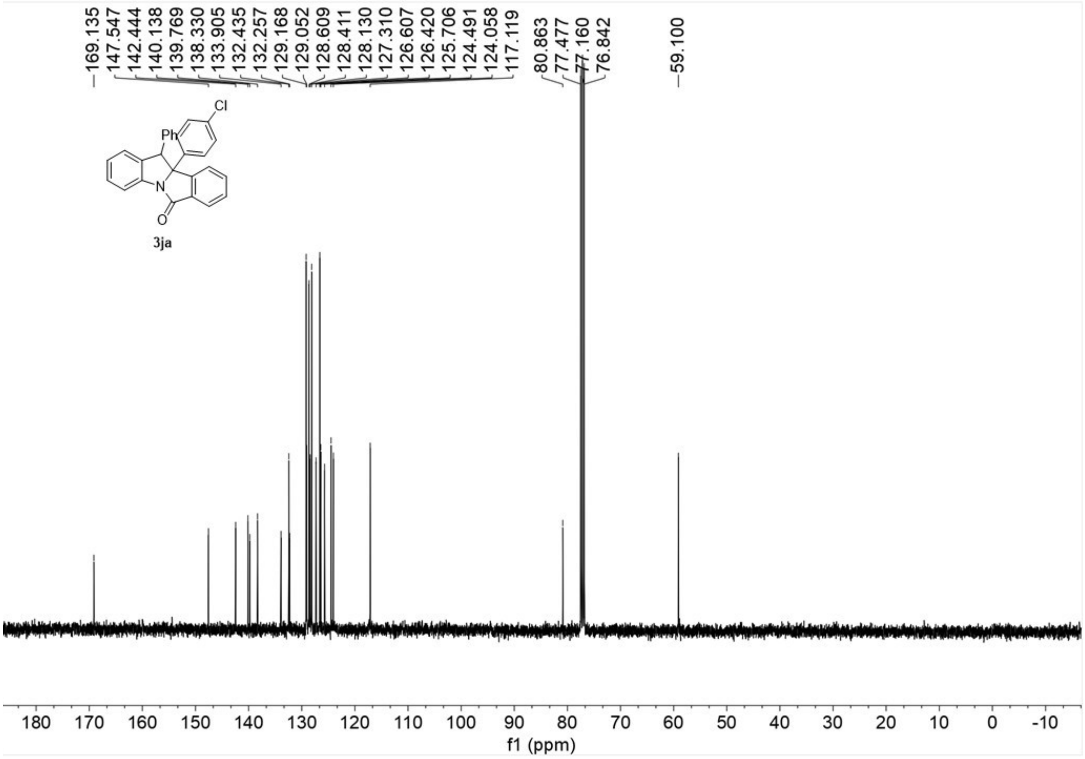


$^{1}$H-NMR spectrum ($CDCl_3$, 600 MHz) of **3ka**

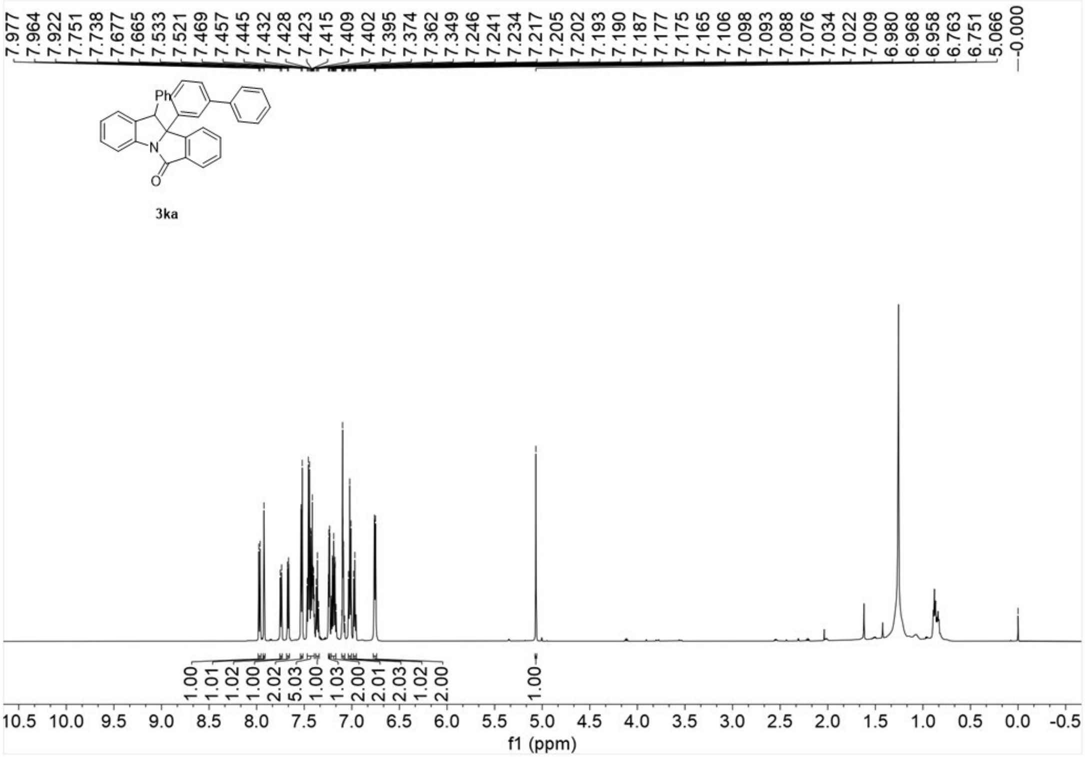

$^{13}$C-NMR spectrum ($CDCl_3$, 150 MHz) of **3ka**

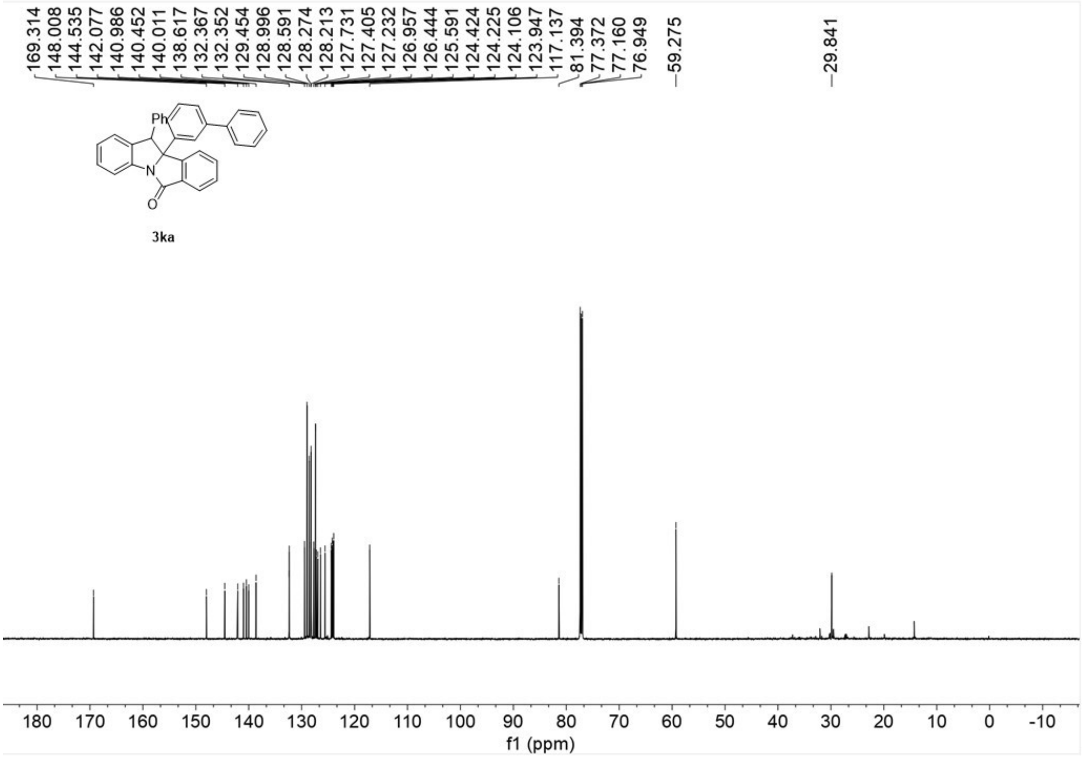


$^{1}$H-NMR spectrum ($CDCl_3$, 600 MHz) of **3la**

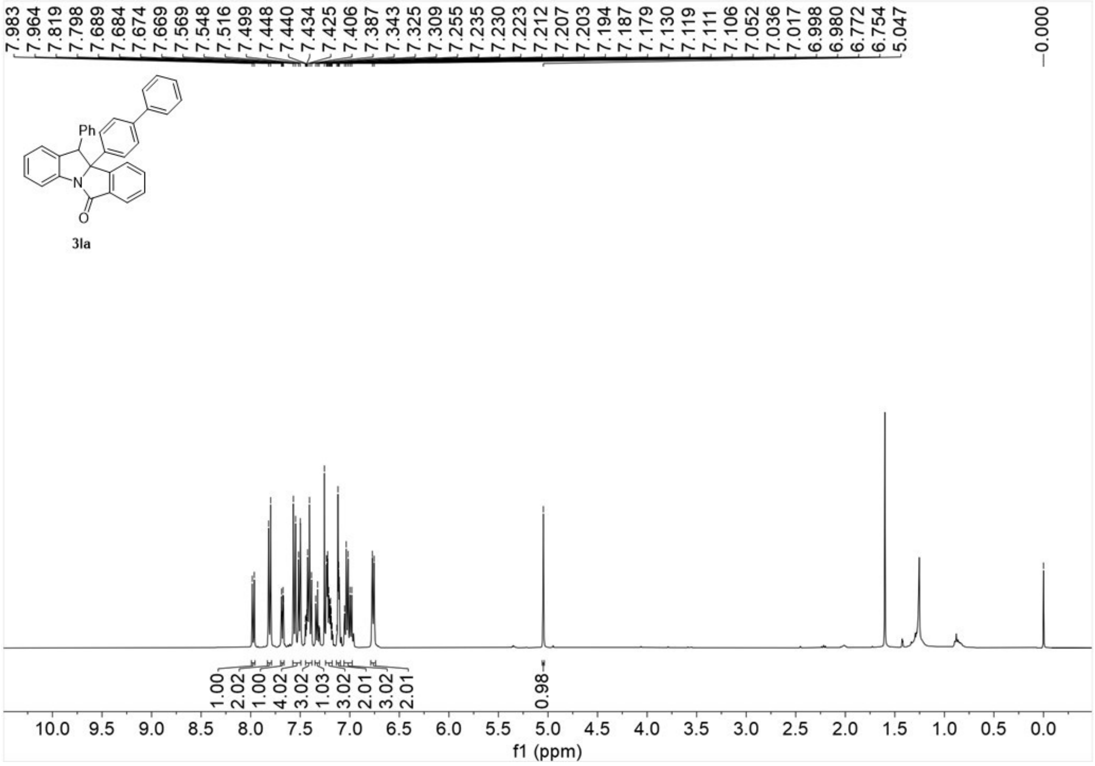

<sup></sup>

$^{13}$C-NMR spectrum ($CDCl_3$, 150 MHz) of **3la**

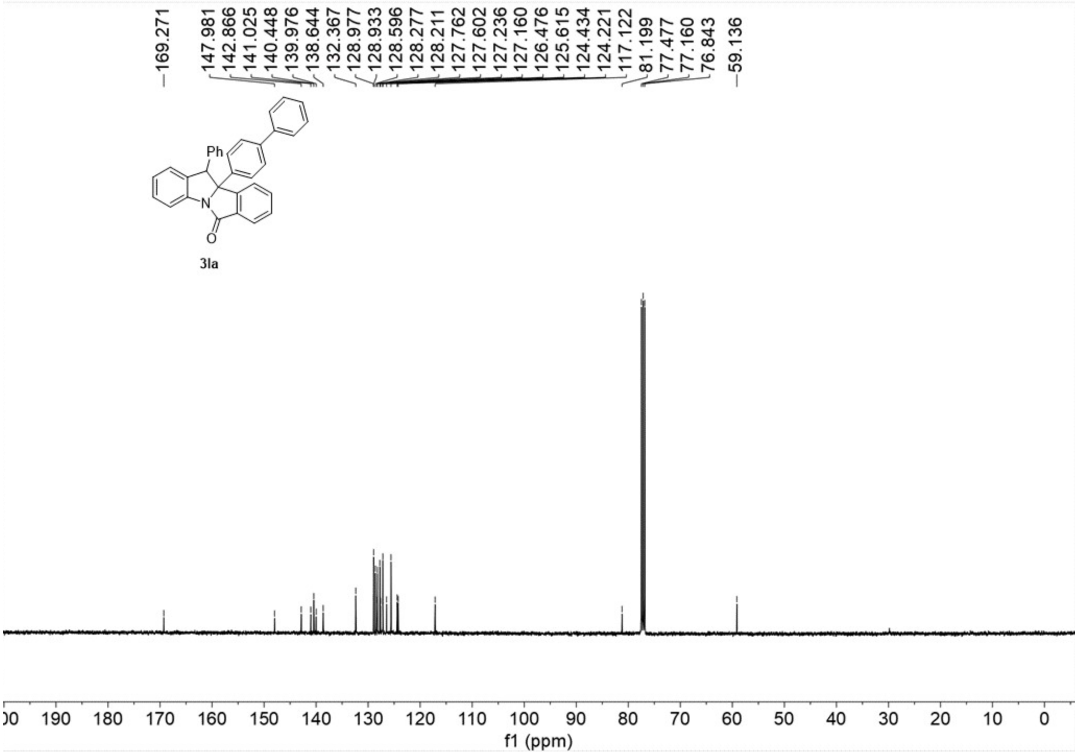

$^{1}$H-NMR spectrum ($CDCl_3$, 600 MHz) of **3ma**

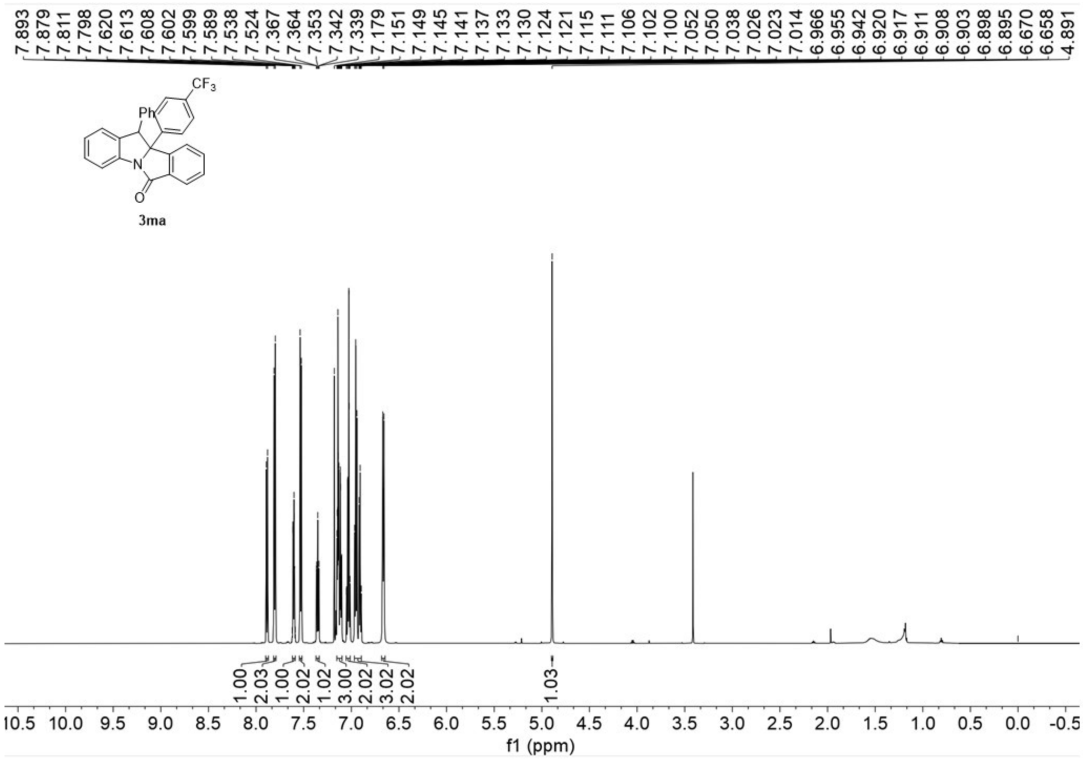

$^{13}$C-NMR spectrum ($CDCl_3$, 150 MHz) of **3ma**

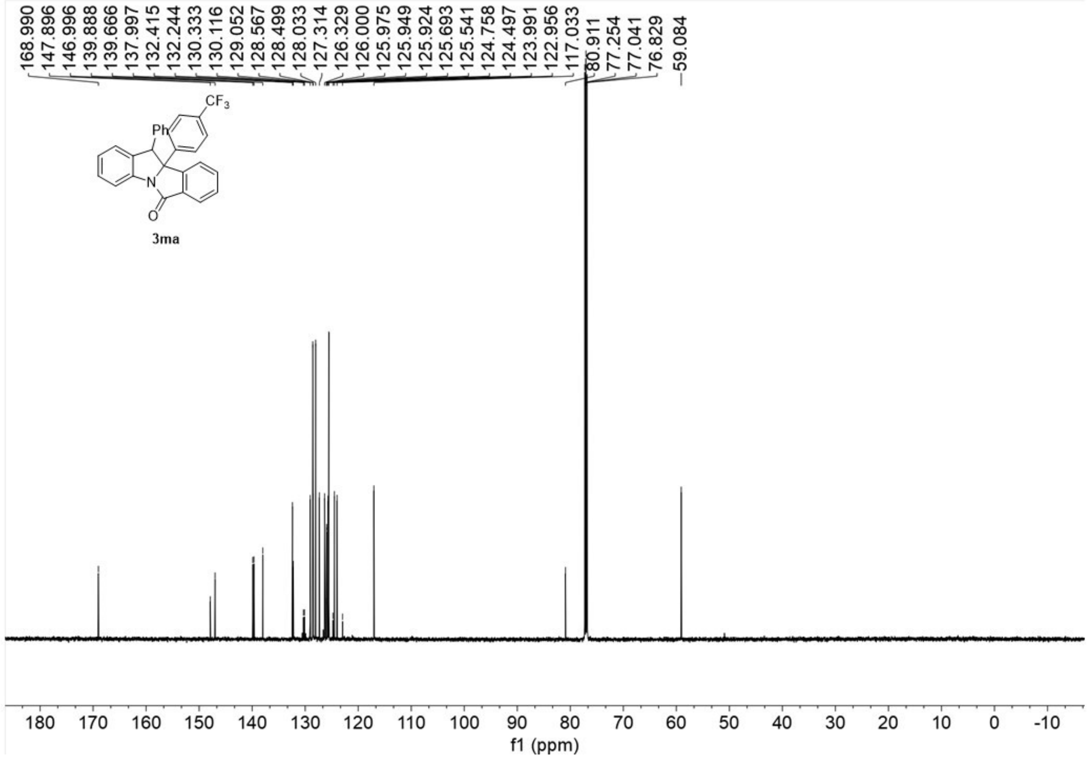

$^{19}$F-NMR spectrum ($CDCl_3$, 565 MHz) of **3ma**

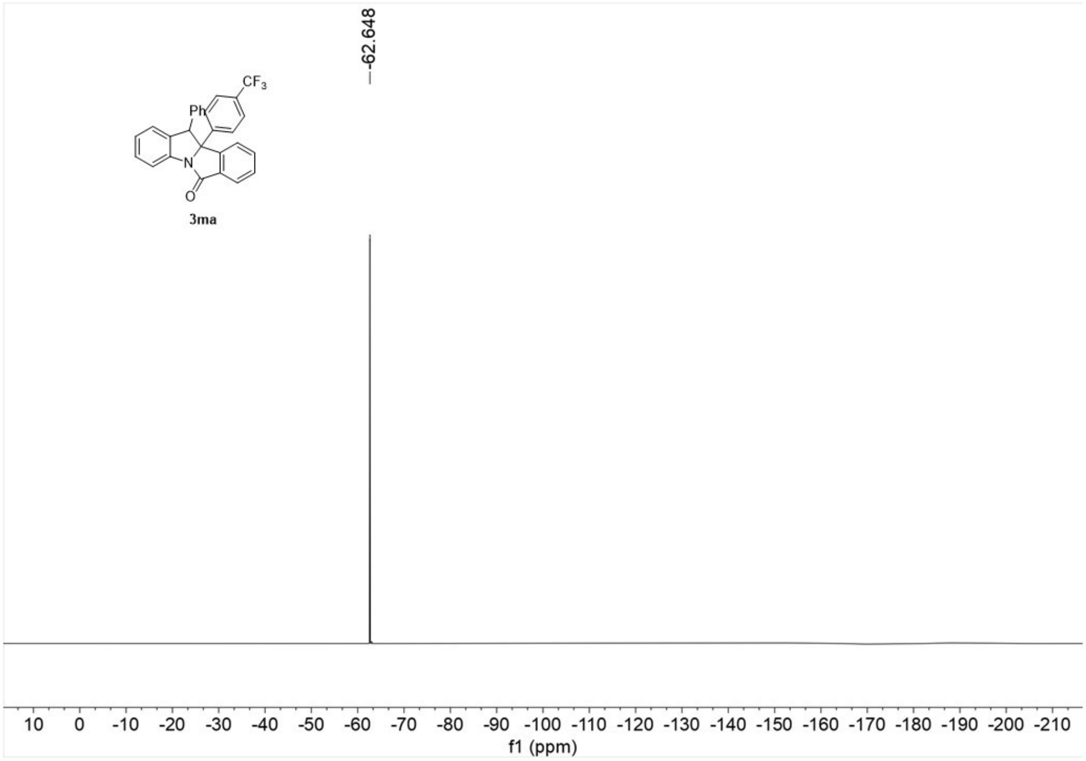


$^{1}$H-NMR spectrum ($CDCl_3$, 400 MHz) of **3na**

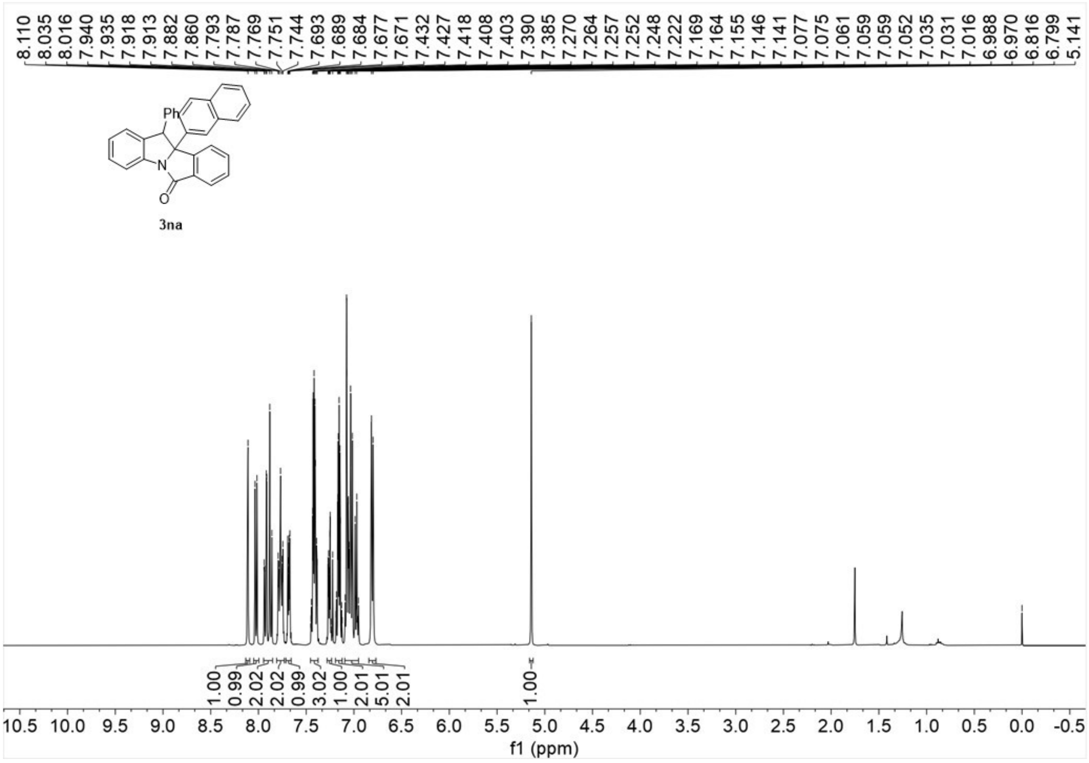

$^{13}$C-NMR spectrum ($CDCl_3$, 100 MHz) of **3na**

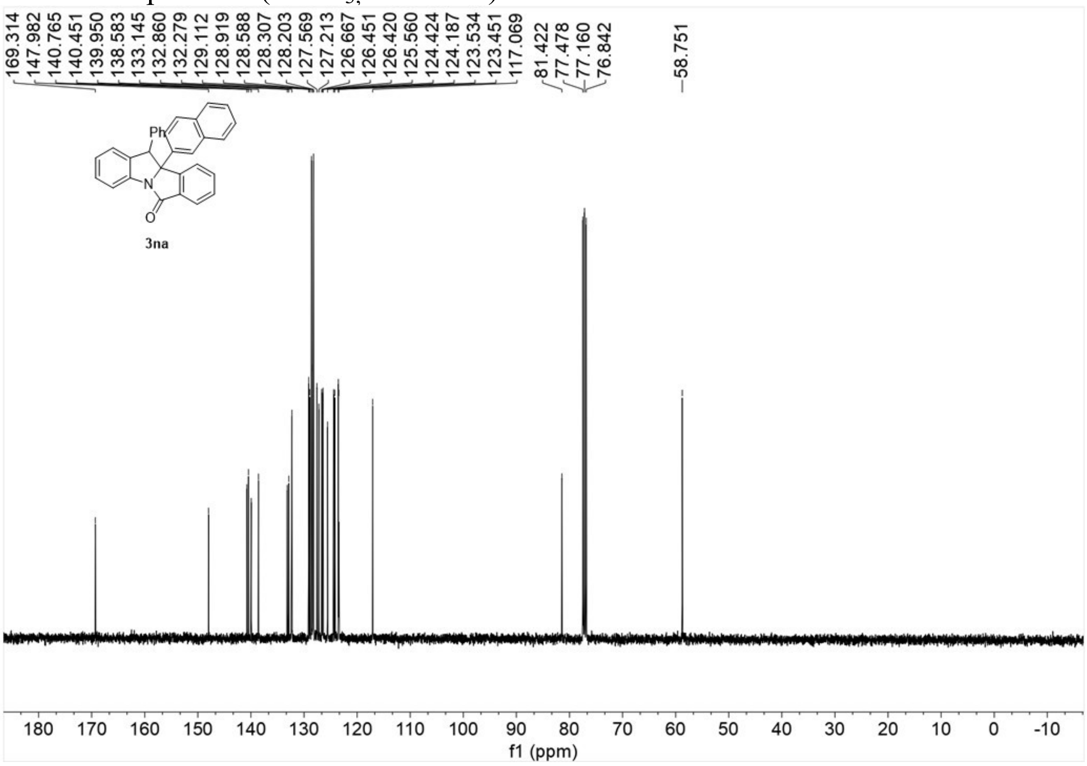


$^{1}$H-NMR spectrum ($CDCl_3$, 400 MHz) of **3oa**

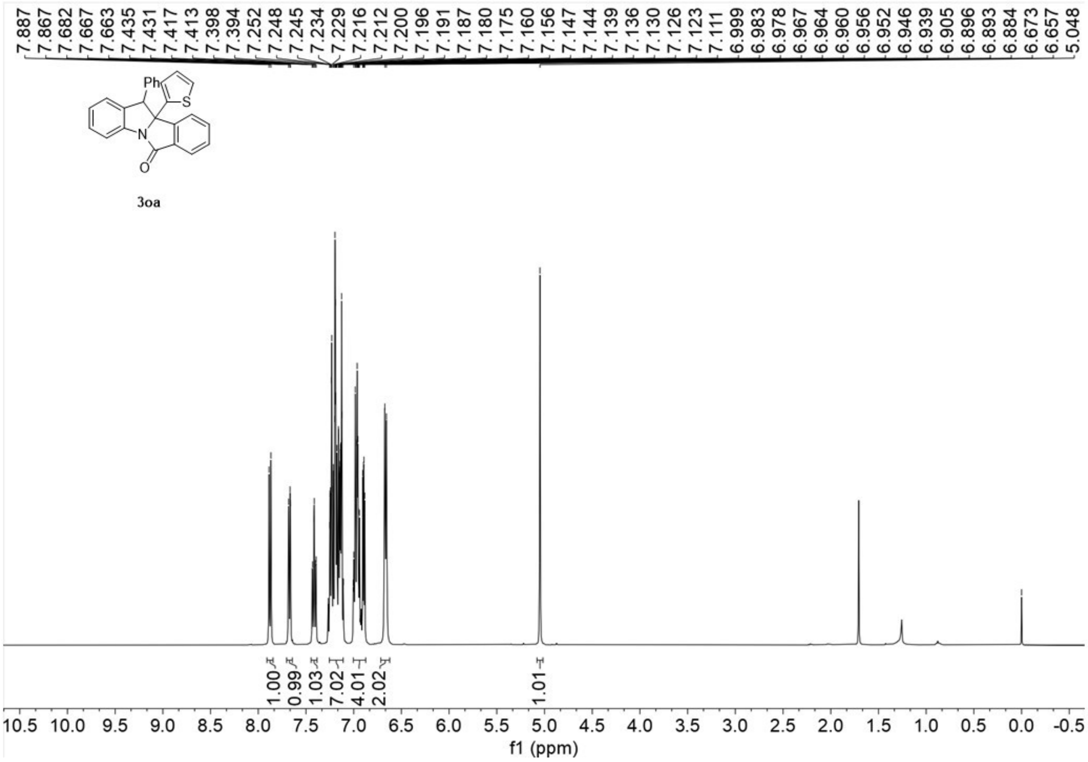

$^{13}$C-NMR spectrum ($CDCl_3$, 100 MHz) of **3oa**

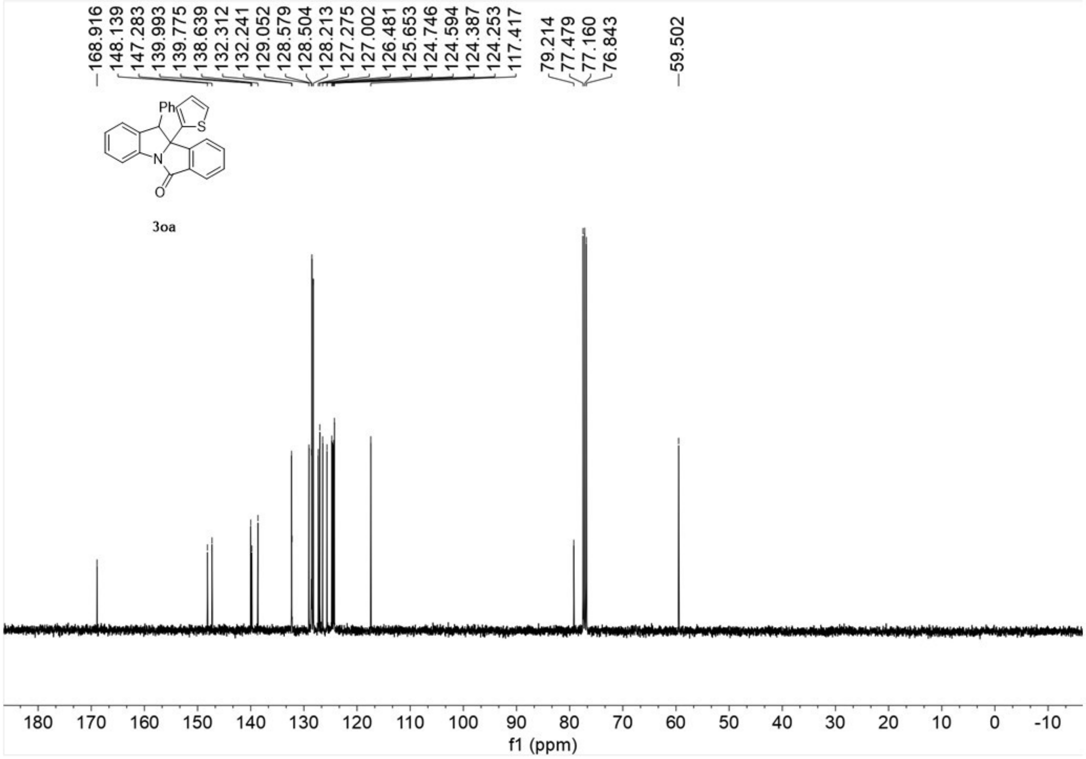

$^{1}$H-NMR spectrum ($CDCl_3$, 400 MHz) of **3pa**

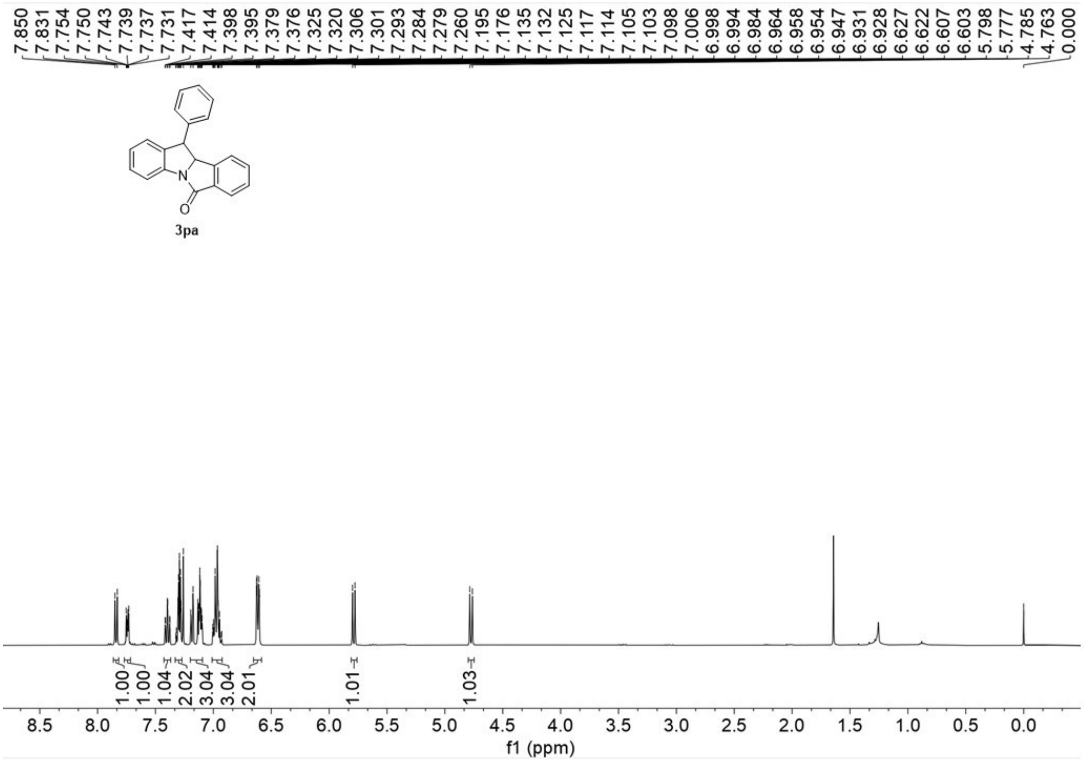

$^{13}$C-NMR spectrum ($CDCl_3$, 100 MHz) of **3pa**

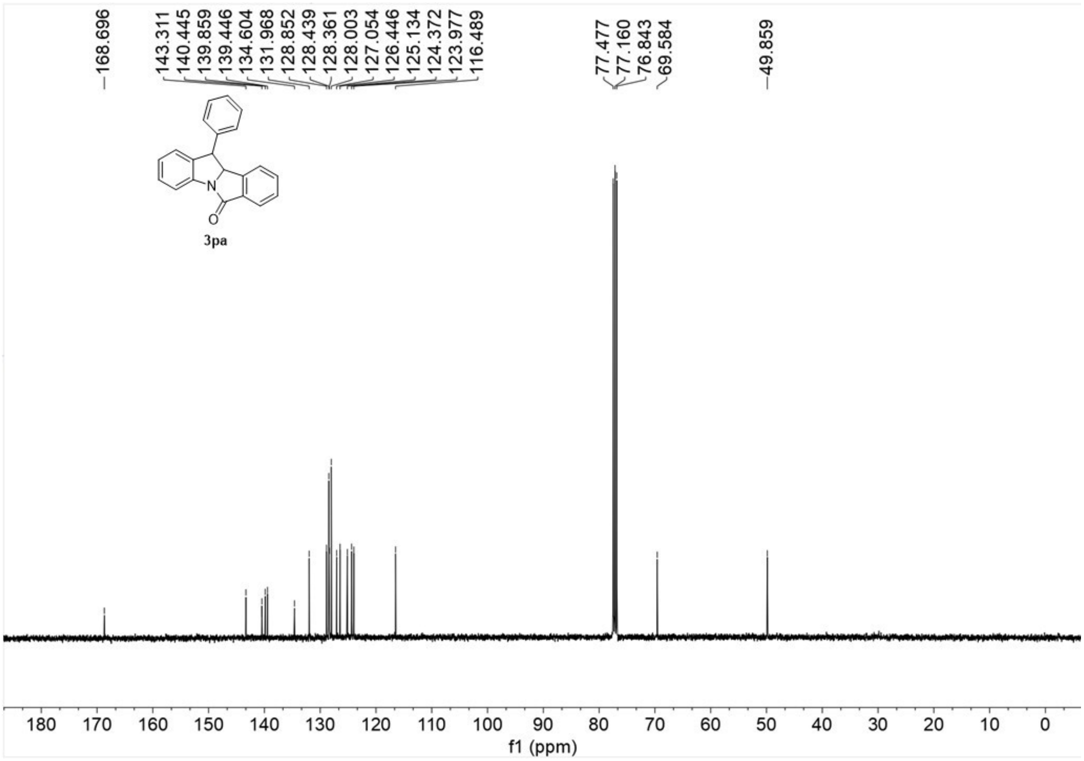


$^{1}$H-NMR spectrum ($CDCl_3$, 400 MHz) of **3qa**

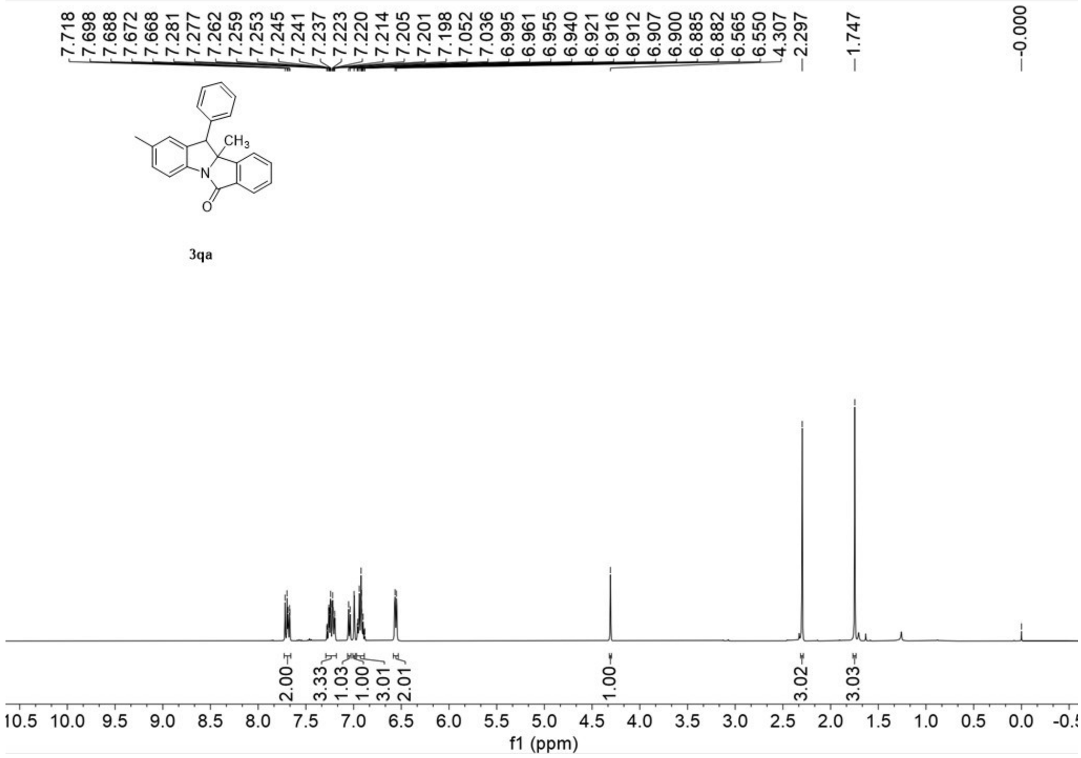

$^{13}$C-NMR spectrum ($CDCl_3$, 100 MHz) of **3qa**

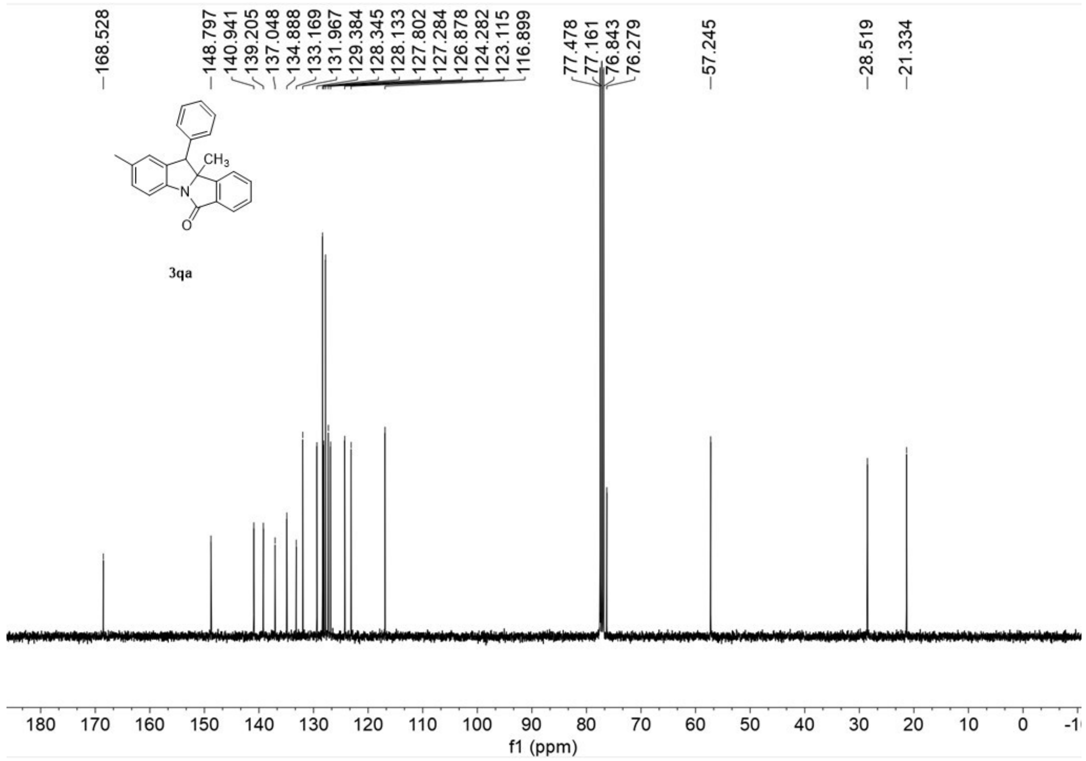


$^{1}$H-NMR spectrum ($CDCl_3$, 400 MHz) of **3ra**

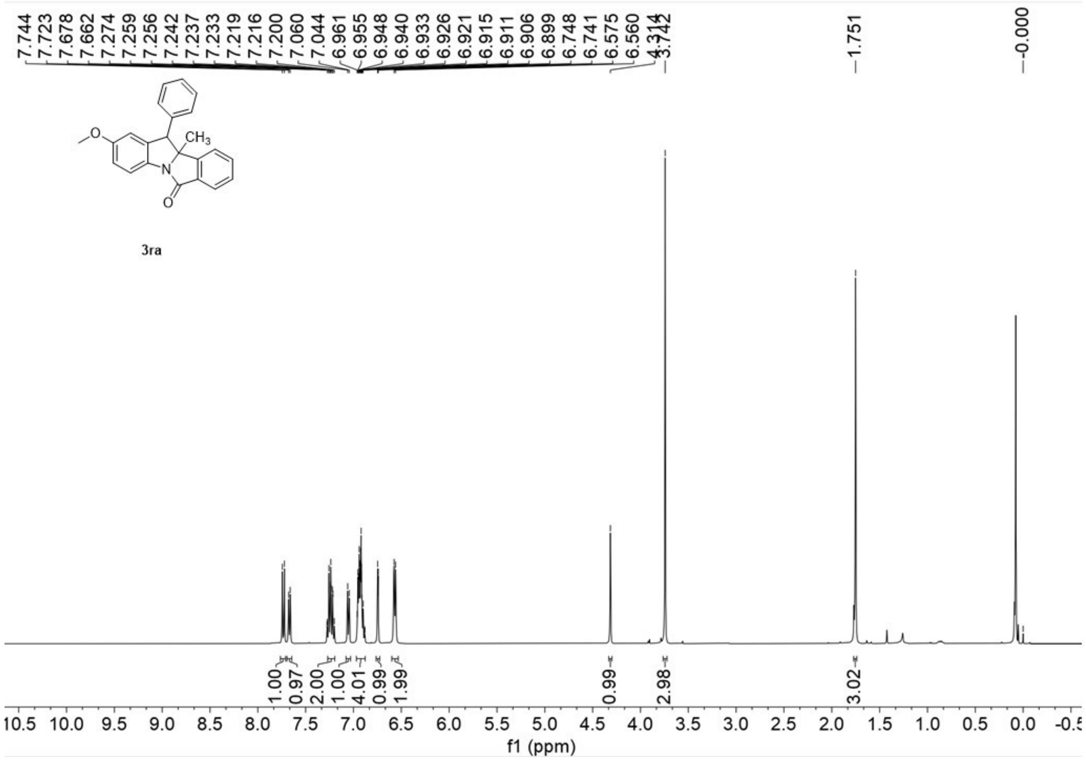

$^{13}$C-NMR spectrum ($CDCl_3$, 100 MHz) of **3ra**

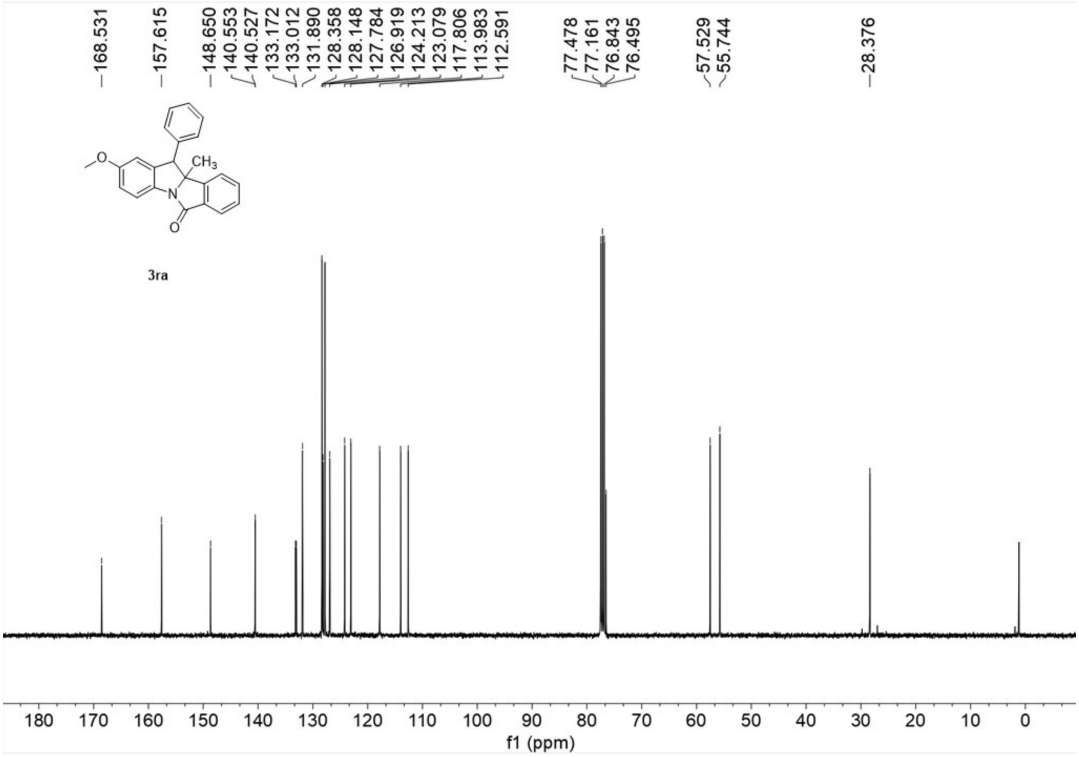

$^{1}$H-NMR spectrum ($CDCl_3$, 400 MHz) of **3sa**

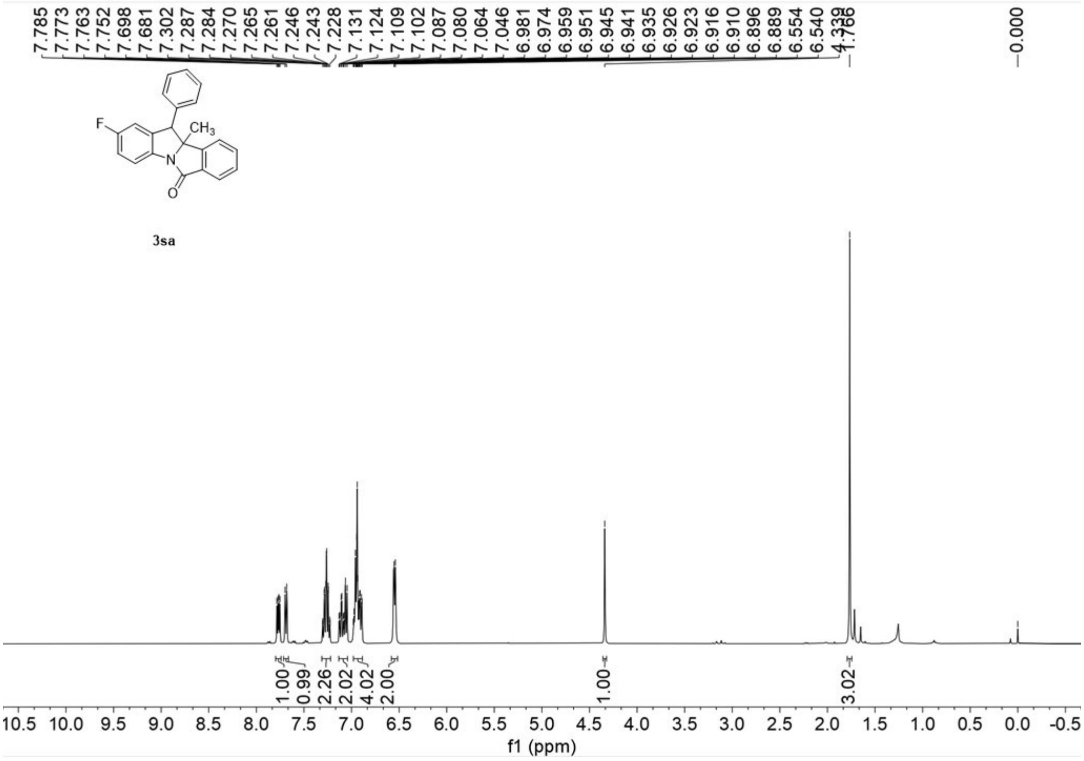

$^{13}$C-NMR spectrum ($CDCl_3$, 100 MHz) of **3sa**

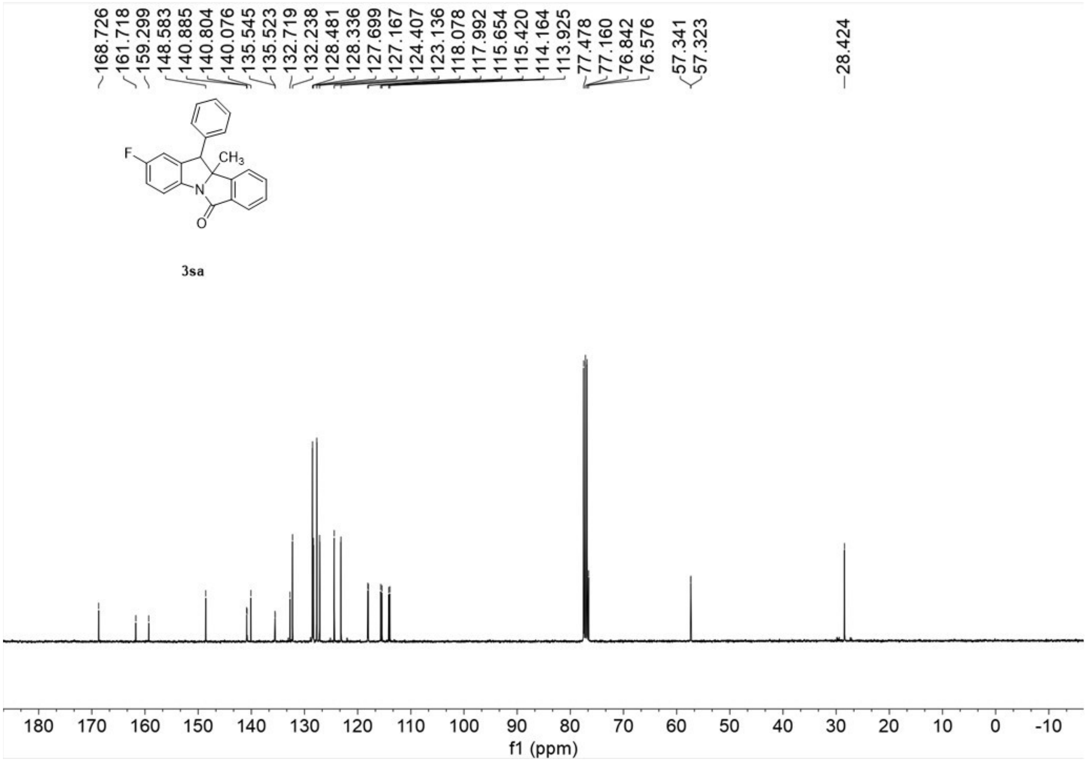


$^{19}$F-NMR spectrum ($CDCl_3$, 377 MHz) of **3sa**

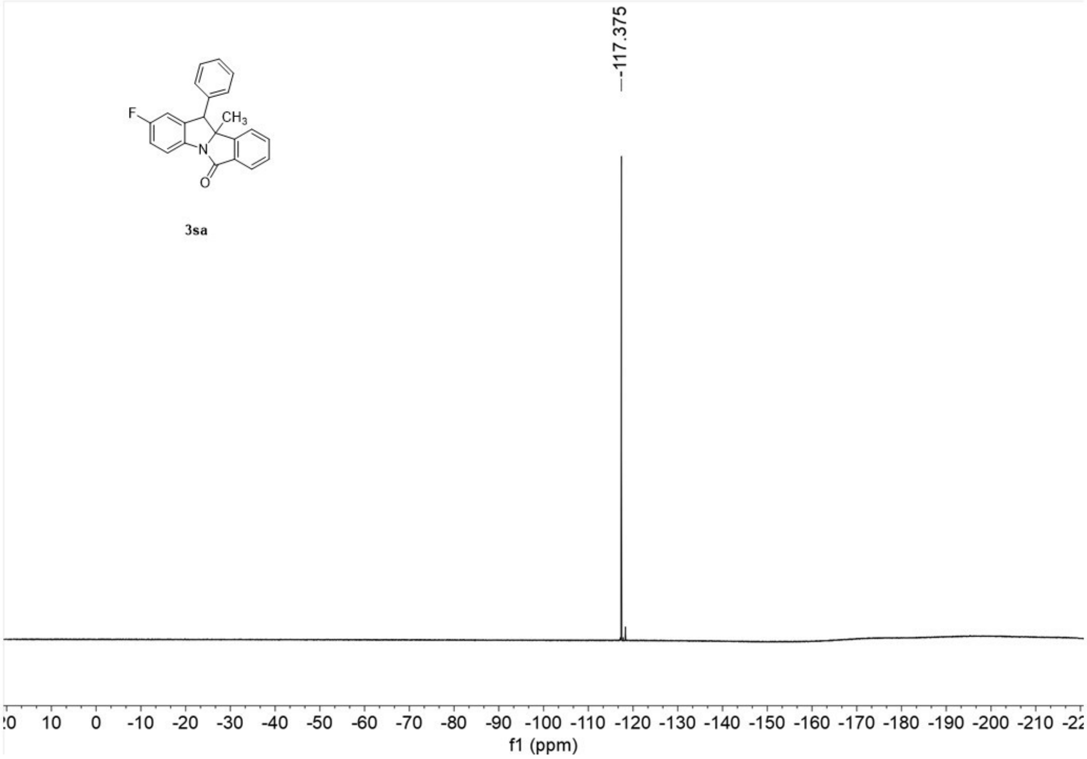

$^{1}$H-NMR spectrum ($CDCl_3$, 400 MHz) of **3ta**

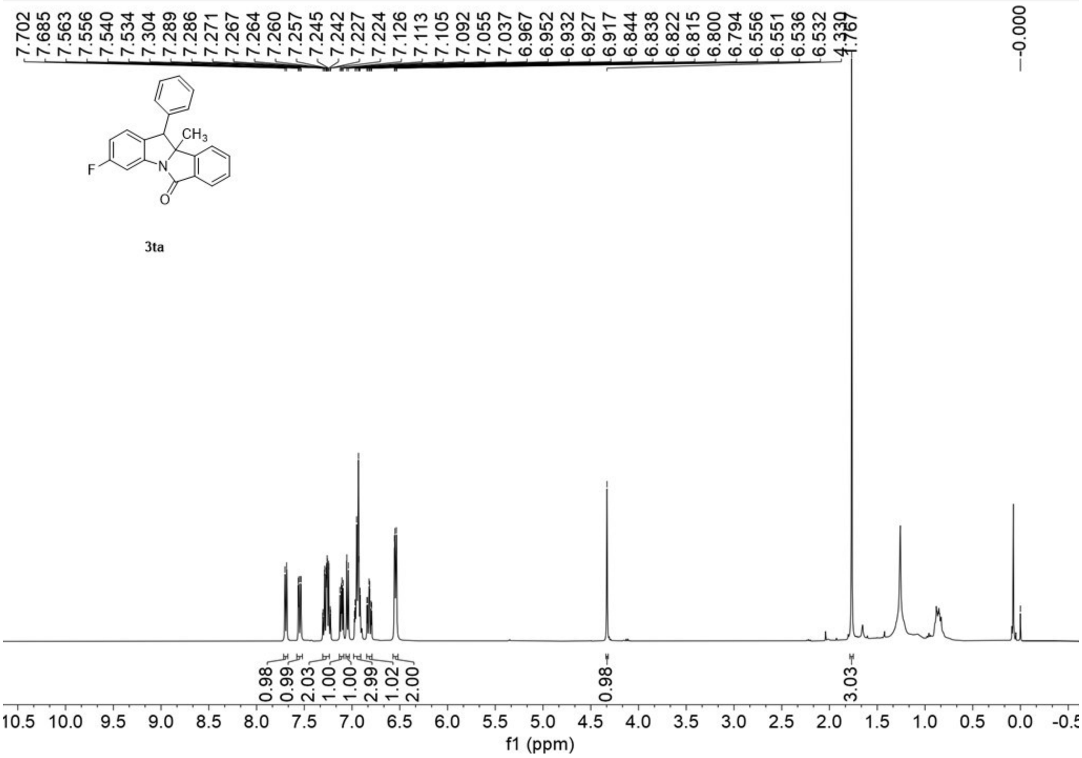


$^{13}$C-NMR spectrum ($CDCl_3$, 100 MHz) of **3ta**

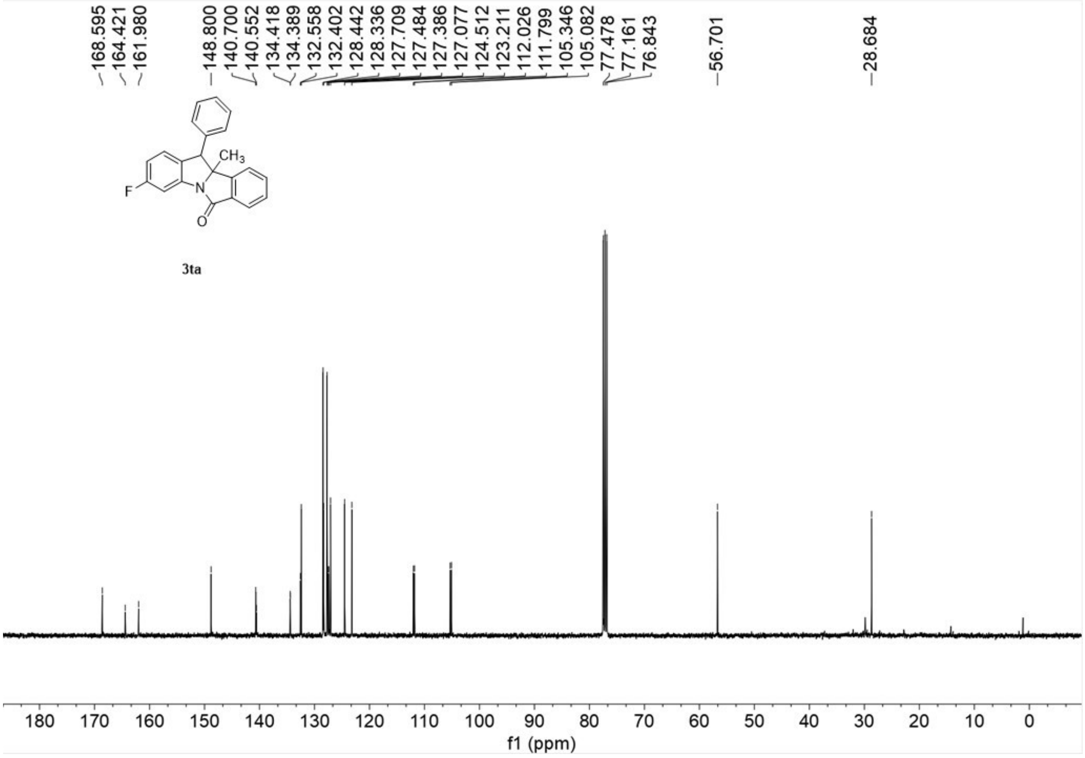

$^{19}$F-NMR spectrum ($CDCl_3$, 377 MHz) of **3ta**

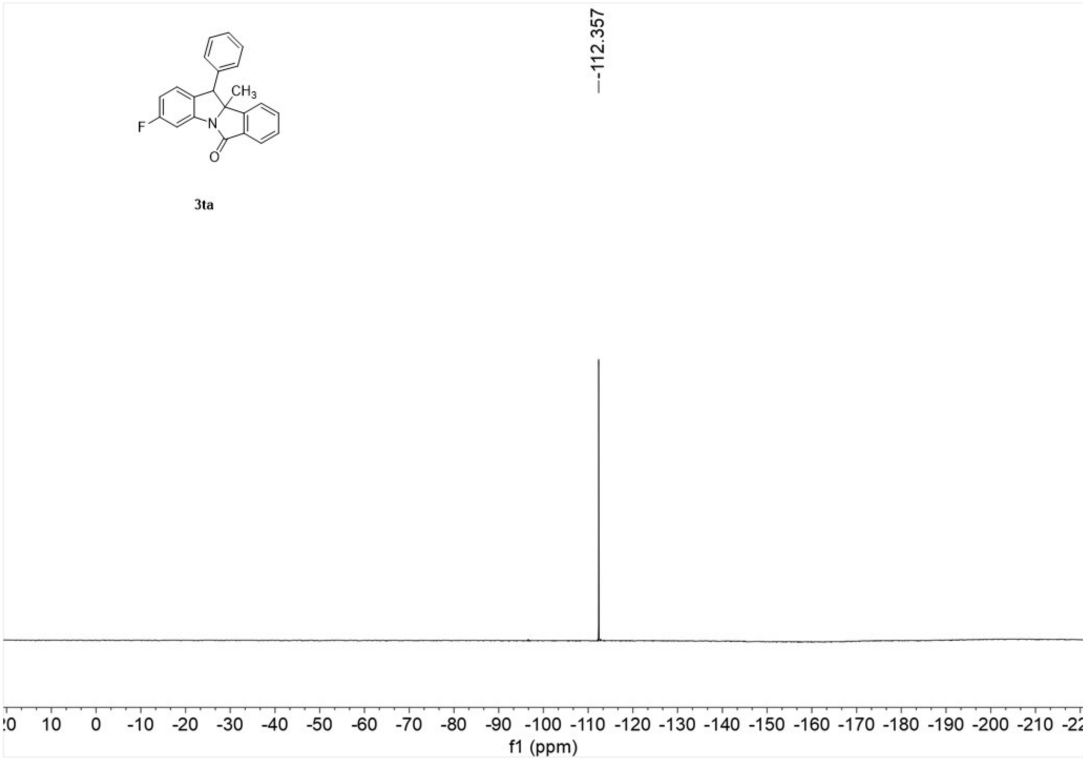


$^{1}$H-NMR spectrum ($CDCl_3$, 400 MHz) of **3ua**

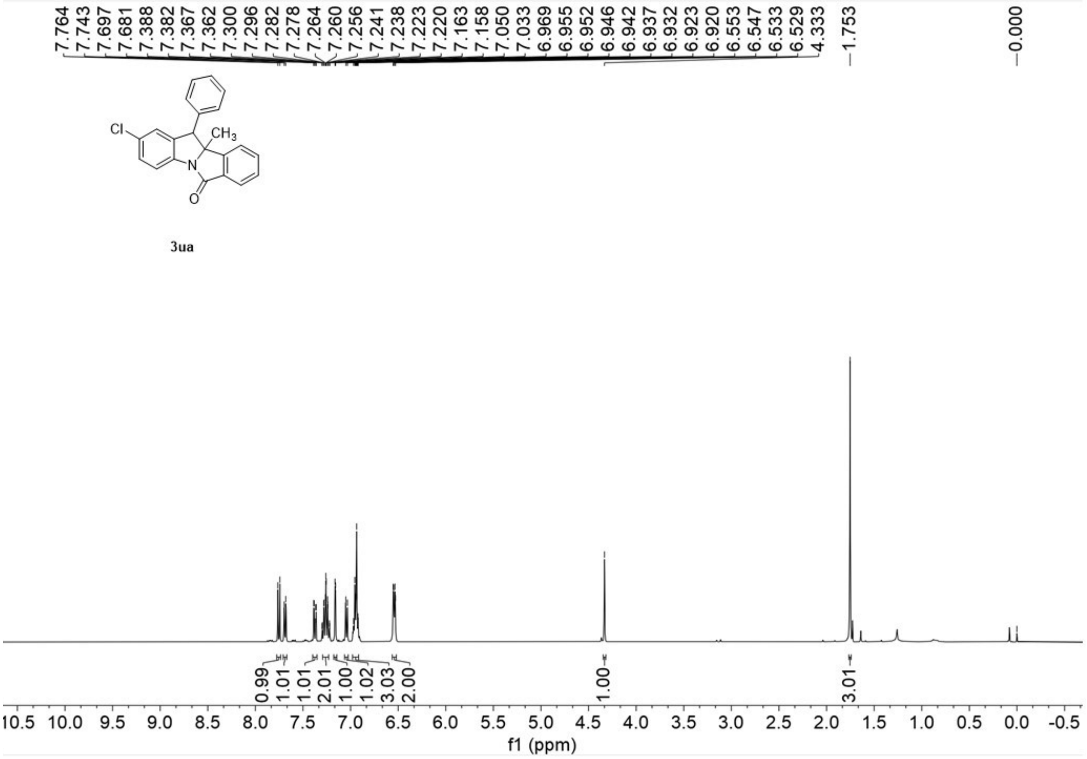

$^{13}$C-NMR spectrum ($CDCl_3$, 100 MHz) of **3ua**

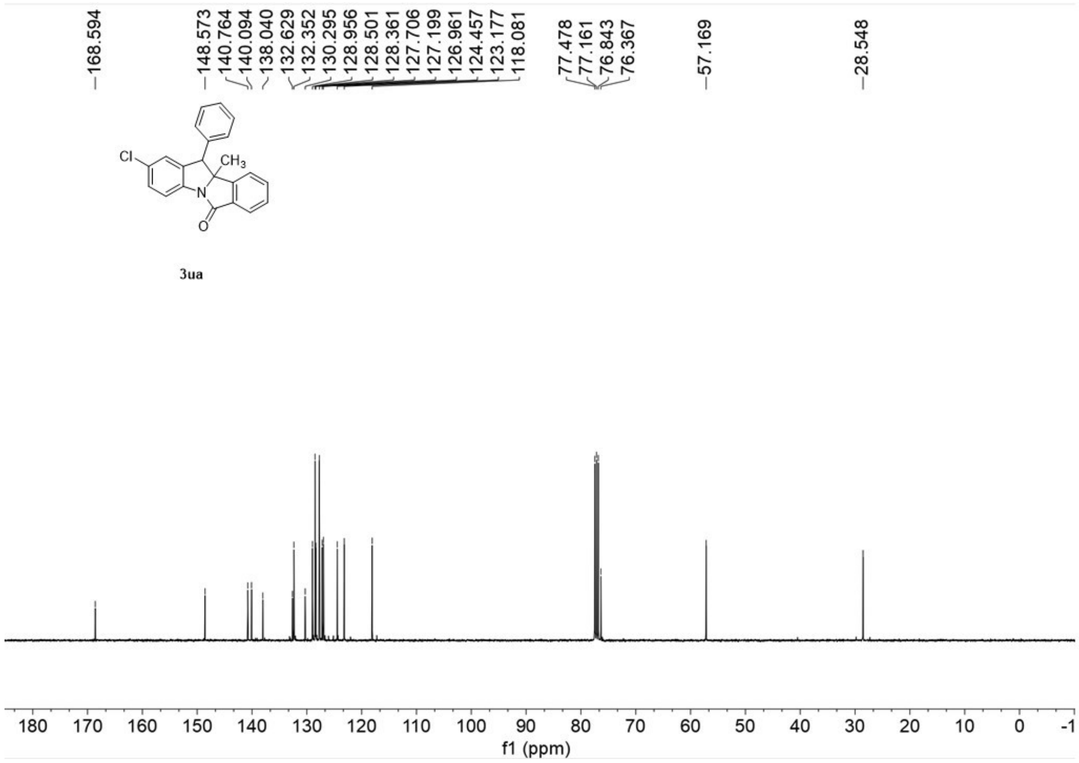


$^{1}$H-NMR spectrum ($CDCl_3$, 400 MHz) of **3ab**

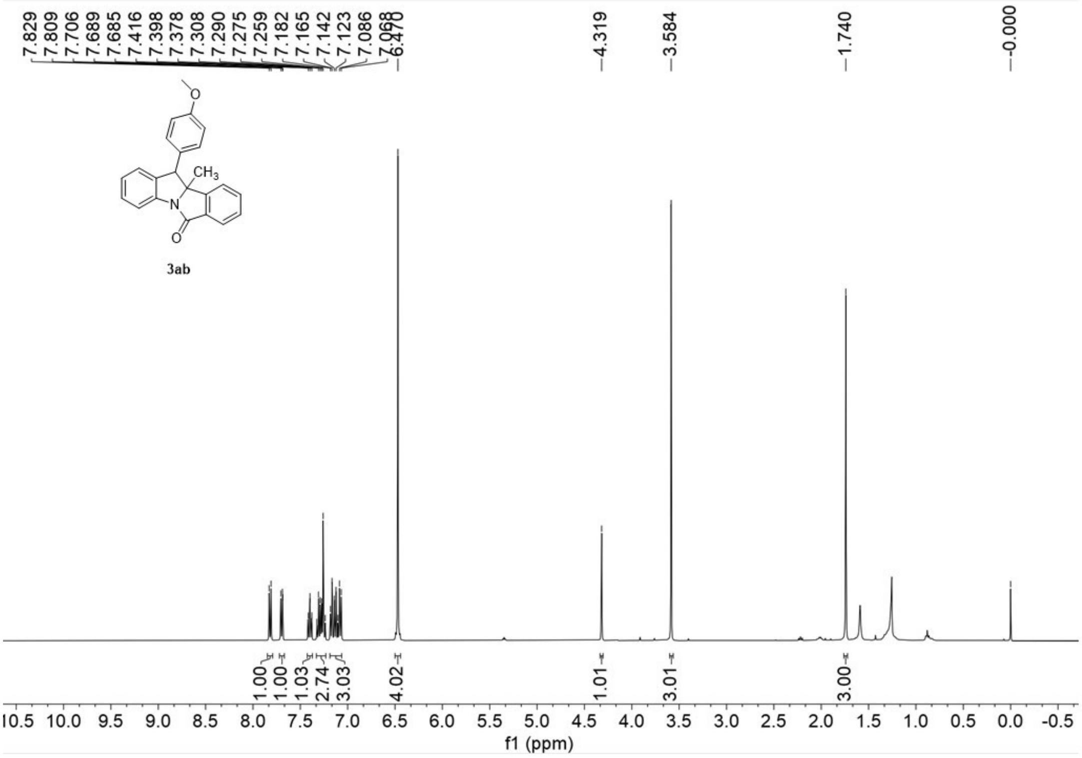

$^{13}$C-NMR spectrum ($CDCl_3$, 100 MHz) of **3ab**

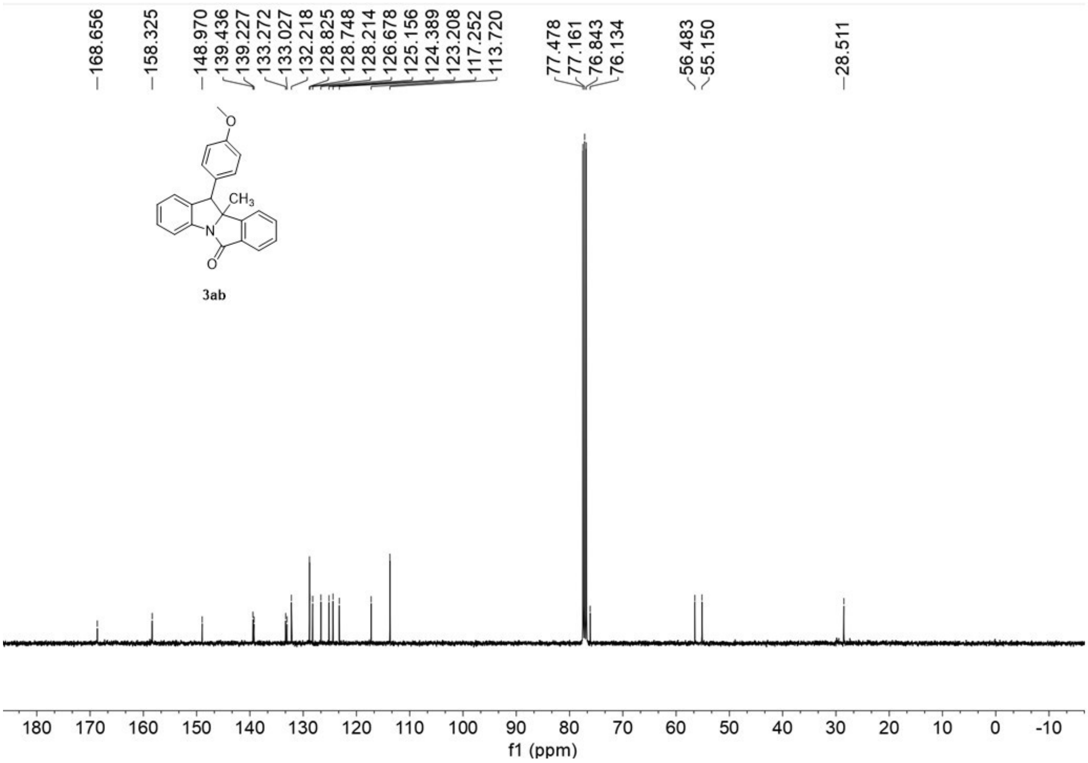


$^{1}$H-NMR spectrum ($CDCl_3$, 400 MHz) of **3ac**

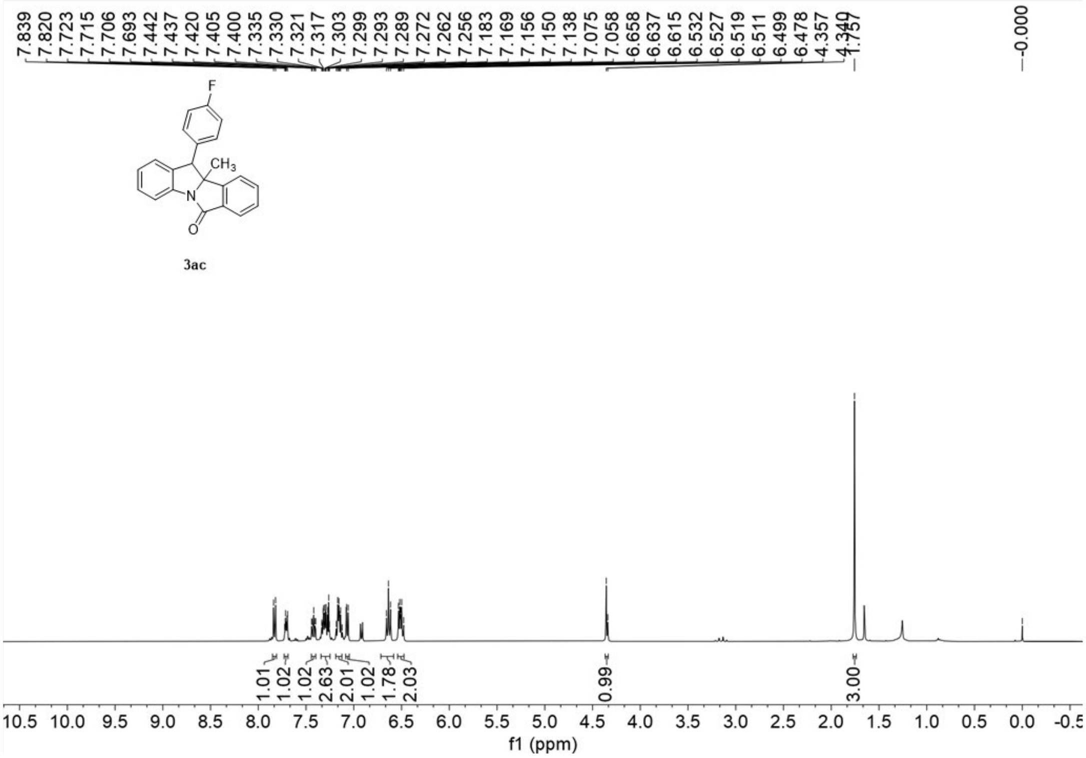

$^{13}$C-NMR spectrum ($CDCl_3$, 100 MHz) of **3ac**

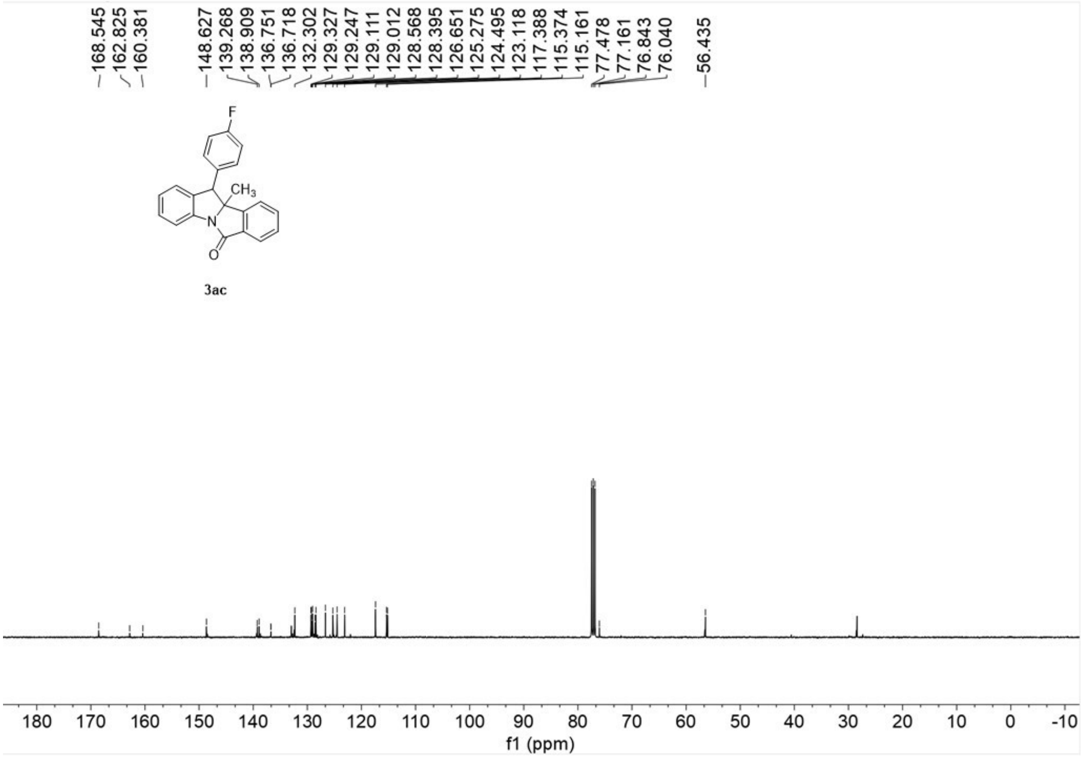


$^{19}$F-NMR spectrum ($CDCl_3$, 377 MHz) of **3ac**

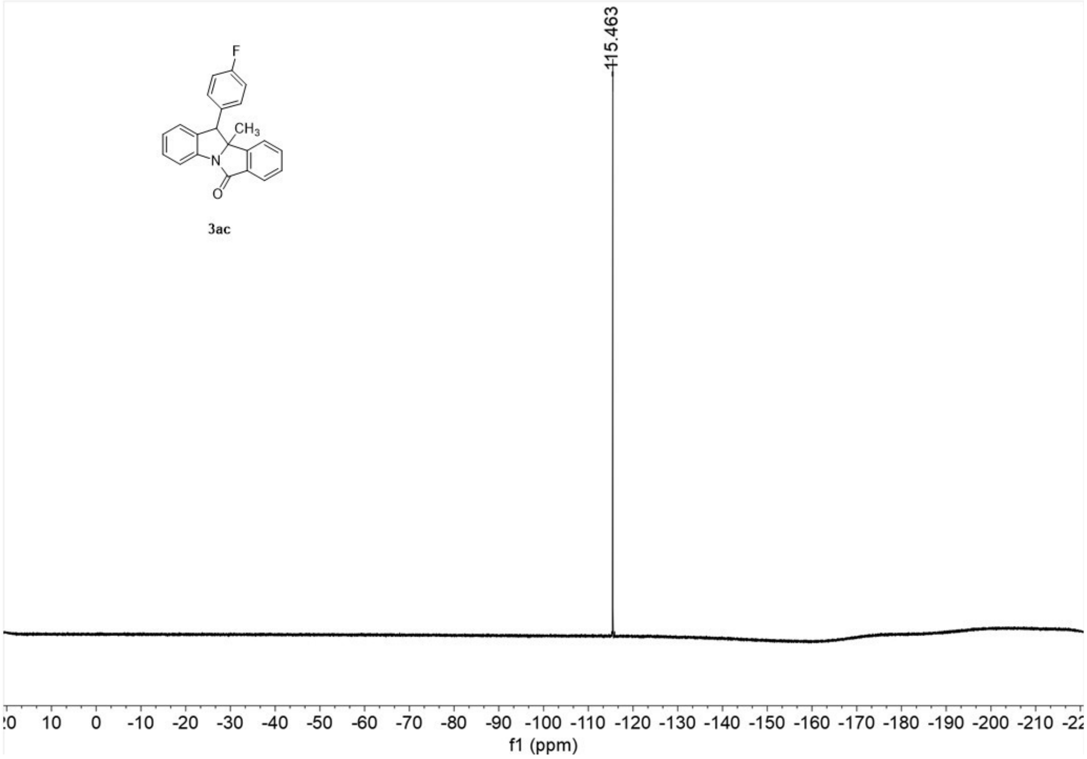

$^{1}$H-NMR spectrum ($CDCl_3$, 400 MHz) of **3ad**

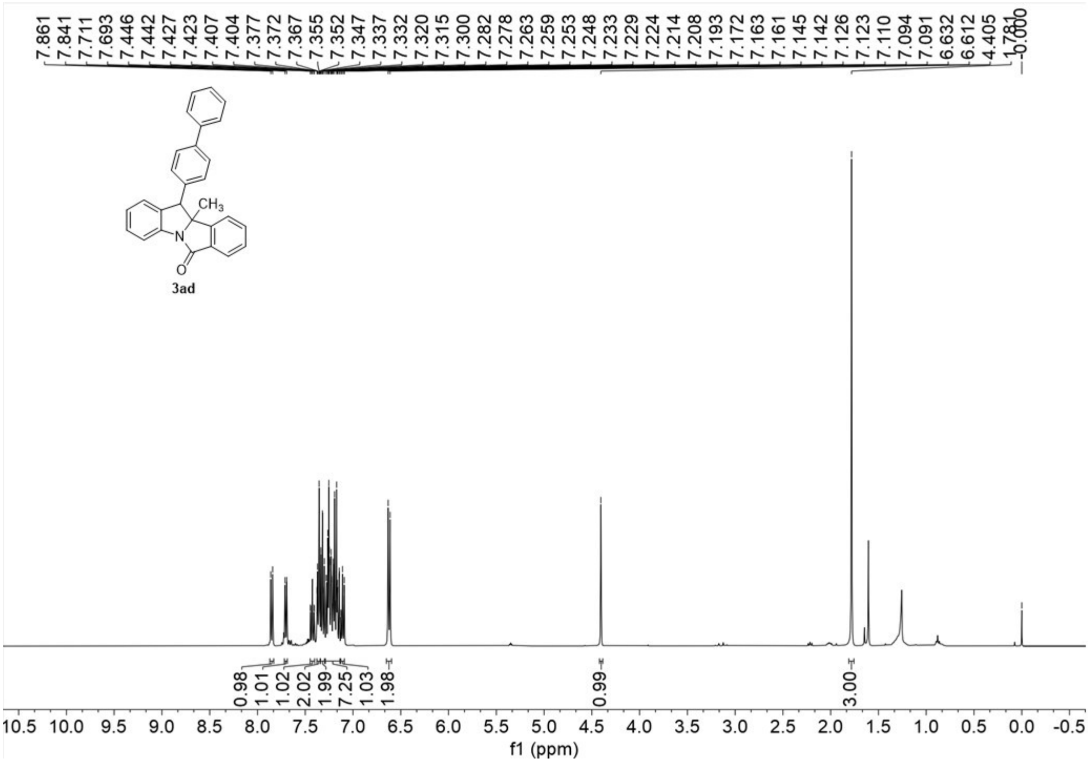

$^{13}$C-NMR spectrum ($CDCl_3$, 100 MHz) of **3ad**

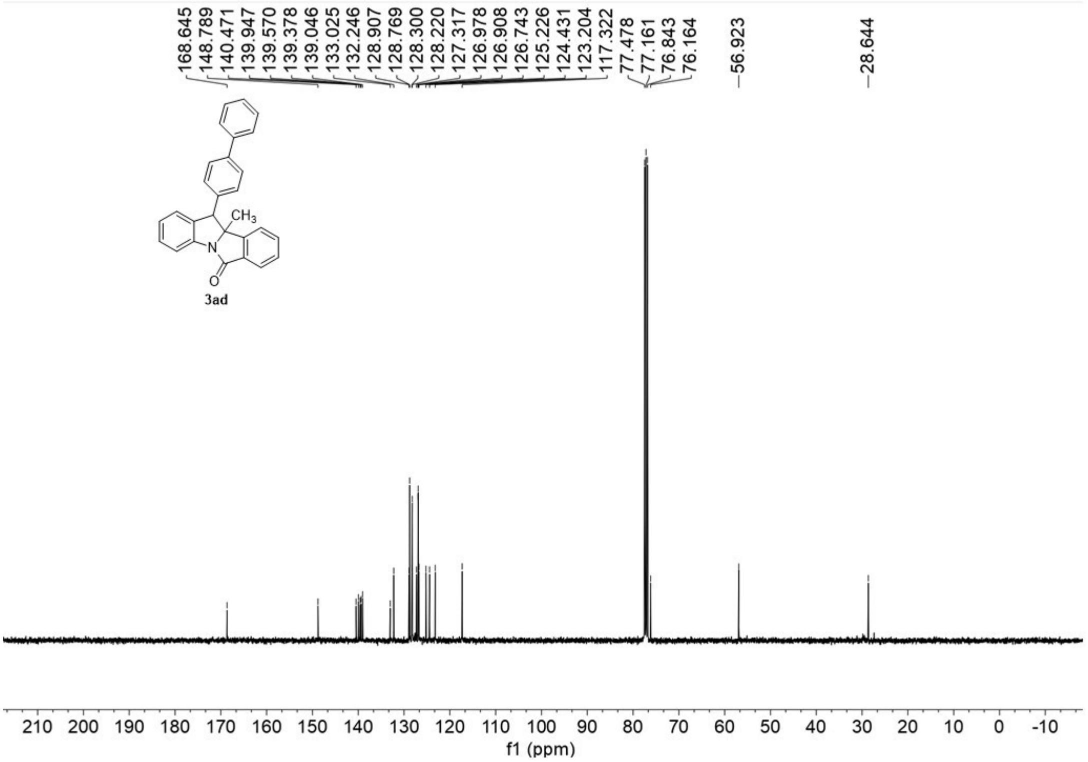

$^{1}$H-NMR spectrum ($CDCl_3$, 600 MHz) of **3ae**

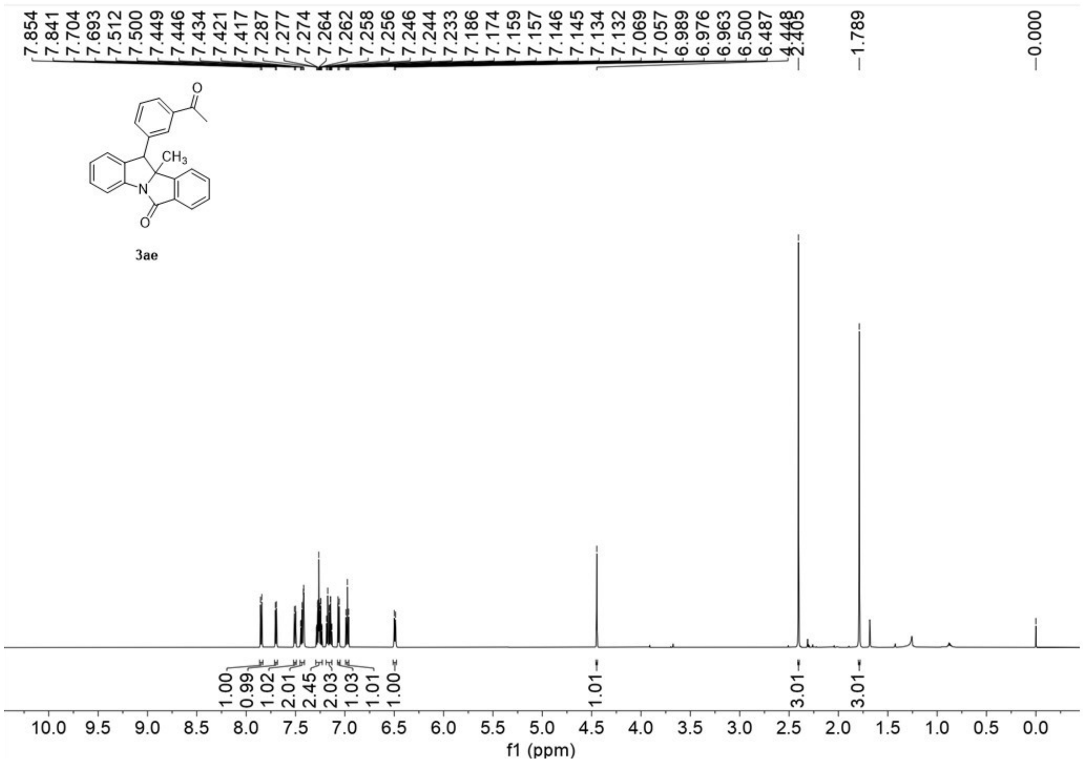


$^{13}$C-NMR spectrum ($CDCl_3$, 150 MHz) of **3ae**

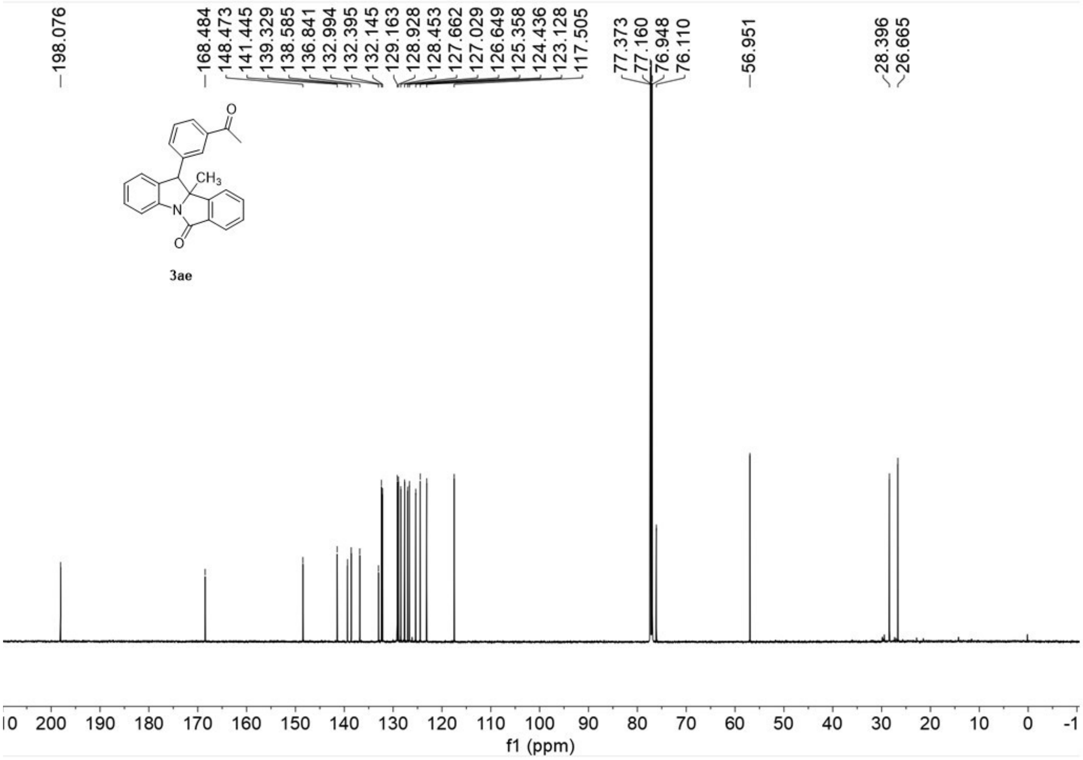

$^{1}$H-NMR spectrum ($CDCl_3$, 400 MHz) of **3af**

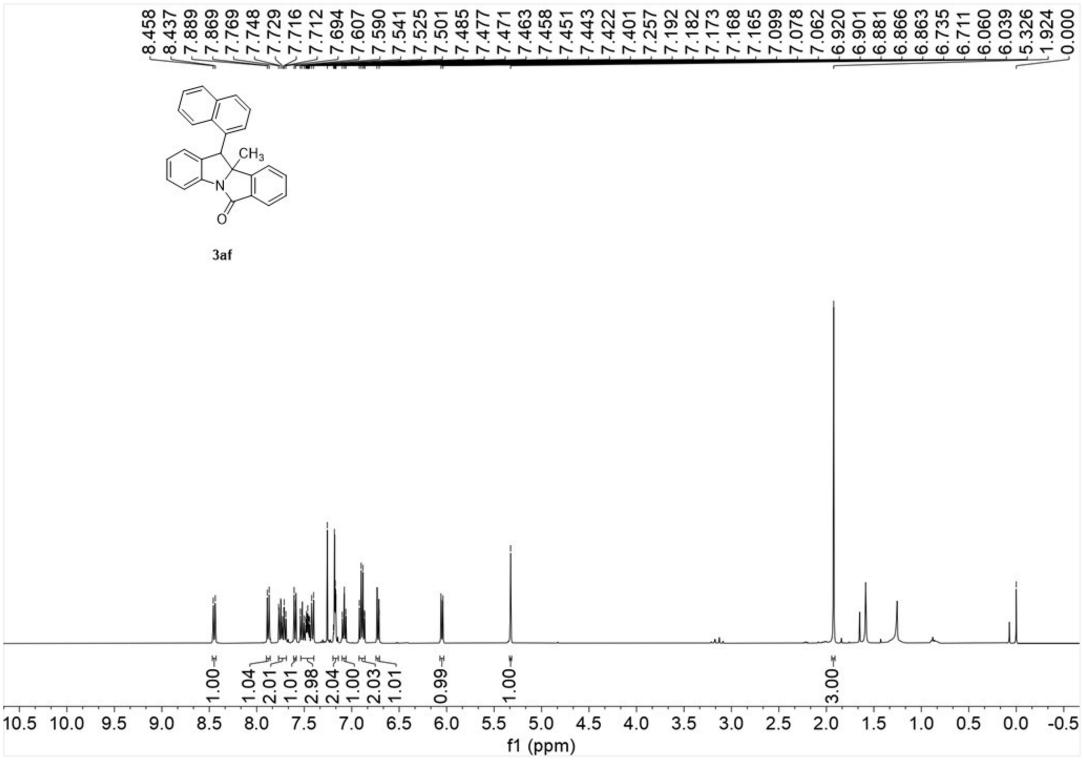


$^{13}$C-NMR spectrum ($CDCl_3$, 100 MHz) of **3af**

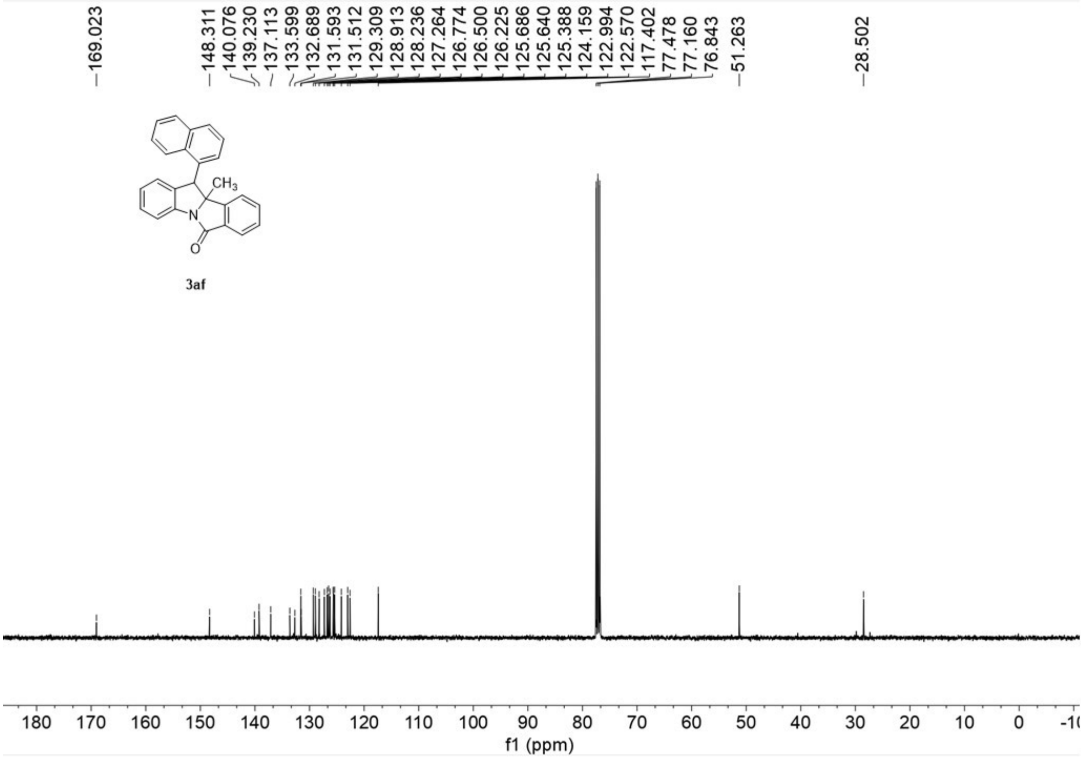

$^{1}H$-NMR spectrum ($CDCl_3$, 400 MHz) of **3ag**

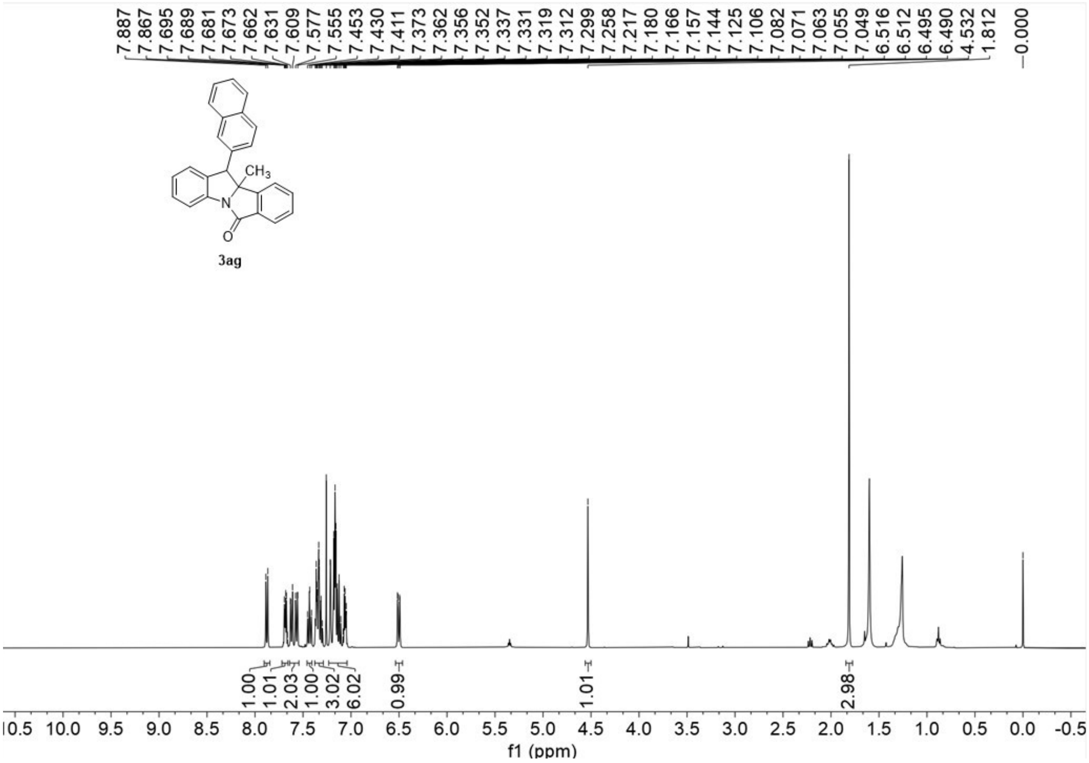

$^{13}C$-NMR spectrum ($CDCl_3$, 100 MHz) of **3ag**

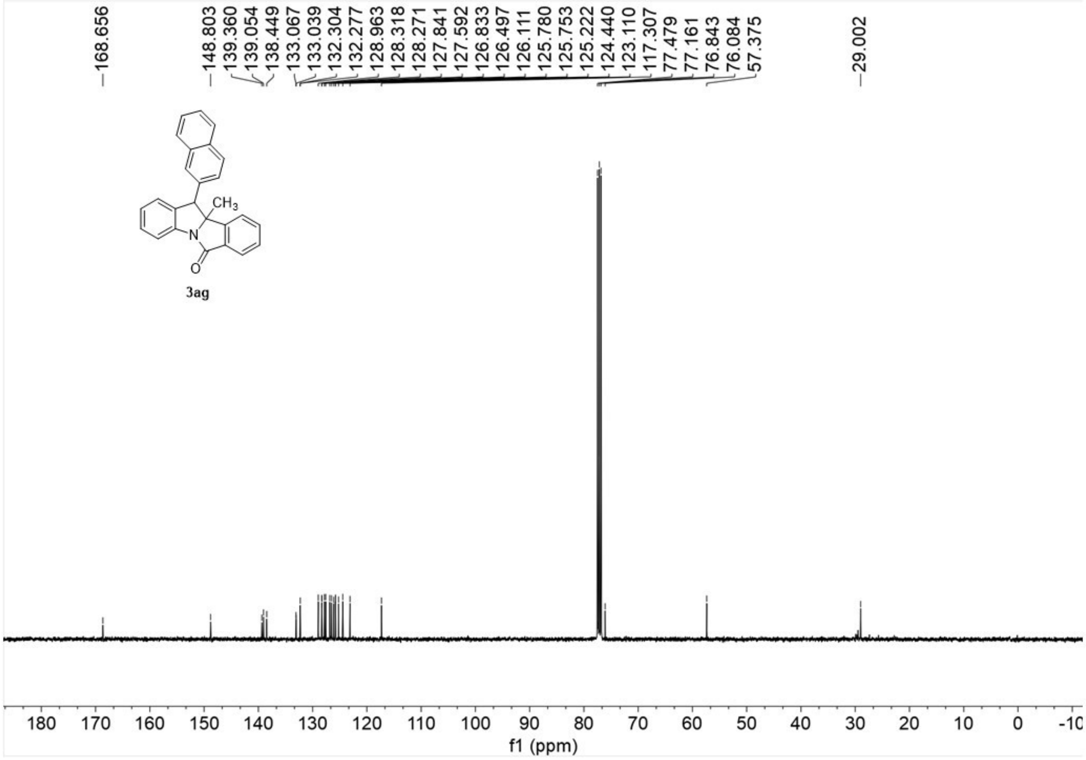

$^{1}$H-NMR spectrum ($CDCl_3$, 400 MHz) of **3ah**

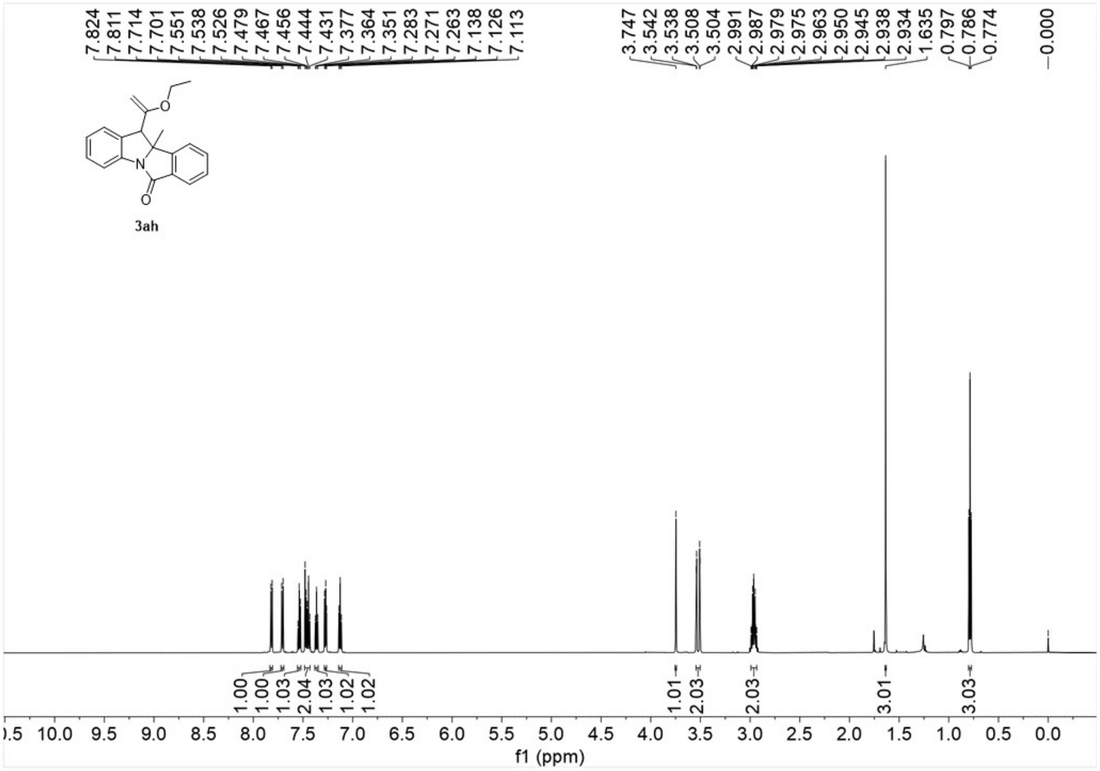


$^{13}$C-NMR spectrum ($CDCl_3$, 100 MHz) of **3ah**

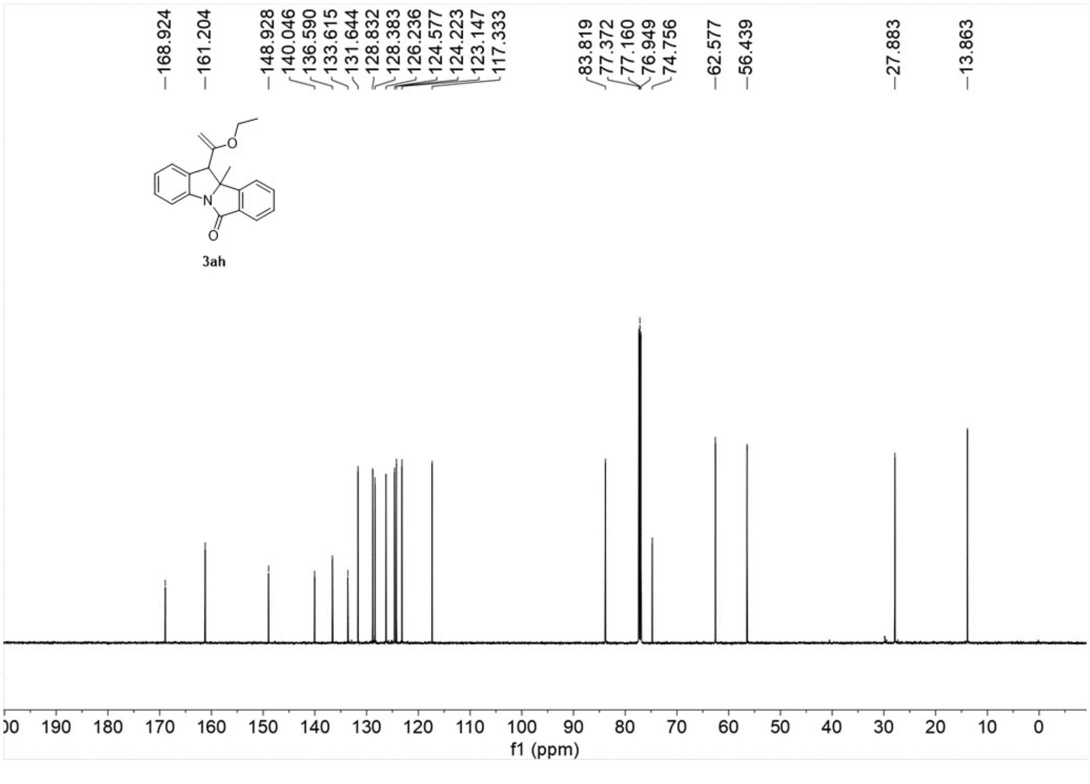

$^{1}$H-NMR spectrum ($CDCl_3$, 400 MHz) of **3ai**

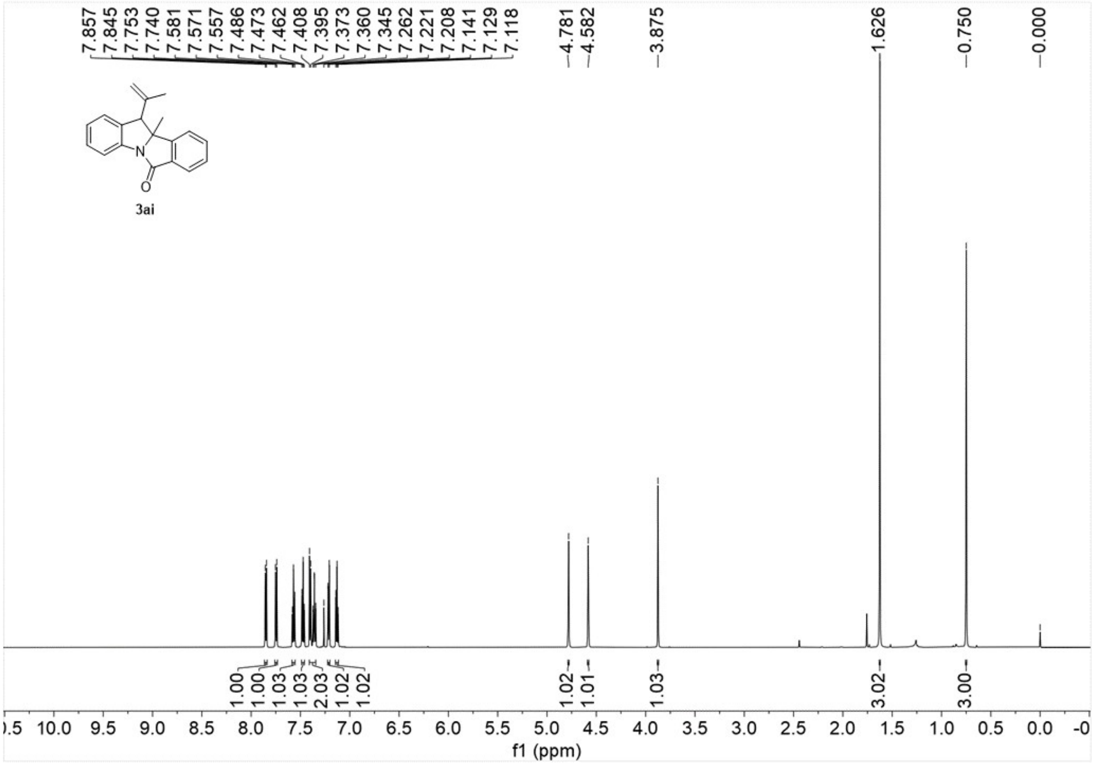

$^{13}$C-NMR spectrum ($CDCl_3$, 100 MHz) of **3ai**

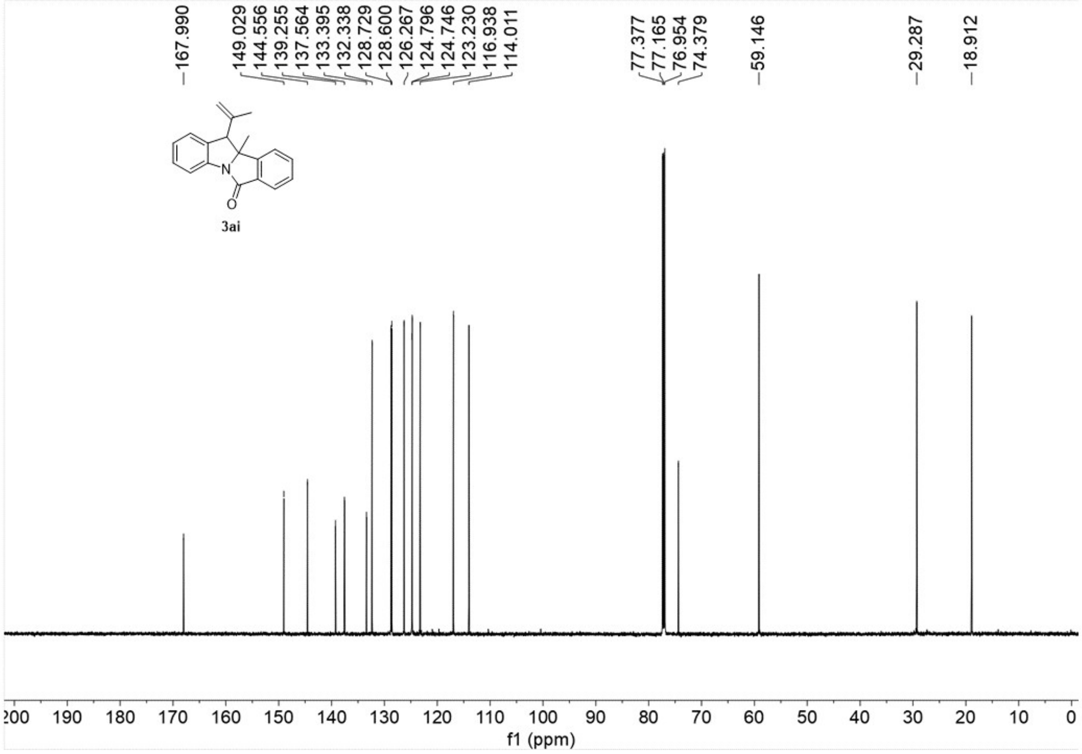

$^{1}$H-NMR spectrum ($CDCl_3$, 400 MHz) of **3aj**

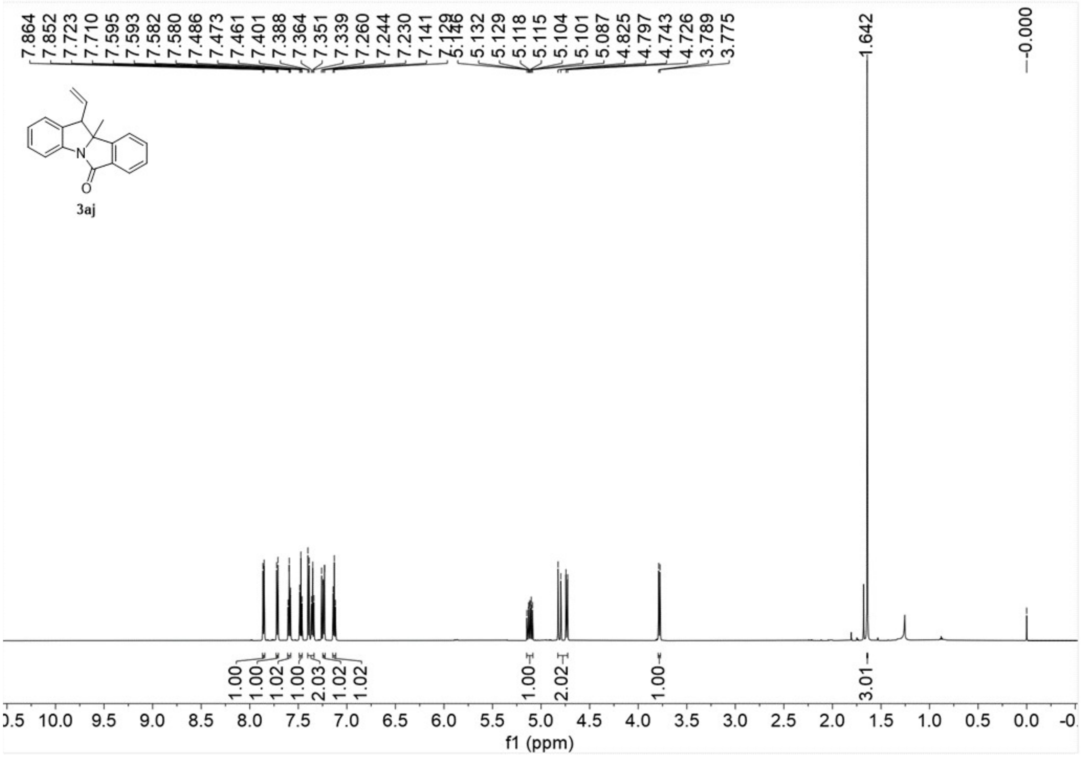


$^{13}$C-NMR spectrum ($CDCl_3$, 100 MHz) of **3aj**

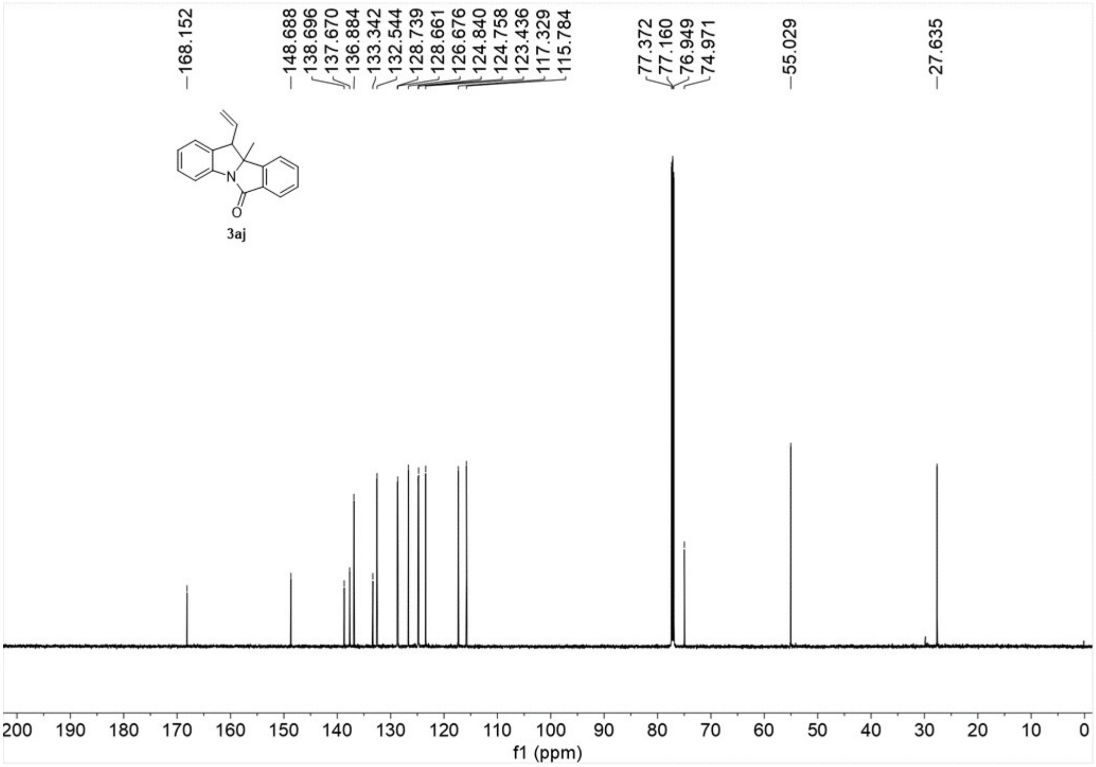

$^{1}$H-NMR spectrum ($CDCl_3$, 400 MHz) of **3ak**

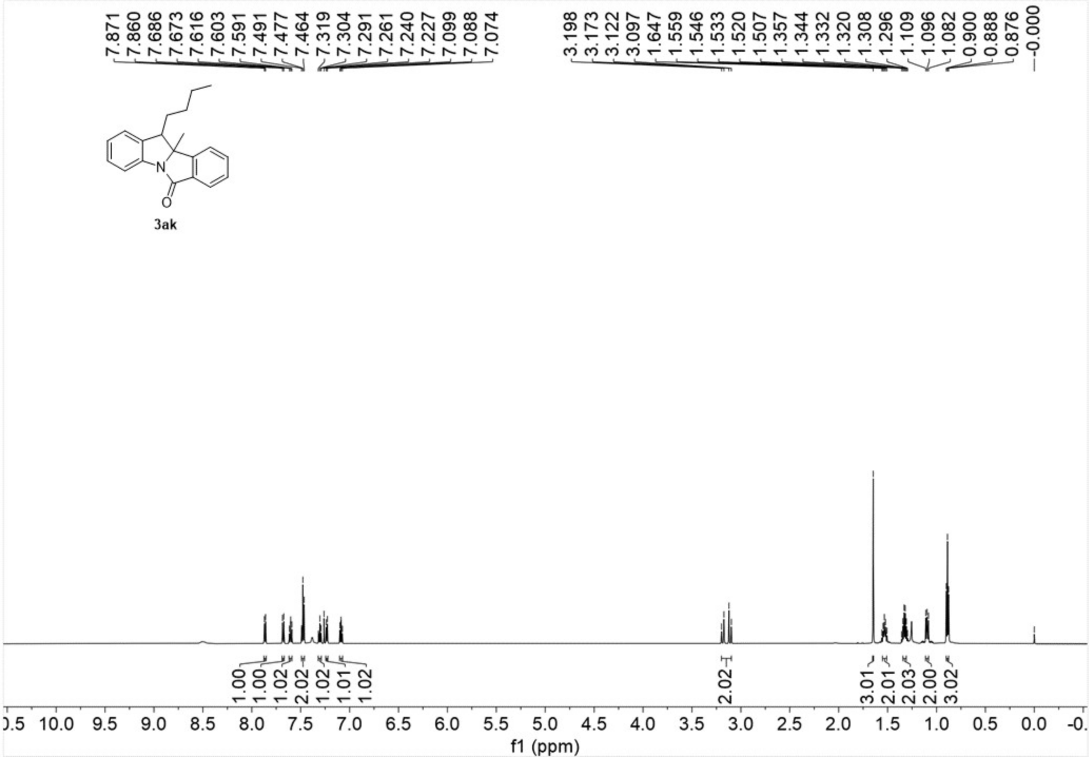

$^{13}$C-NMR spectrum ($CDCl_3$, 100 MHz) of **3ak**

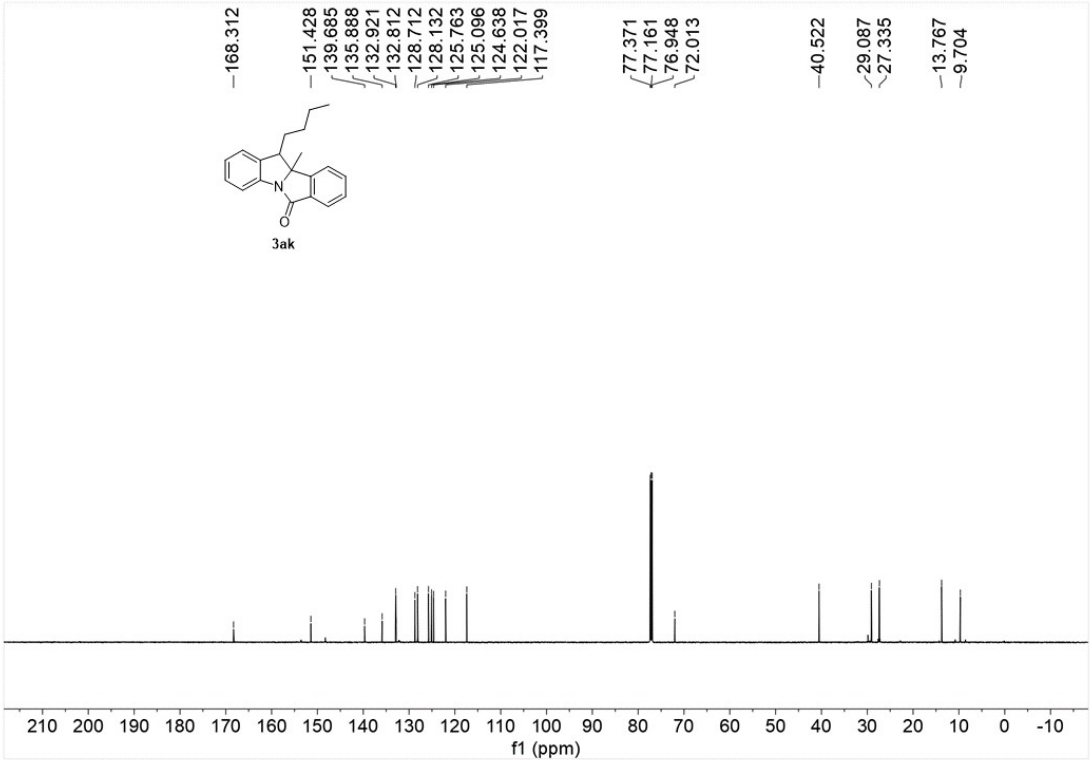

Supplement: RA-016-D6RA02185A-s001 [file RA-016-D6RA02185A-s001.pdf]
